# Interferon-Alpha Reduces Human Hippocampal Neurogenesis and Increases Apoptosis via Activation of Distinct STAT1-Dependent Mechanisms

**DOI:** 10.1093/ijnp/pyx083

**Published:** 2017-10-10

**Authors:** Alessandra Borsini, Annamaria Cattaneo, Chiara Malpighi, Sandrine Thuret, Neil A Harrison, MRC ImmunoPsychiatry Consortium, Patricia A Zunszain, Carmine M Pariante

**Affiliations:** 1 Section of Stress, Psychiatry and Immunology and Perinatal Psychiatry, King’s College London, London, United Kingdom; 2 Institute of Psychiatry, Psychology and Neuroscience, Department of Psychological Medicine, London, United Kingdom; 3 IRCCS Fatebenefratelli Institute, Biological Psychiatry Laboratory, Brescia, Italy; 4 King’s College London, Institute of Psychiatry, Psychology and Neuroscience, Department of Basic and Clinical Neuroscience, London, United Kingdom; 5 University of Sussex, Department of Neuroscience, Brighton and Sussex Medical School, Brighton, United Kingdom

**Keywords:** apoptosis, depression, inflammation, interferon-alpha, neurogenesis

## Abstract

**Background:**

In humans, interferon-α treatment for chronic viral hepatitis is a well-recognized clinical model for inflammation-induced depression, but the molecular mechanisms underlying these effects are not clear. Following peripheral administration in rodents, interferon-α induces signal transducer and activator of transcription-1 (STAT1) within the hippocampus and disrupts hippocampal neurogenesis.

**Methods:**

We used the human hippocampal progenitor cell line HPC0A07/03C to evaluate the effects of 2 concentrations of interferon-α, similar to those observed in human serum during its therapeutic use (500 pg/mL and 5000 pg/mL), on neurogenesis and apoptosis.

**Results:**

Both concentrations of interferon-α decreased hippocampal neurogenesis, with the high concentration also increasing apoptosis. Moreover, interferon-α increased the expression of interferon-stimulated gene 15 (ISG15), ubiquitin-specific peptidase 18 (USP18), and interleukin-6 (IL-6) via activation of STAT1. Like interferon-α, co-treatment with a combination of ISG15, USP18, and IL-6 was able to reduce neurogenesis and enhance apoptosis via further downstream activation of STAT1. Further experiments showed that ISG15 and USP18 mediated the interferon-α-induced reduction in neurogenesis (potentially through upregulation of the ISGylation-related proteins UBA7, UBE2L6, and HERC5), while IL-6 mediated the interferon-α-induced increase in apoptosis (potentially through downregulation of aquaporin 4). Using transcriptomic analyses, we showed that interferon-α regulated pathways involved in oxidative stress and immune response (e.g., Nuclear Factor (erythroid-derived 2)-like 2 [Nrf2] and interferon regulatory factor [IRF] signaling pathway), neuronal formation (e.g., CAMP response element-binding protein [CREB] signaling), and cell death regulation (e.g., tumor protein(p)53 signaling).

**Conclusions:**

We identify novel molecular mechanisms mediating the effects of interferon-α on the human hippocampus potentially involved in inflammation-induced neuropsychiatric symptoms.

**Significance Statement**

In humans, IFN-α treatment for chronic viral hepatitis is a well-recognized clinical model for inflammation-induced depression, but the molecular mechanisms underlying these effects are not clear. The present in vitro study shows that IFN-α reduces human neurogenesis and enhances human cell death in human hippocampal progenitor cells. We also identify novel molecular mechanisms that underlie these effects and that are thus potentially relevant for inflammation-induced neuropsychiatric symptoms. In particular, we demonstrate that IFN-α upregulates three IFN-α-stimulated genes, ISG15, USP18, and IL-6, via STAT1-dependent mechanisms and that those molecules are ultimately responsible for the effects seen upon in vitro treatment with IFN-α. Considering the significant involvement of IFN-α in neuropsychiatric and neurological disorders, this study offers a useful in vitro model on the potential roles of the hippocampus in the IFN-α-induced psychopathology and provides a better understanding of the molecular mechanisms regulated by IFN-α and potentially mediating inflammation-induced depression.

## Introduction

Initially identified for its anti-viral properties, interferon (IFN)-α is a pleiotropic molecule and a major mediator of innate and adaptive immune responses (Pestka, 2000). In addition to its immuno-modulatory properties, IFN-α has been shown to have a number of effects on the brain, ranging from increased neurotoxicity (Alboni et al., 2013) to the development of behavioral phenotypes, in both human and animal models (Felger et al., 2007; Hepgul et al., 2016a). In particular, IFN-α treatment has been shown to cause depressive symptoms as well as other neuropsychiatric adverse effects, such as anxiety, sleep disorders, and memory impairment, when administered to patients as a treatment for chronic viral hepatitis (Raison et al., 2005). However, the molecular mechanisms underlying these effects have not been completely elucidated yet.

Studies in rodents have shown that peripheral IFN-α induces changes in the expression of IFN-stimulated genes (ISGs) within discrete brain regions, including the hippocampus (Wang et al., 2008). Previous research has also demonstrated that peripheral administration of IFN-α alters neurogenesis in the hippocampus of adult rodents in terms of both cell proliferation and neuronal differentiation (Zheng et al., 2014). Taken together, these findings indicate that the hippocampus may be affected by IFN-α and that the psychiatric side effects caused by IFN-α therapy might be a consequence of disrupted neurogenesis or neuronal function in the hippocampus. Indeed, there is evidence that IFN-α and other cytokines can penetrate the more permeable areas of the blood-brain barrier to affect brain signaling (Zheng et al., 2014). Moreover, some other cytokines have been shown to alter neurogenesis (Borsini et al., 2015). In humans, adult neurogenesis principally occurs in the subventricular zone of the lateral ventricles and the subgranular zone of the dentate gyrus of the hippocampus (Kempermann et al., 2015). To our knowledge, there have been no studies investigating the effects of IFN-α on human hippocampal neurogenesis or examining the potential molecular mechanisms underlying these effects.

Previous data have shed light on some molecular pathways activated by IFN-α in the brain and highlighted their potential involvement in the development of IFN-α-induced psychopathology. IFN-α binds to its interferon alpha/beta receptor, leading to the phosphorylation of 2 transcription factors, STAT1 and STAT2. Phosphorylated STAT1 and STAT2 then migrate into the nucleus and induce the expression of ISGs, which mediates the biological effects of IFN-α (Wang et al., 2008). Indeed, we have recently shown that the IFN-stimulated genes, ISG15 and ubiquitin-specific peptidase 18 (USP18), are among the most upregulated genes in the blood of patients following in vivo IFN-α treatment (Hepgul et al., 2016b). Similarly, via activation of STAT signaling pathways, IFN-α can induce inflammatory cytokines, including IL-6 (Taga and Kishimoto, 1997). Indeed, there is in vitro evidence that neurons in the hippocampus of rodents respond to IFN-α with an increase in ISGs, such as STAT1, ISG15, USP18, and IL-6 (Wang et al., 2008; Jin et al., 2012).

To dissect the molecular mechanisms underlying the putative effects of IFN-α on human hippocampal neurogenesis, we here analyze the effect of 2 distinct concentrations of IFN-α (500 and 5000 pg/mL, similar to serum concentrations of patients receiving IFN-α for chronic viral hepatitis) (Bruno et al., 2004; Francois et al., 2010) on an immortalized human hippocampal progenitor cell line previously established as an in vitro model of human neurogenesis (Zunszain et al., 2012; Anacker et al., 2013a). We also test the ability of IFN-α to induce STAT1, ISG15, USP18, and IL-6 in these cells and investigate whether these proteins are mechanistically involved in the effects of IFN-α. Finally, we conduct transcriptomic analyses to shed light on novel molecular signaling pathways regulated by IFN-α in hippocampal neurons.

## Methodology

### Cell Culture

We used our established in vitro model of human hippocampal neurogenesis, the multipotent human hippocampal progenitor cell line HPC0A07/03C (provided by ReNeuron, Surrey, UK) (Anacker et al., 2011, 2013a, 2013b; Zunszain et al., 2012; Horowitz et al., 2015; Borsini et al., 2017). This model was previously validated using a hippocampal newborn neuron specific marker, Prospero homeobox protein 1 (Anacker et al., 2013a). Cells were left to proliferate in the presence of growth factors (Epidermal Growth Factor [EGF], basic Fibroblast Growth Factor [bFGF]) and 4-hydroxytamoxifen (4-OHT). Differentiation was initiated by removal of the growth factors and 4-OHT. Detailed information on this cell line and culture conditions can be found in our previous publications and in the supplementary Materials.

### Differentiation Assays

To assess changes in neuronal differentiation, HPC0A07/03C cells were plated into clear 96-well plates (Nunclon) at a density of 1.2x10^4^ cells/well. Twenty-four hours later, cells were cultured in the presence of EGF, bFGF, and 4-OHT and with IFN-α, or other proteins/drugs for 3 days (see supplementary Materials). After this initial proliferation phase, cells were washed and then cultured in media containing IFN-α or the same proteins/compounds but without growth factors or 4-OHT for 7 subsequent days. This paradigm was used for all experiments. Finally, cells were rinsed with warm PBS and fixed with 4% paraformaldehyde (PFA) for 20 minutes at room temperature.

### Immunocytochemistry

Differentiation into immature and mature neurons was assessed with doublecortin (DCX) (Alexa 488 donkey anti-rabbit; 1:1000) and microtubule-associated protein 2 (MAP2) (Alexa donkey 555 anti-mouse, 1:1000, Invitrogen), respectively. Apoptotic cells were examined using caspase 3 (CC3) (Alexa 488 donkey anti-rabbit; 1:1000; Invitrogen). DAPI dye was used to label all cells. The number of positive cells over total DAPI positive cells was counted using an insight automated imaging platform (CellInsight) (see supplementary Materials). See supplementary Figure 1 for representative images.

### Multiplex Cytokines Measurement

For cytokines measurement, cell supernatants of differentiated cells were run on the Human ProInflammatory Multileplex Very-Sensitive Kit from Meso Scale Discovery according to the manufacturers’ instructions (see supplementary Materials) and analyzed on the SECTOR Imager Meso Scale Discovery device.

### RNA Isolation, cDNA Synthesis, and Quantitative Real-Time PCR (qPCR) Analysis

RNA of differentiated cells was isolated using the RNeasy Micro Kit (Qiagen) following the manufacturer’s instructions and frozen at -80°C. Then 1 µg total RNA was reversed-transcribed by Superscript III enzyme (Life Technologies) according to the manufacturers’ instructions (see supplementary Materials). Subsequently, both target and housekeeping gene expression levels were analyzed by TaqMan qRT-PCR instrument (CFX384 real time system, Bio-Rad) using the iScript one-step RTPCR kit for probes (Bio-Rad) (see supplementary Materials).

### Microarray Analysis

Microarray assays were performed following the protocol in the Affymetrix GeneChip Expression Analysis technical manual (Affymetrix), as previously described (Anacker et al., 2013a). For gene expression data, CEL files were imported into Partek Genomics Suite V6.6 for data visualization and quality control and statistical analyses. Ingenuity Pathway Analysis (IPA) Software was used to identify regulation of molecular signaling pathways. Seventeen genes were validated using Real Time PCR analyses, as described above.

### Statistical Analysis

Each experiment was replicated in at least 3 independent cultures (biological replicates). All statistical analyses were performed with GraphPad Prism 6.00. One-way ANOVA with Newman–Keuls’s posthoc test was used for multiple comparisons among treatment groups. Data are presented as mean*±*SEM, and *P* values<.05 were considered significant.

## Results

### IFN-α Decreases Neurogenesis and Increases Apoptosis in Human Hippocampal Precursor Cells

**Figure 1.**
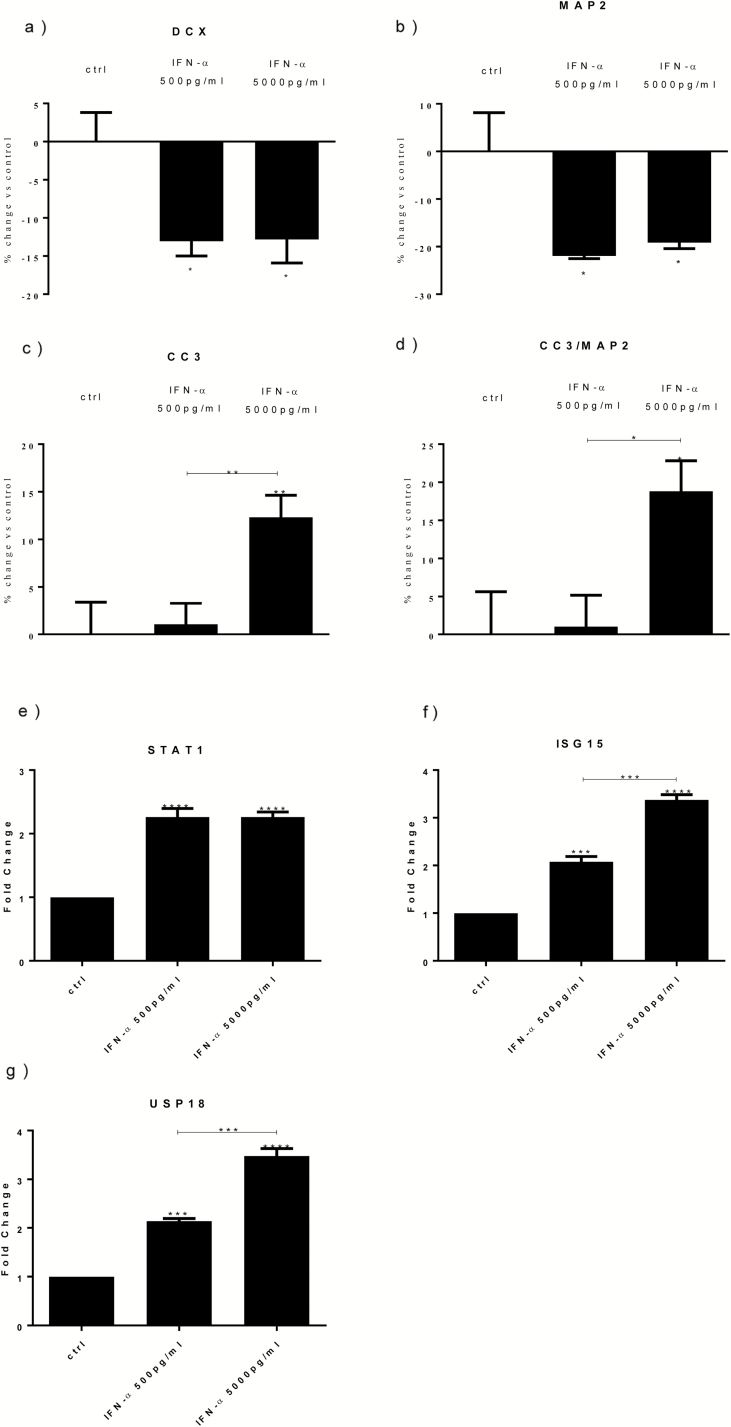
Interferon (IFN)-α reduces differentiation, increases apoptosis, and upregulates signal transducer and activator of transcription-1 (STAT1), interferon-stimulated gene 15 (ISG15), and ubiquitin-specific peptidase 18 (USP18) gene expression. IFN-α (500 and 5000 pg/mL) decreased doublecortin (DCX)+ (a) and microtubule-associated protein 2 (MAP2)+ cells (b). IFN-α 5000 pg/mL increased both caspase 3 (CC3)+ cells (c) and CC3+/MAP2+ cells (d). IFN-α (500 and 5000 pg/mL) increased STAT1 (e), ISG15 (f), and USP18 (g) gene expression. Three independent experiments were conducted on independent cultures (n=3). Data are shown as mean*±*SEM. 1-way ANOVA, Newman–Keuls’ posthoc test ^*^P<.05, ^**^P<.01, ^***^P<.001, ^****^P<.0001, compared with vehicle treatment or as indicated.

Cells were treated with IFN-α (500 pg/mL or 5000 pg/mL) over a period of 10 days (3 days of proliferation followed by 7 days of differentiation). We found a significant reduction in the number of DCX+ cells (immature neurons) by both concentrations of IFN-α (-12%; Figure 1a). We also found that the number of MAP2+ cells (mature neurons) was significantly reduced by both conditions (-21% and -18%, respectively) (Figure 1b).

IFN-α at 5000 pg/mL, but not at 500 pg/mL, also significantly increased the number of apoptotic cells (CC3+) and the number of apoptotic cells over the pool of newly mature neurons (CC3+/MAP2+) (respectively, +12%, Figure 1c; and +18%, Figure 1d).

### IFN-α Increases STAT1, ISG15, and USP18 mRNA Gene Expression, and IL-6 Protein

Previous evidence has reported that STAT1, ISG15, and USP18 are among the most upregulated genes in mouse neurons following stimulation with IFN-α (Wang et al., 2008) as well as in the blood of patients taking IFN-α (Hepgul et al., 2016b). We wanted to test whether in vitro treatment with IFN-α was also able to induce those genes in human hippocampal cells. We indeed demonstrated that both concentrations of IFN-α (500 and 5000 pg/mL) significantly increased mRNA gene expression of STAT1 (fold change [fc]=2.7 and 3.1, respectively), ISG15 (fc=2.1 and 3.3), and USP18 (fc=2.1 and 3.4) (Figure 1e-g).

In addition, we sought to characterize the effects of IFN-α on cytokine secretion in the supernatant of these cells. We focused our analysis on cytokines known to be regulated by IFN-α: 3 that are stimulated (IL-6, IL-8, and IFN-γ; Bonaccorso et al., 2001) and 2 that are inhibited (IL-10 and IL-13; Feng et al., 2002; Sugimoto et al., 2005). However, we found no effects of the low concentration of IFN-α (500 pg/mL) on any of the cytokines, which were all expressed at low levels in the supernatant (<3 pg/mL). At the high concentration of IFN-α (5000 pg/mL), only IL-6 protein expression was significantly upregulated compared with unstimulated cells (4.9 pg/mL*±*0.1 vs 1.7 pg/mL±0.03, *P*<.0001).

### Cotreatment with ISG15, USP18, and IL-6 Mimics the Effect of IFN-α on Neurogenesis and Apoptosis

To investigate the potential mechanisms involved in effects of IFN-α on neurogenesis and apoptosis, we focused on ISG15, USP18, and IL-6, the 3 molecules induced by treatment with IFN-α. Cells were cotreated with ISG15 (10 pg/μL), USP18 (10 pg/μL), or IL-6 (5 pg/μL) recombinant proteins, either alone or in combination (see supplementary Materials for justification of the concentrations).

**Figure 2.**
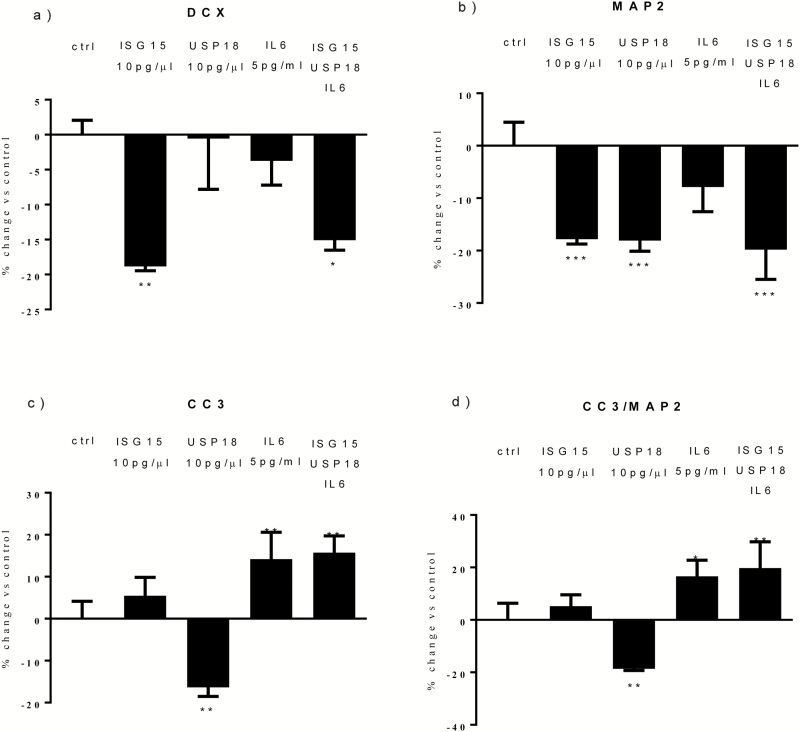
Cotreatment with interferon-stimulated gene 15 (ISG15), ubiquitin-specific peptidase 18 (USP18), and interleukin (IL)-6 mimics the effect of interferon (IFN)-α on neurogenesis and apoptosis. ISG15 and all proteins reduced doublecortin (DCX)+ cells (a). ISG15, USP18, and all proteins reduced microtubule-associated protein 2 (MAP2)+ cells (b). USP18 also decreased caspase 3 (CC3)+ cells, whereas IL-6 and all proteins significantly increased both CC3+ and CC3/MAP2+ cells (c-d). Three independent experiments were conducted on independent cultures (n=3). Data are shown as mean*±*SEM. 1-way ANOVA, Newman–Keuls’ posthoc test ^*^P<.05, ^**^P<.01, ^***^P<.001, compared with vehicle treatment or as indicated.

Similar to what we found with IFN-α, we found a significant reduction in DCX+ cells following cotreatment with all proteins (-15%, Figure 2a). Moreover, we found a significant reduction following treatment with ISG15 alone (-18%, Figure 2a), but not with USP18 or IL-6 alone (Figure 2a). This shows that ISG15, but not USP18 or IL-6, is involved in the IFN-α-induced reduction in DCX+ cells.

Again, similar to what we found with IFN-α, we also found a significant reduction in MAP2+ cells following cotreatment with all proteins (-19%, Figure 2b). Moreover, we found a significant reduction following treatment with ISG15 alone (-17%, Figure 2b) or USP18 alone (-18%, Figure 2b), but not with IL-6 alone (Figure 2b). This shows that ISG15 and USP18, but not IL-6, are involved in the IFN-α**–**induced reduction in MAP2+ cells.

**Figure 3.**
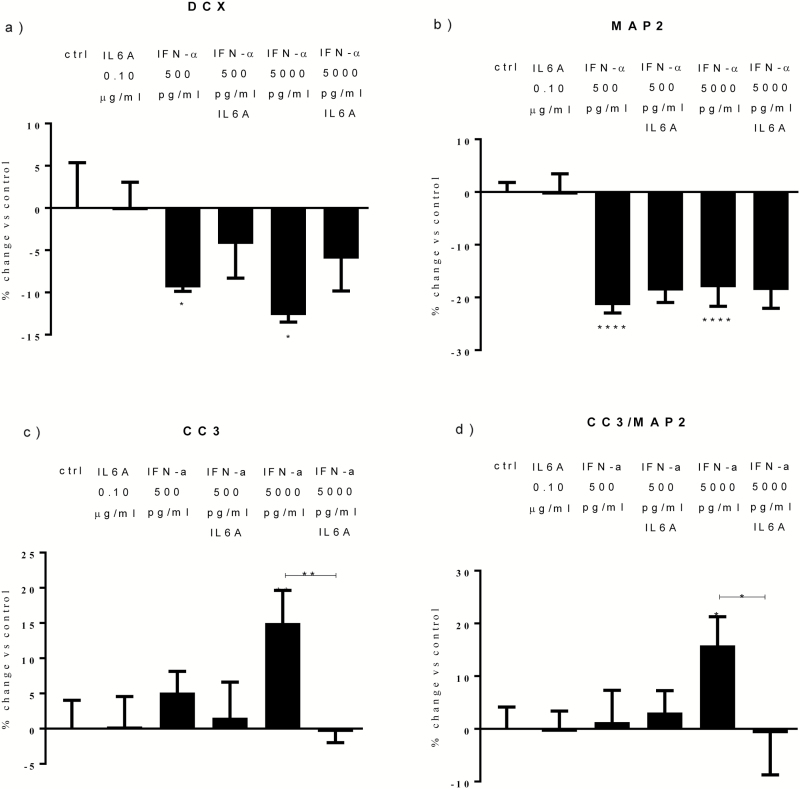
Interleukin (IL)-6A prevents the effects of interferon (IFN)-α on apoptosis. Treatment with IL-6A prevented the increase in caspase 3 (CC3)+ and CC3/microtubule-associated protein 2 (MAP2)+ cells detected upon IFN-α 5000 pg/mL (c-d). Three independent experiments were conducted on independent cultures (n=3). Data are shown as mean*±*SEM. 1-way ANOVA, Newman–Keuls’ posthoc test ^*^P<.05, ^**^P<.01, ^****^P<.0001, compared with vehicle treatment or as indicated.

With regards to apoptosis, again similarly to what we found with IFN-α, we found a significant increase in CC3+ and CC3+/MAP2+ cells following cotreatment with all proteins (+15% and +19%, Figure 2c-d). Moreover, we found a significant increase in CC3+ and CC3+/MAP2+ cells following treatment with IL-6 (+14% and +16%, Figure 2c-d), but we found a decrease in the number of CC3+ cells with USP18 (-15%, Figure 2c) and no effect with ISG15 treatment (Figure 2c). This shows that IL-6, but not ISG15 and USP18, is involved in the IFN-α-induced increase in apoptosis. This conclusion is further supported by additional mechanistic experiments where we showed that an anti-IL-6 monoclonal antibody (0.10 μg/mL) prevents the IFN-α-induced increase in apoptosis, but not the reduction in neurogenesis (Figure 3a-d).

### Treatment with a STAT1 Inhibitor Reverts the Effects of IFN-α on Neurogenesis and Apoptosis by Preventing the Upregulation of ISG15, USP18, and IL-6

**Figure 4.**
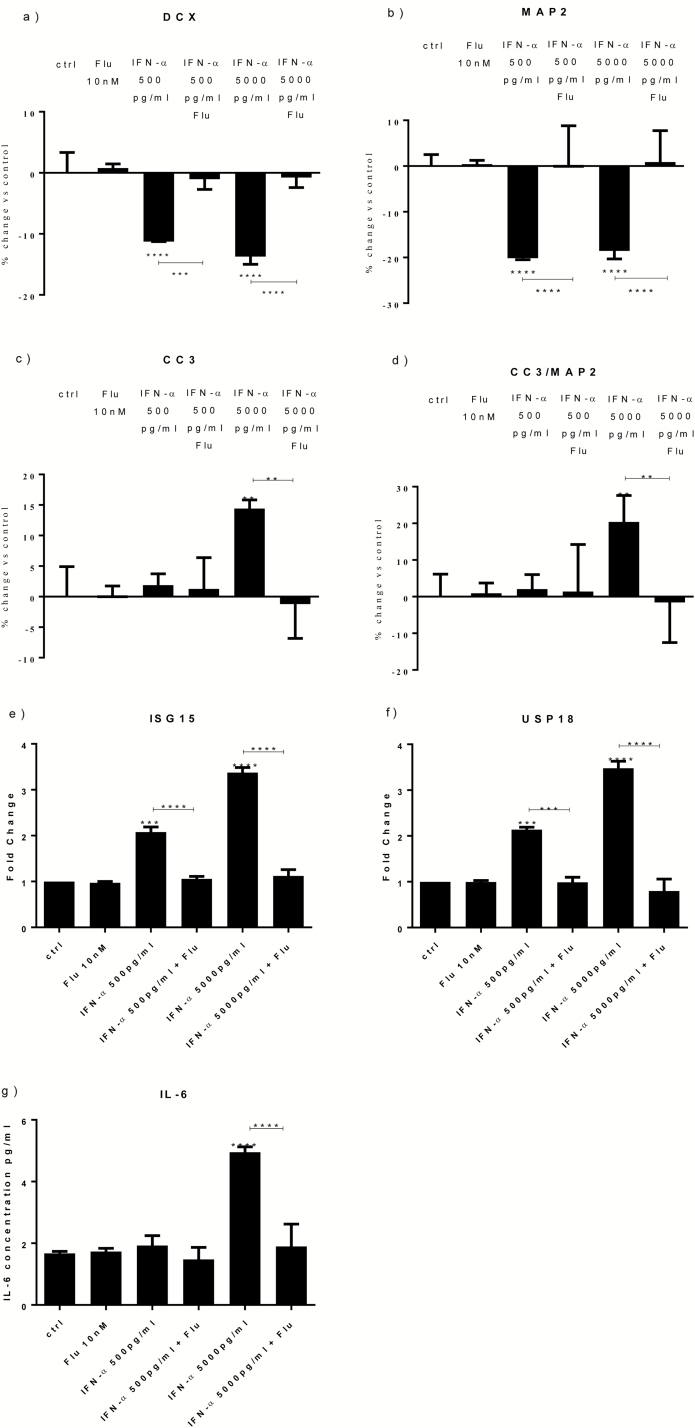
Treatment with a signal transducer and activator of transcription-1 (STAT1) inhibitor reverts the effects of interferon (IFN)-α on neurogenesis and apoptosis by preventing the upregulation of interferon-stimulated gene 15 (ISG15), ubiquitin-specific peptidase 18 (USP18), and interleukin (IL)-6. Fludarabine prevented the decrease in doublecortin (DCX)+ and microtubule-associated protein 2 (MAP2)+ cells caused by IFN-α (500 and 5000 pg/mL) (a-b), as well as the decrease in caspase 3 (CC3)+ and CC3+/MAP2+ cells detected upon IFN-α 5000 pg/mL (c-d). Fludarabine prevented the increase in ISG15 (e) and USP18 (f) genes and IL-6 protein expression (g) observed upon IFN-α (500 and 5000 pg/mL). Three independent experiments were conducted on independent cultures (n=3). Data are shown as mean*±*SEM. 1-way ANOVA, Newman–Keuls’ posthoc test ^**^P<.01, ^***^P<.001, ^****^P<.0001, compared with vehicle treatment or as indicated.

Having described such marked effects of IFN-α on neurogenesis and apoptosis, and the possible involvement of STAT1, ISG15, USP18, and IL-6 as potentially relevant mechanisms, we next investigated if and how these mechanisms were inter-connected. Therefore, cells were treated with IFN-α (500 and 5000 pg/mL) either alone or in combination with a STAT1 inhibitor, fludarabine (10 nM). We found that cotreatment with fludarabine abolished the IFN-α-induced reduction in DCX+ (Figure 4a) and MAP2+ cells (Figure 4b) at both concentrations of IFN-α. Similarly, cotreatment with fludarabine abolished the IFN-α-induced increase in CC3+ (Figure 4c) and CC3+/MAP2+ cells (Figure 4d) seen upon the high IFN-α concentration (5000 pg/mL). Finally, we also found that fludarabine blocked the upregulation of ISG15, USP18, and IL-6 (Figure 4e-g), thus indicating that STAT1 activation is required for the IFN-α-induced upregulation of these 3 molecules.

### Treatment with a STAT1 Inhibitor Prevents the Effect of ISG15 on Neurogenesis and of IL-6 on Apoptosis

**Figure 5.**
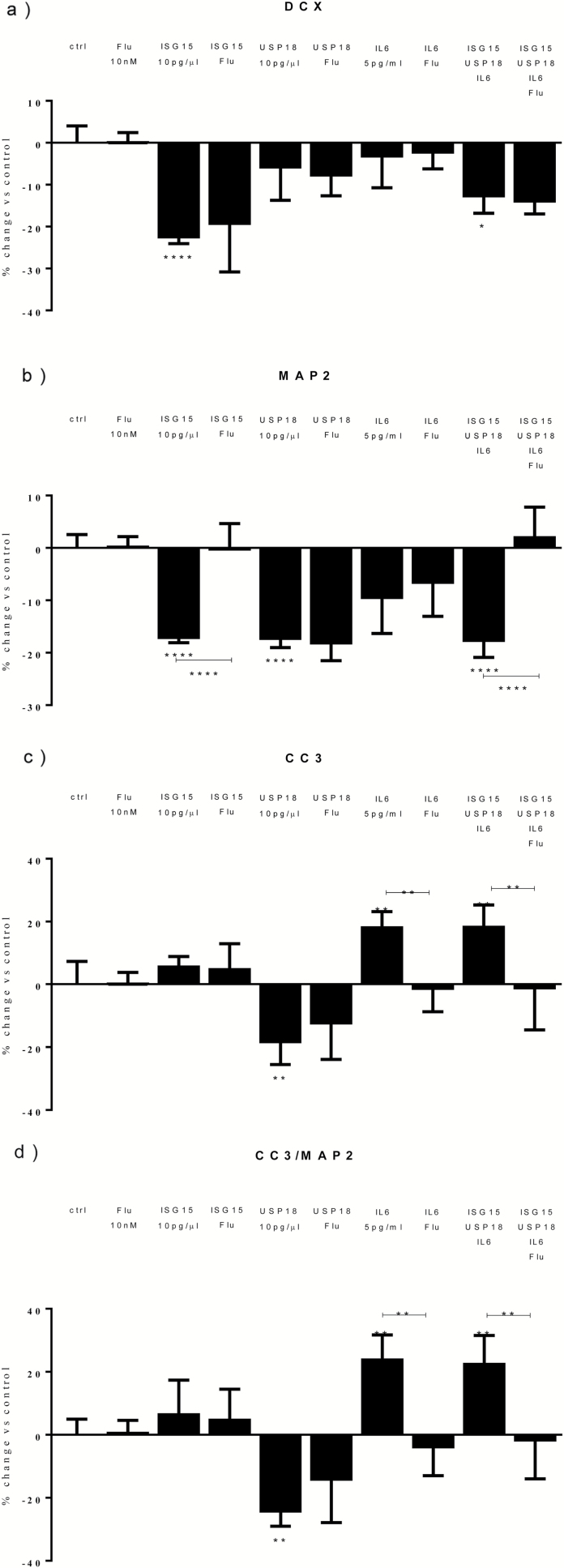
Treatment with a signal transducer and activator of transcription-1 (STAT1) inhibitor prevents the effect of interferon-stimulated gene 15 (ISG15) on neurogenesis and of interleukin (IL)-6 on apoptosis. Fludarabine prevented the decrease in microtubule-associated protein 2 (MAP2)+ cells caused by ISG15 and all proteins (b), as well as the increase in caspase 3 (CC3)+ and CC3+/MAP2+ cells detected upon treatment with IL-6 (c) and all proteins (d). Three independent experiments were conducted on independent cultures (n=3). Data are shown as mean*±*SEM. One-way ANOVA, Newman–Keuls’ posthoc test ^*^P<.05, ^**^P<.01, ^****^P<.0001, compared with vehicle treatment or as indicated.

We then wanted to investigate if STAT1 is involved also in the downstream mechanisms by which ISG15, USP18, and IL-6, respectively, reduce neurogenesis and increase apoptosis. We again treated cells with a cotreatment with ISG15, USP18, and IL-6, or with these proteins alone, and with or without fludarabine. We found that fludarabine blocked the reduction in MAP2+ cells induced by the cotreatment and by ISG15 alone (Figure 5b), as well as the increase in CC3+ and CC3+/MAP2+ cells induced by the cotreatment and by IL-6 alone (Figure 5c-d). However, fludarabine did not prevent the reduction in MAP2+ cells by USP18 (Figure 5b). This experiment suggests that the downstream effects of ISG15 and IL-6 on, respectively, neurogenesis and apoptosis are also mediated by further STAT1 activation, while the effects of USP18 on neurogenesis are mediated by a different mechanism (see supplementary Figure 4 for a summary of the findings).

### Treatment with IFN-α, ISG15, and All Proteins Increase UBA7, UBE2L6, and HERC5 Gene Expression via Activation of STAT1 Protein

**Figure 6.**
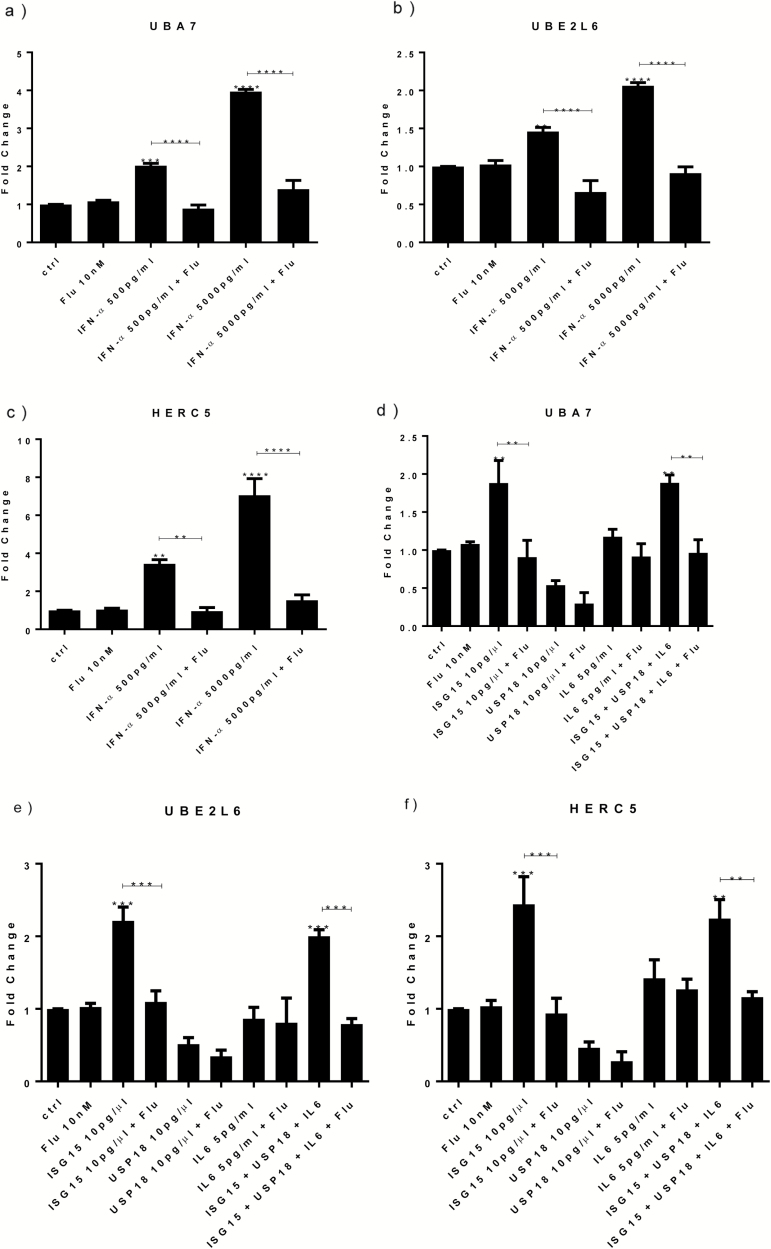
Treatment with interferon (IFN)-α, interferon-stimulated gene 15 (ISG15), and all proteins increase UBA7, UBE2L6, and HERC5 gene expression via activation of signal transducer and activator of transcription-1 (STAT1) protein. IFN-α (500 and 5000 pg/mL) increased UBA7 (a), UBE2L6 (b), and HERC5 (c) gene expression, whereas cotreatment with fludarabine prevented such upregulation (a-c). ISG15 and all proteins induced UBA7 (d), UBE2L6 (e), and HERC5 (f). Cotreatment with either ISG15 or all proteins and fludarabine prevented such increase (d-f). Three independent experiments were conducted on independent cultures (n=3). Data are shown as mean*±*SEM. One-way ANOVA, Newman–Keuls’ posthoc test ^**^P<.01, ^***^P<.001, ^****^P<.0001, compared with vehicle treatment or as indicated.

Having demonstrated that IFN-α can reduce neurogenesis via ISG15-dependent STAT1 activation, we decided to investigate which downstream mechanisms STAT1 can modulate once induced by ISG15. We focussed on 3 genes that were among the most upregulated in the transcriptomic analysis (see below) by both concentrations of IFN-α: ubiquitin like modifier activating enzyme 7 (UBA7), ubiquitin conjugating enzyme E2 L6 (UBE2L6), and HECT and RLD domain containing E3 ubiquitin protein ligase 5 (HERC5) (supplementary Table 3). These molecules, also defined as the main E1, E2, and E3 enzymes involved in ISG15 ISGylation (Zhang and Zhang, 2011), are able to target distinct cyclin proteins (Feng et al., 2008) known for their ability to regulate both cell proliferation and differentiation (Kabadi et al., 2012) and therefore represent a possible mechanism mediating IFN-α-dependent reduction of neurogenesis. Indeed, and in accordance with the microarray results, IFN-α increased UBA7, UBE2L6, and HERC5 gene expression measured by qPCR (Figure 6a-c), and this effect was blocked by cotreatment with fludarabine (Figure 6a-c).

We then wanted to test whether ISG15 was able to induce UBA7, UBE2L6, and HERC5, and whether such effect was mediated by further downstream STAT1 activation. As before, cells were treated for 3 days of proliferation followed by 7 days of differentiation, with ISG15 or cotreatment with ISG15, USP18, and IL-6, either alone or in combination with fludarabine. Results showed a significant increase in UBA7, UBE2L6, and HERC5 upon treatment with ISG15 alone or with cotreatment with all proteins (Figure 6d-f). Most interestingly, this upregulation was prevented by fludarabine (Figure 6d-f). In separate experiments, USP18 alone and IL-6 alone did not modulate these genes (Figure 6d-f). These findings suggest that STAT1-mediated upregulation of UBA7, UBE2L6, and HERC5 is a downstream mechanism involved in the ISG15-dependent pathway by which IFN-α reduces neurogenesis.

### Treatment with IFN-α or IL-6 Decreases AQP4 Gene Expression via Activation of STAT1 Protein

**Figure 7.**
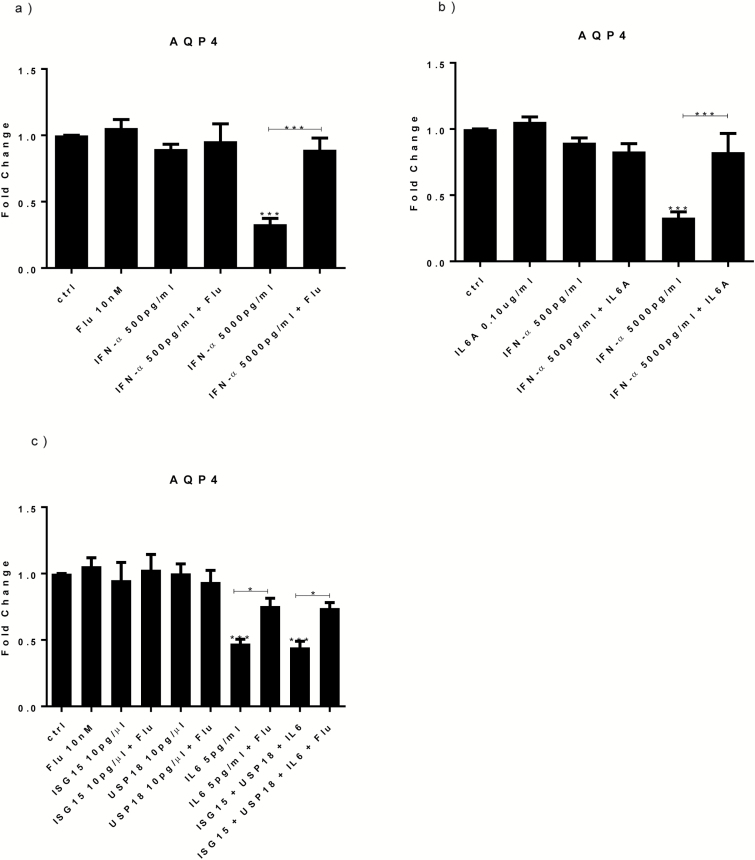
Treatment with interferon (IFN)-α, interleukin (IL)-6, and all proteins decrease AQP4 gene expression via activation of signal transducer and activator of transcription-1 (STAT1) protein. IFN-α 5000 pg/mL downregulated AQP4 gene expression, whereas cotreatment with IFN-α 5000 pg/mL and fludarabine prevented such downregulation (a). IL-6A blocked the reduction in AQP4 seen upon IFN-α 5000 pg/mL (b). Treatment with IL-6 and all proteins decreased AQP4 gene expression, whereas cotreatment of IL-6 or all proteins with fludarabine prevented the reduction in AQP4 gene expression (c). Three independent experiments were conducted on independent cultures (n=3). Data are shown as mean*±*SEM. 1-way ANOVA, Newman–Keuls’ posthoc test ^*^P<.05, ^***^P<.001, compared with vehicle treatment or as indicated.

Having demonstrated that only the high concentration of IFN-α (5000 pg/mL) alters apoptosis via IL-6-dependent STAT1 protein activation, we decided to investigate which subsequent mechanisms may be involved. We focussed on one gene that was among the most downregulated in the transcriptomic (see below) by the high concentration of IFN-α, AQP4 (Table S2). AQP4 is important for suppressing neuronal cell death, possibly by involving p53-dependent signaling (Kong et al., 2008; Bao et al., 2012). Indeed, and in accordance with the microarray results, only IFN-α 5000 pg/mL significantly decreased AQP4 gene expression measured by qPCR (Figure 7a), and this effect was blocked by cotreatment with fludarabine (Figure 7a).

We then wanted to test whether IL-6 was involved in the AQP4 gene expression downregulation induced by the high dose of IFN-α (5000 pg/mL). Indeed, results showed that the anti-IL-6 antibody blocked the reduction in AQP4 induced by IFN-α (Figure 7b). Moreover, both IL-6 alone and the cotreatment with IL-6, ISG15, and USP18 induce a significant downregulation of AQP4 (fc=-1.5 and =-1.6, respectively; Figure 7c); this effect was counteracted by fludarabine (Figure 7c). In separate experiments, ISG15 alone and USP18 alone did not modulate AQP4 (Figure 7c). These findings suggest that STAT1-mediated downregulation of AQP4 is a downstream mechanism involved in the IL-6-dependent pathway by which IFN-α increases apoptosis.

### IFN-α Modulates Signaling Pathways Involved in Cell Development, Cell Death, Oxidative Stress, and Inflammatory Response

To further identify the molecular mechanisms potentially involved in the effects of IFN-α on neurogenesis and apoptosis, we next analyzed gene expression changes by transcriptomics upon IFN-α treatment at the same time point (3 days of proliferation followed by 7 days of differentiation) as our previous immunocytochemistry experiments. Overall, IFN-α modulated 885 genes, of which 155 genes were regulated only by the lower concentration (500 pg/mL), 530 genes only by the high concentration (5000 pg/mL), and 200 genes by both concentrations (supplementary Figure 2a). The complete gene expression analysis is presented in supplementary Tables 1 to 3, whereas the network analysis is presented in supplementary Figure 3 and further discussed in supplementary Materials.

We also conducted a successful qPCR validation of 17 genes up- or downregulated in the transcriptomics analyses, chosen because of their association with neurogenesis (tryptophan hydroxylase 1, trifoil factor 3, S100 calcium binding protein A13, aldo-keto reductase family 7 member A3, and transmembrane protein 86A) (supplementary Table 4), cell death and cell cycle progression (cyclin D1, histone deacetylase 5, caspase 1, P53-Induced Death Domain Protein 1, and AQP4) (supplementary Table S4), and interferon signaling (ISG15, USP18, STAT1, IRF7, UBA7, UBE2L6, and HERC5) (supplementary Table 5), and found a very high correlation between the fold-changes detected by Affymetrix and those detected by qPCR (r=0.99; supplementary Figure 2b).

We also conducted a pathway analysis focusing our attention particularly on pathways involved in oxidative stress, neuronal formation, cell death and immune response (supplemental Table 6). Fifty-six pathways were significantly regulated only by the lower concentration of IFN-α (500 pg/mL) (which, as shown above, reduces neurogenesis but does not increase apoptosis). Of particular note are the Nuclear Factor (erythroid-derived 2)-like 2 (Nrf2)-mediated Oxidative Stress Response, endothelial Nitric Oxide Synthase signaling, Serotonin Receptor Signaling, and the Serotonin and Melatonin Biosynthesis pathways. Recent data indicate that both the Nrf2 and endothelial Nitric Oxide Synthase signaling involved in the regulation of antioxidant proteins against oxidative damage play an essential role in governing neuronal cell fate (Chen et al., 2005; Karkkainen et al., 2014). Similarly, previous evidence has shown that alteration in serotonin and melatonin signaling is also associated with impairments in cell proliferation and neuronal generation (Radley and Jacobs, 2002; Manda and Reiter, 2010; Klempin et al., 2013).

Additionally, 188 pathways were regulated only by the high concentration of IFN-α (5000 pg/mL) (which, as shown above, is the only one that increases apoptosis) (supplemental Table 6). Of particular note are the p53 signaling, Transforming Growth Factor-β signaling, Cyclin-dependent Kinase5 signaling, and FGF signaling pathways. In particular, p53 signals have been linked to cell cycle arrest and apoptosis and are known to be activated in response to DNA damage (Thotala et al., 2012). Moreover, modulation of distinct phases of the cell cycle by Transforming Growth Factor-β, Cyclin-dependent Kinase5, and FGF signals has been associated with significant alteration in neuronal differentiation (Alexander et al., 2004; He et al., 2014; Woodbury and Ikezu, 2014).

Finally, high and low concentrations of IFN-α (500 and 5000 pg/mL) modulated 165 common pathways (supplemental Table S6), among which the Cyclic Adenosine-Monophosphate Response Element Binding Protein (CREB) Signaling in Neurons and the non-canonical Wnt/Ca+ pathway, associated with cell differentiation, as well as the Activation of IRF by Cytosolic Pattern Recognition Receptors and the Protein Ubiquitination Pathway, involved in the inflammatory response. CREB signaling has a well-established role in mediating both brain development and neuronal formation (Nakagawa et al., 2002). Similarly, Wnt/Ca+ signaling is part of the mechanisms regulating neuronal motility, regeneration, and plasticity (Zheng and Poo, 2007). Finally, protein ubiquitination and IRF signaling are known not only to crucially mediate the antiviral response but also to regulate cell growth, proliferation, and differentiation (Feng et al., 2008; Baruch et al., 2014).

## Discussion

In this study, we provide the first evidence that IFN-α regulates human hippocampal neurogenesis and apoptosis, and we identify novel molecular mechanisms that underlie these effects and that are thus potentially relevant for inflammation-induced neuropsychiatric symptoms. We show in vitro that 2 concentrations of IFN-α (500 and 5000 pg/mL), similar to the lowest and the highest serum concentrations found in patients receiving IFN-α for chronic viral hepatitis, reduce human neurogenesis and the high concentration also increases human hippocampal apoptosis. We also demonstrate that both concentrations upregulate 2 IFN-α-stimulated genes, ISG15 and USP18, via STAT1-dependent mechanisms, and that either of these molecules alone can induce the same reduction in neurogenesis induced by IFN-α. In addition, only the high concentration of IFN-α enhances IL-6 protein expression, and in turn IL-6 alone can induce the same increase in apoptosis induced by IFN-α. At the molecular level, treatment with IFN-α regulates gene expression pathways involved in oxidative stress (e.g., Nrf2), immune responses (e.g., IRF signaling pathway), neuronal formation (e.g., CREB signaling), and cell death regulation (e.g., p53 signaling).

This study identifies ISG15 and USP18 as potential mediators of the IFN-α-induced reduction of human neurogenesis. Previous studies have shown that systemic injection of IFN-α reduces cell proliferation (Kaneko et al., 2006; Zheng et al., 2014) and neurogenesis (Zheng et al., 2014) in the dentate gyrus of adult rodents. Moreover, studies have also reported an increase in STAT1, ISG15, and USP18 gene expression in parenchymal neurons of mice receiving IFN-α treatment (Wang et al., 2008). We have previously confirmed the upregulation of these genes in the blood of patients treated with IFN-α for chronic viral hepatitis (Hepgul et al., 2016b), and we now show that in vitro treatment with IFN-α has the same effects in human hippocampal progenitors. In addition, we show that IFN-α upregulates ISG15 and USP18 via STAT1, and that, in turn, each of these 2 proteins alone can mimic the effects of IFN-α on neurogenesis.

Subsequently, we also show that ISG15, but not USP18 and IL-6, induces UBA7, UBE2L6, and HERC5 genes via further downstream activation of STAT1 (see supplementary Figure 4 for a summary of the findings). UBA7, UBE2L6, and HERC5 bind to ISG15 in the process of ISGylation (Zhang and Zhang, 2011), required for the biological action of ISG15, and they also independently target cyclin proteins regulating cell proliferation and differentiation (Feng et al., 2008). ISG15 ISGylation also modulates Nrf2-mediated signaling (Komatsu et al., 2010; Nakashima et al., 2015), which protects against oxidative damage, a relevant mechanism in IFN-α-induced neuronal toxicity (Alboni et al., 2013). Indeed, we also found that IFN-α downregulates Nrf2-mediated signaling pathway in the transcriptomics analysis. Taken together, these findings identify STAT1-mediated upregulation of UBA7, UBE2L6, and HERC5 as a potential mechanism in the ISG15-dependent reduction in neurogenesis by IFN-α.

Of note, we did not find evidence that STAT1 mediates the USP18-dependent reduction in neurogenesis. However, the transcriptomics analysis shows that the Notch signaling pathway is regulated by both the low and the high concentrations of IFN-α, albeit trough different molecules (see supplemental Table 6). This pathway is well known for its ability to govern proliferation and neurogenesis (Xiao et al., 2009), and USP18, via autophagic mechanisms, can induce Notch degradation (Xu et al., 2015). Therefore, we can speculate that the USP18-induced reduction in neurogenesis involves the Notch signaling pathways.

We also show that IFN-α is responsible for cell apoptosis via STAT1-mediated production of IL-6, measured as protein in the supernatant of our cells. Of note, the transcriptomics analysis does not identify IL-6 as one of the genes regulated by IFN-α (see supplemental Tables 1–3). This is not surprising, as only around 30% to 40% of the variance in protein abundance is explained by mRNA abundance (Vogel and Marcotte, 2012). Interestingly, an important ISG, the IRF7 gene, is significantly upregulated by IFN-α (see supplemental Table 3). IFN-α-induced IRF7 has been previously shown to increase IL-6 protein secretion without influencing the transcripts (Sweeney, 2011), thus offering an explanation for our findings. Interestingly, the highest concentration of IFN-α induces up to 5 pg/mL of IL-6, a concentration similar to that found in both plasma and CSF of HCV patients who developed IFN-α-induced depression (Wichers et al., 2006; Raison et al., 2009), and this is the concentration of IL-6 that we use in our in vitro experiments. In contrast, in a previous study investigating the effect of IL-6 on neurogenesis using our same cellular model, Johansson et al. (2008) used a much higher concentration of the IL-6 (50000 pg/mL) and found an opposite effect on neurogenesis (Johansson et al., 2008), but their concentration is unlikely to be physiologically relevant. Of note, in contrast with IFN-α and IL-6, USP18 decreases apoptosis; this is consistent with previous evidence in brain ependymal cells (Ritchie et al., 2002). Clearly, IL-6 must be able to overcome these putative protective effects of USP18.

Finally, upon the highest concentration of IFN-α and with IL-6 alone, we also show a downregulation of the AQP4 gene, again via STAT1 activation. AQP4 is important for suppressing neuronal cell death (Kong et al., 2008), possibly by involving p53-dependent signaling (Bao et al., 2012), and, in fact, p53-dependent signaling pathway is regulated by the high concentration of IFN-α in our transcriptomics analysis. Taken together, these data indicate that IL-6-induced inhibition of AQP4 (possibly leading to changes in p53 signaling) is a potential mechanism through which IFN-α induces neuronal apoptosis.

Overall, our findings help clarify the molecular mechanisms underpinning the detrimental effects of IFN-α on both neurogenesis and apoptosis, and these mechanisms have translational potential. It is well known that therapeutic use of IFN-α is associated with many neuropsychiatric side effects, including depression, anxiety, and cognitive abnormalities (Raison et al., 2005; Hepgul et al., 2016a). Interestingly, the aforementioned animal studies have shown a significant upregulation of STAT1, ISG15, and USP18 genes in the mouse brain, including the hippocampus (Wang and Campbell, 2005; Wang et al., 2008), a brain region involved in neurogenesis-dependent cognitive functions, such as memory and attention, often impaired in patients with depression (Moylan et al., 2013). Indeed, postmortem studies have confirmed that patients with major depression have reduced hippocampal neurogenesis (Boldrini et al., 2013) and that in vitro treatment with antidepressants can revert or prevent this effect (Hui et al., 2014). Moreover, one study has shown a trend towards a greater increase in ISG15 gene expression in peripheral leukocytes of patients with IFN-α-induced depression (Schlaak et al., 2012). Therefore, our study and others suggest that IFN-α therapy can activate IFN-stimulated molecules in the blood as well as in specific regions of the brain, leading to neurogenic alterations that are associated with cognitive and behavioral disturbances. We would further argue that those molecules might be regarded as valuable therapeutic targets for future treatments of neuropsychiatric disorders. IL-6 antagonists developed for autoimmune disorders are currently tested in clinical trials for depression (see, for example, ClinicalTrials.gov identifier NCT02473289), and ISG15 inhibition has been proposed as an antiviral strategy and could be repurposed for psychiatric indications (Hermann and Bogunovic, 2017).

A possible limitation of our in vitro model is that the immortalized cell line, while being of invaluable importance for our understanding of molecular mechanisms occurring in the hippocampus, may differ from the scenario of an adult organism in vivo, especially because of the absence of microglia cells. However, all our previous results with this in vitro model have been replicated by animal or clinical studies, including changes in neurogenesis by cortisol, IL-1, and antidepressants, and changes in stress- and antidepressants-regulated genes (Anacker et al., 2011; Zunszain et al., 2012; Anacker et al., 2013a, 2013b; Horowitz et al., 2014; Borsini et al., 2017). Therefore, we are confident that our results are relevant to the human brain. Of note, we did not assess the effect of IFN-α on astrogliogenesis or oligodendrogenesis, but previous in vitro evidence has reported no changes in gliogenesis upon IFN-α treatment (Zheng et al., 2014).

In summary, our study reveals the ability of IFN-α to reduce human neurogenesis and to enhance human apoptosis in human hippocampal precursor cells. We also demonstrate a critical role of STAT1-dependent mechanisms that involve ISG15, USP18, and IL-6 for these effects of IFN-α. The presence of a cell population in the hippocampus that is highly sensitive to IFN-α, responding with a dramatic activation of IFN-induced genes, highlights the potential roles of the hippocampus in the IFN-α-induced psychopathology and potentially in the mechanisms leading to inflammation-induced depression.

## Funding

This work was supported by the grants Immunopsychiatry: a Consortium to Test the Opportunity for Immunotherapeutics in Psychiatry (MR/L014815/1) and Persistent Fatigue Induced by Interferon-alpha: A New Immunological Model for Chronic Fatigue Syndrome (MR/J002739/1) from the Medical Research Council (UK). Additional support has been offered by the National Institute for Health Research Mental Health Biomedical Research Centre in Mental Health at South London and Maudsley NHS Foundation Trust and King’s College London. The views expressed are those of the authors and not necessarily those of the NHS, the NIHR, or the Department of Health. Dr Cattaneo is also funded by the Eranet Neuron Inflame-D. Dr Thuret is also funded by the Welton Foundation, the Alzheimer’s Society, and the MRC. Dr Zunszain is also funded by the MRC.

## Statement of Interest

The authors have received research funding from Janssen Pharmaceutical NV/Janssen Pharmaceutical Companies of Jonhson&Jonhson (A.B., P.A.Z., S.T., C.M.P.). C.M.P. has also received speaker’s fees from Lundbeck and consultation fees from Consultant to Eleusis Benefit Corporation.

## Supplementary Material

Supplementary Table 1

Supplementary Table 2

Supplementary Table 3

Supplementary Table 4

Supplementary Table 5

Supplementary Table 6

Supplementary Figure 1

Supplementary Figure 2

Supplementary Figure 3

Supplementary Figure 4

Supplementary_Materials
