# Supplementary material for: Interferon-Alpha Reduces Human Hippocampal Neurogenesis and Increases Apoptosis via Activation of Distinct STAT1-Dependent Mechanisms
Source: Int J Neuropsychopharmacol. 2017 Oct 10;21(2):187–200. doi: 10.1093/ijnp/pyx083 (PMC5793815; doi:10.1093/ijnp/pyx083)
Supplement: Supplementary Table 1 [file pyx083_suppl_supplementary_table_1.docx]

**Supplementary Table 1. Genes Regulated by IFN-α 500 pg/mL Only When Compared with Vehicle**

| **Elements in IFN-α 500 pg/ml only vs vehicle** | | |  |  |
| --- | --- | --- | --- | --- |
|  |  |  |  |  |
|  | **Gene** | **Gene Title** | ***P*** | **FC** |
| **1** | AKR7A3 | aldo-keto reductase family 7, member A3 | .0211 | -1.7509 |
| **2** | GBP4 | guanylate binding protein 4 | .0204 | -1.6057 |
| **3** | PDE10A | phosphodiesterase 10A | .0014 | -1.4772 |
| **4** | MSTO1 | misato 1, mitochondrial distribution and morphology regulator | .0499 | -1.4522 |
| **5** | IGKV3-15 | immunoglobulin kappa variable 3-15 | .0377 | -1.4365 |
| **6** | RAP2CP1 | RAP2CP1 // RAP2C pseudogene 1 | .0330 | -1.4317 |
| **7** | ITIH5 | inter-alpha-trypsin inhibitor heavy chain family, member 5 | .0186 | -1.4148 |
| **8** | PGM5-AS1 | PGM5 antisense RNA 1 | .0149 | -1.3783 |
| **9** | ESRG | embryonic stem cell related (non-protein coding) | .0463 | -1.3581 |
| **10** | OR7E2P | olfactory receptor, family 7, subfamily E, member 2 pseudogene | .0016 | -1.3354 |
| **11** | GEMIN8P4 | gem (nuclear organelle) associated protein 8 pseudogene 4 | .0436 | -1.3319 |
| **12** | WDR7 | WD repeat domain 7 | .0301 | -1.3197 |
| **13** | SCARNA3 | small Cajal body-specific RNA 3 | .0351 | -1.3138 |
| **14** | RRP7B | ribosomal RNA processing 7 homolog B (S. cerevisiae) | .0211 | -1.3112 |
| **15** | OR52E8 | olfactory receptor, family 52, subfamily E, member 8 | .0217 | -1.3069 |
| **16** | OR4K13 | olfactory receptor, family 4, subfamily K, member 13 | .0425 | -1.2980 |
| **17** | IGFL3 | IGF-like family member 3 | .0086 | -1.2967 |
| **18** | LPAR1 | lysophosphatidic acid receptor 1 | .0014 | -1.2951 |
| **19** | ZNF300 | zinc finger protein 300 | .0186 | -1.2930 |
| **20** | USP12-AS2 | USP12 antisense RNA 2 (head to head) | .0128 | -1.2911 |
| **21** | DAZL | deleted in azoospermia-like | .0395 | -1.2893 |
| **22** | TIGD5 | tigger transposable element derived 5 | .0065 | -1.2891 |
| **23** | GLYATL2 | glycine-N-acyltransferase-like 2 | .0053 | -1.2870 |
| **24** | ANKRD1 | ankyrin repeat domain 1 (cardiac muscle) | .0222 | -1.2858 |
| **25** | CSDC2 | cold shock domain containing C2, RNA binding | .0267 | -1.2857 |
| **26** | ZSCAN22 | zinc finger and SCAN domain containing 22 | .0021 | -1.2840 |
| **27** | KCTD21-AS1 | KCTD21 antisense RNA 1 | .0294 | -1.2798 |
| **28** | AP000470.2 | putative novel transcript | .0206 | -1.2791 |
| **29** | RPL26L1 | ribosomal protein L26-like 1 | .0048 | -1.2788 |
| **30** | ZBED9 | zinc finger, BED-type containing 9 | .0182 | -1.2696 |
| **31** | GCSAML-AS1 | GCSAML antisense RNA 1 | .0385 | -1.2681 |
| **32** | NCR1 | natural cytotoxicity triggering receptor 1 | .0188 | -1.2634 |
| **33** | ANXA3 | annexin A3 | .0133 | -1.2623 |
| **34** | TFF3 | trefoil factor 3 (intestinal) | .0229 | -1.2562 |
| **35** | IPW | imprinted in Prader-Willi syndrome (non-protein coding) | .0265 | -1.2557 |
| **36** | C4orf6 | chromosome 4 open reading frame 6 | .0229 | -1.2549 |
| **37** | PSORS1C2 | psoriasis susceptibility 1 candidate 2 | .0428 | -1.2538 |
| **38** | CYB561A3 | cytochrome b561 family, member A3 | .0138 | -1.2534 |
| **39** | DND1 | DND microRNA-mediated repression inhibitor 1 | .0388 | -1.2533 |
| **40** | OR4S1 | olfactory receptor, family 4, subfamily S, member 1 | .0148 | -1.2518 |
| **41** | CNN1 | calponin 1, basic, smooth muscle | .0363 | -1.2485 |
| **42** | TPH1 | tryptophan hydroxylase 1 | .0081 | -1.2408 |
| **43** | FAM9C | family with sequence similarity 9, member C | .0391 | -1.2406 |
| **44** | RUNX1-IT1 | RUNX1 intronic transcript 1 (non-protein coding) | .0412 | -1.2395 |
| **45** | KIAA1656 | KIAA1656 protein | .0026 | -1.2386 |
| **46** | JAKMIP2-AS1 | JAKMIP2 antisense RNA 1 | .0094 | -1.2377 |
| **47** | CCDC103 | coiled-coil domain containing 103 | .0162 | -1.2349 |
| **48** | OR4C45 | olfactory receptor, family 4, subfamily C, member 45 | .0127 | -1.2344 |
| **49** | CYP4F11 | cytochrome P450, family 4, subfamily F, polypeptide 11 | .0141 | -1.2340 |
| **50** | IGLV1-50 | immunoglobulin lambda variable 1-50 (non-functional) | 0324 | -1.2336 |
| **51** | PCDH9-AS1 | PCDH9 antisense RNA 1 | .0086 | -1.2317 |
| **52** | HOXD9 | homeobox D9 | .0353 | -1.2305 |
| **53** | S100A13 | S100 calcium binding protein | .0368 | -1.2278 |
| **54** | TMEM86A | transmembrane protein 86A | .0481 | -1.2271 |
| **55** | CA13 | carbonic anhydrase XIII | .0001 | -1.2265 |
| **56** | FIBCD1 | fibrinogen C domain containing 1 | .0212 | -1.2259 |
| **57** | CHMP1B2P | charged multivesicular body protein 1B2, pseudogene | .0161 | -1.2228 |
| **58** | FAM69A | family with sequence similarity 69, member A | .0229 | -1.2221 |
| **59** | TES | testis derived transcript (3 LIM domains) | 0019 | -1.2207 |
| **60** | SELL | selectin L | .0185 | -1.2200 |
| **61** | GRHL3 | grainyhead-like 3 (Drosophila) | .0143 | -1.2168 |
| **62** | MFSD3 | major facilitator superfamily domain containing 3 | .0347 | -1.2158 |
| **63** | UIMC1 | ubiquitin interaction motif containing 1 | .0030 | -1.2153 |
| **64** | AGAP2 | ArfGAP with GTPase domain, ankyrin repeat and PH domain 2 | .0011 | -1.2145 |
| **65** | FAM46B | family with sequence similarity 46, member B | .0164 | -1.2143 |
| **66** | NT5C1A | 5-nucleotidase, cytosolic IA | .0212 | -1.2124 |
| **67** | MMP14 | matrix metallopeptidase 14 (membrane-inserted) | .0076 | -1.2115 |
| **68** | CHCHD1 | coiled-coil-helix-coiled-coil-helix domain containing 1 | .0022 | -1.2109 |
| **69** | KLHL34 | kelch-like family member 34 | .0064 | -1.2106 |
| **70** | MUC1 | mucin 1, cell surface associated | .0079 | -1.2079 |
| **71** | NPL | N-acetylneuraminate pyruvate lyase | .0269 | -1.2073 |
| **72** | OR4N5 | olfactory receptor, family 4, subfamily N, member 5 | .0315 | -1.2070 |
| **73** | DDX11L2 | DEAD/H (Asp-Glu-Ala-Asp/His) box helicase 11 like 2 | .0052 | -1.2064 |
| **74** | OR52E6 | olfactory receptor, family 52, subfamily E, member 6 | .0119 | -1.2057 |
| **75** | CMC2 | C-x(9)-C motif containing 2 | .0012 | -1.2049 |
| **76** | WDR20 | WD repeat domain 20 | .0067 | -1.2022 |
| **77** | FAM198B | family with sequence similarity 198, member B | .0161 | -1.2014 |
| **78** | DOCK11 | dedicator of cytokinesis 11 | .0199 | -1.2002 |
| **79** | HEY2 | hes-related family bHLH transcription factor with YRPW motif 2 | .0340 | 1.2004 |
| **80** | SLC2A9 | solute carrier family 2 (facilitated glucose transporter), member 9 | .0241 | 1.2006 |
| **81** | TEX15 | testis expressed 15 | .0327 | 1.2030 |
| **82** | KRTAP10-11 | keratin associated protein 10-11 | .0402 | 1.2032 |
| **83** | ADARB1 | adenosine deaminase, RNA-specific, B1 | .0273 | 1.2043 |
| **84** | ADAMTS3 | ADAM metallopeptidase with thrombospondin type 1 motif, 3 | .0421 | 1.2045 |
| **85** | SCGB1D2 | secretoglobin, family 1D, member 2 | .0261 | 1.2046 |
| **86** | EVA1B | eva-1 homolog B (C. elegans) | .0174 | 1.2053 |
| **87** | FCRL5 | Fc receptor-like 5 | .0449 | 1.2062 |
| **88** | MTX3 | metaxin 3 | .0414 | 1.2092 |
| **89** | CCL25 | chemokine (C-C motif) ligand 25 | .0266 | 1.2105 |
| **90** | MSANTD1 | Myb/SANT-like DNA-binding domain containing 1 | .0085 | 1.2121 |
| **91** | DPEP2 | dipeptidase 2 | .0066 | 1.2123 |
| **92** | MTBP | MDM2 binding protein | .0034 | 1.2125 |
| **93** | EPSTI1 | epithelial stromal interaction 1 (breast) | .0396 | 1.2143 |
| **94** | FAM185A | family with sequence similarity 185, member A | .0286 | 1.2155 |
| **95** | CXCR3 | chemokine (C-X-C motif) receptor 3 | .0129 | 1.2157 |
| **96** | SCGB2A1 | secretoglobin, family 2A, member 1 | .0092 | 1.2157 |
| **97** | MCIDAS | multiciliate differentiation and DNA synthesis associated protein | .0161 | 1.2159 |
| **98** | STAG3L5P | STAG3L5P-PVRIG2P-PILRB readthrough | .0369 | 1.2162 |
| **99** | PLEKHG4 | pleckstrin homology domain containing, family G4 | .0488 | 1.2172 |
| **100** | TRBV28 | T cell receptor beta variable 28 | .0275 | 1.2181 |
| **101** | POTEG | POTE ankyrin domain family, member G | .0479 | 1.2183 |
| **102** | XXbac-BPG13B8 | XXbac-BPG13B8.10 | .0350 | 1.2190 |
| **103** | GNRHR2 | gonadotropin-releasing hormone (type 2) receptor 2 | .0429 | 1.2193 |
| **104** | NEK9 | NIMA-related kinase 9 | .0011 | 1.2211 |
| **105** | XKR5 | XK, Kell blood group complex subunit-related family, member 5 | .0460 | 1.2222 |
| **106** | TMEM17 | transmembrane protein 17 | .0295 | 1.2223 |
| **107** | FAM197Y2 | family with sequence similarity 197, Y-linked, member 2 | .0210 | 1.2224 |
| **108** | RPL23 | ribosomal protein L23 | .0376 | 1.2228 |
| **109** | ATP1A3 | ATPase, Na+/K+ transporting, alpha 3 polypeptide | .0113 | 1.2232 |
| **110** | BATF | basic leucine zipper transcription factor, ATF-like | .0361 | 1.2234 |
| **111** | ALDH5A1 | aldehyde dehydrogenase 5 family, member A1 | .0068 | 1.2240 |
| **112** | RNF113A | ring finger protein 113A | .0383 | 1.2277 |
| **113** | TBC1D3 | TBC1 domain family, member 3 | .0457 | 1.2283 |
| **114** | ZNF804B | zinc finger protein 804B | .0210 | 1.2294 |
| **115** | TISP43 | uncharacterized LOC150527 | .0113 | 1.2306 |
| **116** | HOXC-AS2 | HOXC cluster antisense RNA 2 | .0148 | 1.2307 |
| **117** | SLC22A18 | solute carrier family 22, member 18 | .0359 | 1.2308 |
| **118** | LMX1B | LIM homeobox transcription factor 1, beta | .0196 | 1.2309 |
| **119** | ECRP | ribonuclease, RNase A family, 2 | .0243 | 1.2312 |
| **120** | KCNA1 | potassium voltage-gated channel, shaker-related subfamily | .0164 | 1.2313 |
| **121** | CHRM4 | cholinergic receptor, muscarinic 4 | .0441 | 1.2376 |
| **122** | CASP7 | caspase 7, apoptosis-related cysteine peptidase | .0249 | 1.2380 |
| **123** | SRGAP3-AS3 | SRGAP3 antisense RNA 3 | .0158 | 1.2395 |
| **124** | MAL2 | mal, T-cell differentiation protein 2 (gene/pseudogene) | .0024 | 1.2405 |
| **125** | PRSS3 | protease, serine, 3 | .0012 | 1.2405 |
| **126** | C17orf82 | chromosome 17 open reading frame 82 | .0114 | 1.2407 |
| **127** | F7 | coagulation factor VII (serum prothrombin conversion accelerator) | .0123 | 1.2474 |
| **128** | BX470102.3 | Novel transcript | .0275 | 1.2510 |
| **129** | MCTP2 | multiple C2 domains, transmembrane 2 | .0328 | 1.2513 |
| **130** | STARD9 | StAR-related lipid transfer (START) domain containing 9 | .0359 | 1.2563 |
| **131** | BSN-AS1 | BSN antisense RNA 1 | .0337 | 1.2583 |
| **132** | IGKV3-7 | immunoglobulin kappa variable 3-7 (non-functional) | .0343 | 1.2613 |
| **133** | ISPD-AS1 | ISPD antisense RNA 1 | .0306 | 1.2674 |
| **134** | ZNF714 | zinc finger protein 714 | .0169 | 1.2679 |
| **135** | TRIM77 | tripartite motif containing 77 | .0138 | 1.2711 |
| **136** | EIF4B | eukaryotic translation initiation factor 4B | .0417 | 1.2839 |
| **137** | NBPF26 | neuroblastoma breakpoint family, member 26 | .0103 | 1.2853 |
| **138** | PTK6 | protein tyrosine kinase 6 | .0377 | 1.2876 |
| **139** | AACSP1 | acetoacetyl-CoA synthetase pseudogene 1 | .0385 | 1.2900 |
| **140** | ALPK3 | alpha-kinase 3 | .0468 | 1.2923 |
| **141** | FAM138D | family with sequence similarity 138, member D | .0118 | 1.2933 |
| **142** | USP32P2 | ubiquitin specific peptidase 32 pseudogene 2 | .0339 | 1.2980 |
| **143** | CCNT2-AS1 | CCNT2 antisense RNA 1 | .0207 | 1.3135 |
| **144** | MND1 | meiotic nuclear divisions 1 homolog (S. cerevisiae) | .0040 | 1.3244 |
| **145** | HNRNPA1P33 | heterogeneous nuclear ribonucleoprotein A1 pseudogene 33 | .0350 | 1.3268 |
| **146** | GOLGA8R | golgin A8 family, member R | .0417 | 1.3269 |
| **147** | GPR42 | G protein-coupled receptor 42 (gene/pseudogene) | .0239 | 1.3404 |
| **148** | PCMTD2 | protein-L-isoaspartate (D-aspartate) O-methyltransferase domain 2 | .0362 | 1.3497 |
| **149** | OR7E47P | olfactory receptor, family 7, subfamily E, member 47 pseudogene | .0258 | 1.3535 |
| **150** | FLJ41733 | FLJ41733 protein | .0386 | 1.3665 |
| **151** | PPIE | peptidylprolyl isomerase E (cyclophilin E) | .0015 | 1.3841 |
| **152** | BHLHA15 | basic helix-loop-helix family, member a15 | .0046 | 1.4033 |
| **153** | FAM72C | family with sequence similarity 72, member C | .0244 | 1.4136 |
| **154** | MSRA | methionine sulfoxide reductase A | .0163 | 1.4443 |
| **155** | IGKV1-39 | immunoglobulin kappa variable 1-39 (gene/pseudogene) | .0161 | 1.6674 |
