# Supplementary material for: Interferon-Alpha Reduces Human Hippocampal Neurogenesis and Increases Apoptosis via Activation of Distinct STAT1-Dependent Mechanisms
Source: Int J Neuropsychopharmacol. 2017 Oct 10;21(2):187–200. doi: 10.1093/ijnp/pyx083 (PMC5793815; doi:10.1093/ijnp/pyx083)
Supplement: Supplementary Table 2 [file pyx083_suppl_supplementary_table_2.docx]

**Supplementary Table 2. Genes Regulated by IFN-α 5000 pg/mL Only When Compared with Vehicle**

| **Elements in IFN-α 5000 pg/mL only vs vehicle** | | |  |  |
| --- | --- | --- | --- | --- |
|  |  |  |  |  |
|  | **Gene Symbol** | **Gene Title** | ***P*** | **FC** |
| **1** | SCGN | secretagogin, EF-hand calcium binding protein | .0024 | -2.1694 |
| **2** | C1QL4 | complement component 1, q subcomponent-like 4 | .0002 | -2.0742 |
| **3** | CALB2 | calbindin 2 | .0007 | -2.0366 |
| **4** | DLX6-AS1 | DLX6 antisense RNA 1 | .0015 | -1.7995 |
| **5** | DCX | doublecortin | .0004 | -1.7895 |
| **6** | DLX5 | distal-less homeobox 5 | .0017 | -1.6871 |
| **7** | ATCAY | ataxia, cerebellar, Cayman type | .0003 | -1.6814 |
| **8** | ST8SIA5 | ST8 alpha-N-acetyl-neuraminide alpha-2,8-sialyltransferase 5 | .0003 | -1.6624 |
| **9** | IGLV3-19 | immunoglobulin lambda variable 3-19 | .0413 | -1.6343 |
| **10** | GAD2 | decarboxylase 2 (pancreatic islets and brain, 65kDa) | .0090 | -1.6152 |
| **11** | MMRN1 | multimerin 1 | .0001 | -1.5904 |
| **12** | FREM2 | FRAS1 related extracellular matrix protein 2 | .0024 | -1.5678 |
| **13** | SPARCL1 | SPARC-like 1 (hevin) | .0023 | -1.5550 |
| **14** | DLX6 | distal-less homeobox 6 | .0005 | -1.5442 |
| **15** | ABCC9 | ATP-binding cassette, sub-family C (CFTR/MRP), member 9 | .0004 | -1.5163 |
| **16** | TMSB15A | thymosin beta 15a | .0149 | -1.5152 |
| **17** | PLXNA4 | plexin A4 | .0029 | -1.5102 |
| **18** | NPY | neuropeptide Y | .0009 | -1.5031 |
| **19** | PWAR5 | Prader Willi/Angelman region RNA 5 | .0021 | -1.4969 |
| **20** | PCDHB18 | protocadherin beta 18 pseudogene | .0156 | -1.4837 |
| **21** | GPR158 | G protein-coupled receptor 158 | .0010 | -1.4830 |
| **22** | TAS2R46 | taste receptor, type 2, member 46 | .0252 | -1.4817 |
| **23** | ZNF836 | zinc finger protein 836 | .0105 | -1.4741 |
| **24** | AQP4 | aquaporin 4 | .0001 | -1.4701 |
| **25** | ELAVL3 | ELAV like neuron-specific RNA binding protein 3 | .0120 | -1.4666 |
| **26** | NYNRIN | NYN domain and retroviral integrase containing | .0014 | -1.4591 |
| **27** | GDPD2 | glycerophosphodiester phosphodiesterase domain containing 2 | .0005 | -1.4516 |
| **28** | MOB3B | MOB kinase activator 3B | .0116 | -1.4442 |
| **29** | CCDC30 | coiled-coil domain containing 30 | .0096 | -1.4378 |
| **30** | SLC6A11 | solute carrier family 6 (neurotransmitter transporter), member 11 | .0001 | -1.4353 |
| **31** | LRP1B | low density lipoprotein receptor-related protein 1B | .0056 | -1.4329 |
| **32** | LPPR3 | lipid phosphate phosphatase-related protein type 3 | .0105 | -1.4281 |
| **33** | FRZB | frizzled-related protein | .0018 | -1.4279 |
| **34** | MMP16 | matrix metallopeptidase 16 (membrane-inserted) | .0018 | -1.4265 |
| **35** | LPPR4 | lipid phosphate phosphatase-related protein type 4 | .0010 | -1.4245 |
| **36** | CRYM | crystallin, mu | .0160 | -1.4232 |
| **37** | C10orf107 | chromosome 10 open reading frame 107 | .0059 | -1.4224 |
| **38** | RLBP1 | retinaldehyde binding protein 1 | .0246 | -1.4200 |
| **39** | SNURF | SNRPN upstream reading frame | .0153 | -1.4188 |
| **40** | PTCHD1 | patched domain containing 1 | .0071 | -1.4176 |
| **41** | C12orf55 | chromosome 12 open reading frame 55 | .0204 | -1.4154 |
| **42** | DKFZP434L187 | uncharacterized LOC26082 | .0020 | -1.4105 |
| **43** | RASD1 | RAS, dexamethasone-induced 1 | .0142 | -1.4102 |
| **44** | LRRTM2 | leucine rich repeat transmembrane neuronal 2 | .0003 | -1.4095 |
| **45** | SPANXC | SPANX family, member C | .0266 | -1.3959 |
| **46** | OLIG1 | oligodendrocyte transcription factor 1 | .0222 | -1.3923 |
| **47** | LILRA4 | eukocyte immunoglobulin-like receptor, subfamily A 4 | .0334 | -1.3875 |
| **48** | S1PR1 | sphingosine-1-phosphate receptor 1 | .0018 | -1.3845 |
| **49** | ROBO2 | roundabout, axon guidance receptor, homolog 2 (Drosophila) | .0261 | -1.3819 |
| **50** | CLVS2 | clavesin 2 | .0068 | -1.3759 |
| **51** | ATRNL1 | attractin-like 1 | .0013 | -1.3758 |
| **52** | SLC25A2 | solute carrier family 25 (mitochondrial carrier), member 2 | .0008 | -1.3722 |
| **53** | MTURN | maturin, neural progenitor differentiation regulator homolog | .0001 | -1.3713 |
| **54** | CHST6 | carbohydrate (N-acetylglucosamine 6-O) sulfotransferase 6 | .0033 | -1.3702 |
| **55** | TAC3 | tachykinin 3 | .0299 | -1.3683 |
| **56** | EDNRB | endothelin receptor type B | .0007 | -1.3677 |
| **57** | S100B | S100 calcium binding protein B | .0021 | -1.3650 |
| **58** | BCHE | butyrylcholinesterase | .0014 | -1.3623 |
| **59** | MOXD1 | monooxygenase, DBH-like 1 | .0005 | -1.3617 |
| **60** | C11orf87 | chromosome 11 open reading frame 87 | .0018 | -1.3613 |
| **61** | MAOB | monoamine oxidase B | .0004 | -1.3566 |
| **62** | PPEF1 | protein phosphatase, EF-hand calcium binding domain 1 | .0278 | -1.3547 |
| **63** | SEL1L3 | sel-1 suppressor of lin-12-like 3 (C. elegans) | .0003 | -1.3542 |
| **64** | ATP1A2 | ATPase, Na+/K+ transporting, alpha 2 polypeptide | .0039 | -1.3480 |
| **65** | KANK4 | KN motif and ankyrin repeat domains 4 | .0124 | -1.3466 |
| **66** | FAM197Y9 | family with sequence similarity 197, Y-linked, member 9 | .0102 | -1.3458 |
| **67** | GATSL2 | GATS protein-like 2 | .0015 | -1.3448 |
| **68** | TCEB3CL2 | transcription elongation factor B polypeptide 3C-like 2 | .0179 | -1.3447 |
| **69** | ETV1 | ets variant 1 | .0022 | -1.3443 |
| **70** | ELAVL4 | ELAV like neuron-specific RNA binding protein 4 | .0100 | -1.3398 |
| **71** | BGN | biglycan | .0170 | -1.3372 |
| **72** | HUNK | hormonally up-regulated Neu-associated kinase | .0158 | -1.3363 |
| **73** | ANKRD30BP3 | ankyrin repeat domain 30B pseudogene 3 | .0247 | -1.3301 |
| **74** | MPST | mercaptopyruvate sulfurtransferase | .0225 | -1.3293 |
| **75** | SLC4A4 | solute carrier family 4, member 4 | 0476 | -1.3292 |
| **76** | EEF1A1P22 | eukaryotic translation elongation factor 1 alpha 1 pseudogene 22 | .0287 | -1.3284 |
| **77** | ITPKB | inositol-trisphosphate 3-kinase B | .0133 | -1.3252 |
| **78** | RPL37 | ribosomal protein L37 | .165 | -1.3250 |
| **79** | PPP1R1B | protein phosphatase 1, regulatory (inhibitor) subunit 1B | .0158 | -1.3242 |
| **80** | TRBV20-1 | T cell receptor beta variable 20-1 | .0276 | -1.3241 |
| **81** | WDR78 | WD repeat domain 78 | .0020 | -1.3233 |
| **82** | ZNF615 | zinc finger protein 615 | .0059 | -1.3201 |
| **83** | CPNE5 | copine V | .0030 | -1.3183 |
| **84** | HIST1H4I | histone cluster 1, H4i | .0257 | -1.3162 |
| **85** | ARMC3 | armadillo repeat containing 3 | .0131 | -1.3147 |
| **86** | SLC47A2 | solute carrier family 47 (multidrug and toxin extrusion), member 2 | .0028 | -1.3146 |
| **87** | PC | pyruvate carboxylase | .0046 | -1.3125 |
| **88** | SPON1 | spondin 1, extracellular matrix protein | .0225 | -1.3123 |
| **89** | DACT2 | dishevelled-binding antagonist of beta-catenin 2 | .0266 | -1.3114 |
| **90** | GPR56 | G protein-coupled receptor 56 | .0013 | -1.3105 |
| **91** | GPR98 | G protein-coupled receptor 98 | .0193 | -1.3086 |
| **92** | APOBEC1 | apolipoprotein B mRNA editing enzyme, catalytic polypeptide 1 | .0114 | -1.3060 |
| **93** | CCDC136 | coiled-coil domain containing 136 | .0238 | -1.3048 |
| **94** | FGFBP2 | fibroblast growth factor binding protein 2 | .0173 | -1.3029 |
| **95** | DAPL1 | death associated protein-like 1 | .0362 | -1.3027 |
| **96** | RGS8 | regulator of G-protein signaling 8 | .0304 | -1.3016 |
| **97** | TC2N | tandem C2 domains, nuclear | .0025 | -1.3009 |
| **98** | MARCH3. | membrane-associated ring finger (C3HC4) 3 | .0011 | -1.3009 |
| **99** | MAPK4 | mitogen-activated protein kinase 4 | .0355 | -1.3001 |
| **100** | SLC6A1 | solute carrier family 6 (neurotransmitter transporter), member 1 | .0223 | -1.2976 |
| **101** | CD24 | CD24 molecule | .0123 | -1.2976 |
| **102** | XIST | X inactive specific transcript (non-protein coding) | .0448 | -1.2974 |
| **103** | TPPP3 | tubulin polymerization-promoting protein family member 3 | .0013 | -1.2969 |
| **104** | DCLK2 | doublecortin-like kinase 2 | .0070 | -1.2966 |
| **105** | PPIP5K1 | diphosphoinositol pentakisphosphate kinase 1 | .0280 | -1.2959 |
| **106** | PNRC1 | proline-rich nuclear receptor coactivator 1 | .0001 | -1.2957 |
| **107** | ZNF239 | zinc finger protein 239 | .0107 | -1.2955 |
| **108** | CLGN | calmegin | .0155 | -1.2948 |
| **109** | TTC9 | tetratricopeptide repeat domain 9 | .0154 | -1.2945 |
| **110** | MRGPRX3 | MAS-related GPR, member X3 | .0227 | -1.2944 |
| **111** | GPD1 | glycerol-3-phosphate dehydrogenase 1 (soluble) | .0030 | -1.2940 |
| **112** | HP08942 | uncharacterized LOC101060544 | .0333 | -1.2936 |
| **113** | ARHGEF2 | Rho/Rac guanine nucleotide exchange factor (GEF) 2 | .0030 | -1.2932 |
| **114** | LDLRAD4 | low density lipoprotein receptor class A domain containing 4 | .0086 | -1.2922 |
| **115** | GADD45G | growth arrest and DNA-damage-inducible, gamma | .0015 | -1.2918 |
| **116** | PCDH19 | protocadherin 19 | .0087 | -1.2902 |
| **117** | RANBP3L | RAN binding protein 3-like | .0007 | -1.2883 |
| **118** | HEY1 | hes-related family bHLH transcription factor with YRPW motif 1 | .0486 | -1.2877 |
| **119** | FCGR2B | Fc fragment of IgG, low affinity IIb, receptor (CD32) | .0196 | -1.2873 |
| **120** | DLG1-AS1 | DLG1 antisense RNA 1 | .0273 | -1.2848 |
| **121** | CD38 | CD38 molecule | .0012 | -1.2846 |
| **122** | ZNRF2 | zinc and ring finger 2 | .0371 | -1.2839 |
| **123** | TAS2R19 | taste receptor, type 2, member 19 | .0271 | -1.2825 |
| **124** | MYH16 | myosin, heavy chain 16 pseudogene | .0158 | -1.2810 |
| **125** | CCDC144A | coiled-coil domain containing 144A | .0072 | -1.2808 |
| **126** | PCDHB9 | protocadherin beta 9 | .0454 | -1.2806 |
| **127** | NBPF20 | neuroblastoma breakpoint family, member 20 | .0195 | -1.2795 |
| **128** | ST18 | suppression of tumorigenicity 18, zinc finger | .0040 | -1.2794 |
| **129** | TMEM161B-AS1 | TMEM161B antisense RNA 1 | .0039 | -1.2752 |
| **130** | TOX3 | TOX high mobility group box family member 3 | .0037 | -1.2732 |
| **131** | FRAT2 | frequently rearranged in advanced T-cell lymphomas 2 | .0259 | -1.2725 |
| **132** | PWARSN | Prader Willi/Angelman region RNA, SNRPN neighbor | .0120 | -1.2720 |
| **133** | KAL1 | Kallmann syndrome 1 sequence | .0038 | -1.2699 |
| **134** | BCL11A | B-cell CLL/lymphoma 11A (zinc finger protein) | .0040 | -1.2682 |
| **135** | HYDIN | HYDIN, axonemal central pair apparatus protein | .0316 | -1.2678 |
| **136** | LRRN2 | leucine rich repeat neuronal 2 | .0074 | -1.2669 |
| **137** | PMP2 | peripheral myelin protein 2 | .0309 | -1.2664 |
| **138** | CAMK2N1 | calcium/calmodulin-dependent protein kinase II inhibitor | .0485 | -1.2646 |
| **139** | BEX1 | brain expressed, X-linked 1 | .0031 | -1.2644 |
| **140** | PRSS2 | protease, serine, 2 (trypsin 2) | .0142 | -1.2637 |
| **141** | C11orf63 | chromosome 11 open reading frame 63 | .0363 | -1.2630 |
| **142** | PAG1 | phosphoprotein membrane anchor with glycosphingolipid 1 | .0440 | -1.2623 |
| **143** | POU5F1P4 | POU class 5 homeobox 1 pseudogene 4 | .0481 | -1.2620 |
| **144** | CRY1 | cryptochrome circadian clock 1 | .0139 | -1.2614 |
| **145** | DLL3 | delta-like 3 (Drosophila) | .0218 | -1.2612 |
| **146** | MICAL1 | microtubule associated monooxygenase, calponin and LIM domain 1 | .0346 | -1.2606 |
| **147** | WDR16 | WD repeat domain 16 | .0138 | -1.2600 |
| **148** | NR2F1 | nuclear receptor subfamily 2, group F, member 1 | .0308 | -1.2596 |
| **149** | FAM110B | family with sequence similarity 110, member B | .0210 | -1.2569 |
| **150** | SNAI1 | snail family zinc finger 1 | .0153 | -1.2567 |
| **151** | PKIA | protein kinase (cAMP-dependent, catalytic) inhibitor alpha | .0023 | -1.2556 |
| **152** | HIST2H2AB | histone cluster 2, H2ab | .0291 | -1.2555 |
| **153** | VN1R108P | vomeronasal 1 receptor 108 pseudogene | .0004 | -1.2554 |
| **154** | FSD1L | fibronectin type III and SPRY domain containing 1-like | .0357 | -1.2549 |
| **155** | TPI1P2 | triosephosphate isomerase 1 pseudogene 2 | .0235 | -1.2547 |
| **156** | ZNF10 | zinc finger protein 10 | .0465 | -1.2540 |
| **157** | PKP1 | plakophilin 1 | .0229 | -1.2529 |
| **158** | ARMC2 | armadillo repeat containing 2 | .0281 | -1.2523 |
| **159** | CRB1 | crumbs family member 1 | .0025 | -1.2522 |
| **160** | TMEM35 | transmembrane protein 35 | .0155 | -1.2518 |
| **161** | HIST1H4D | histone cluster 1, H4d | .0163 | -1.2517 |
| **162** | MAPT | microtubule-associated protein tau | .0214 | -1.2511 |
| **163** | STK32A | serine/threonine kinase 32A | .0130 | -1.2501 |
| **164** | GYG2 | glycogenin 2 | .0116 | -1.2500 |
| **165** | EEF1G | eukaryotic translation elongation factor 1 gamma | .0028 | -1.2500 |
| **166** | CSPG4P13 | chondroitin sulfate proteoglycan 4 pseudogene 13 | .0194 | -1.2492 |
| **167** | HIST1H1B | histone cluster 1, H1b | .0306 | -1.2490 |
| **168** | CBX2 | chromobox homolog 2 | .0041 | -1.2489 |
| **169** | TRAJ38 | T cell receptor alpha joining 38 | .0466 | -1.2486 |
| **170** | SMDT1 | single-pass membrane protein with aspartate-rich tail 1 | .0028 | -1.2484 |
| **171** | SYNE2 | spectrin repeat containing, nuclear envelope 2 | .0417 | -1.2472 |
| **172** | ZNF548 | zinc finger protein 548 | .0195 | -1.2470 |
| **173** | IL31 | interleukin 31 | .0005 | -1.2467 |
| **174** | PPARG | peroxisome proliferator-activated receptor gamma | .0048 | -1.2464 |
| **175** | PIN4P1 | protein NIMA-interacting, 4 pseudogene 1 | .0271 | -1.2463 |
| **176** | CITED1 | Cbp/p300-interacting transactivator, carboxy-terminal domain D1 | .0494 | -1.2458 |
| **177** | FAM212B | family with sequence similarity 212, member B | .0217 | -1.2457 |
| **178** | CA14 | carbonic anhydrase XIV | .0306 | -1.2456 |
| **179** | C12orf45 | chromosome 12 open reading frame 45 | .0040 | -1.2451 |
| **180** | SCARNA7 | small Cajal body-specific RNA 7 | .0162 | -1.2446 |
| **181** | MAP1A | microtubule-associated protein 1A | .0075 | -1.2445 |
| **182** | PGAP1 | post-GPI attachment to proteins 1 | .0138 | -1.2445 |
| **183** | C1orf198 | chromosome 1 open reading frame 198 | .0022 | -1.2442 |
| **184** | ATP2B2 | ATPase, Ca++ transporting, plasma membrane 2 | .0380 | -1.2442 |
| **185** | SPINT1 | serine peptidase inhibitor, Kunitz type 1 | .0083 | -1.2435 |
| **186** | ADAMTS16 | ADAM metallopeptidase with thrombospondin type 1 motif, 16 | .0001 | -1.2434 |
| **187** | SNAP25 | synaptosomal-associated protein, 25kDa | .0019 | -1.2431 |
| **188** | TPRX1 | tetra-peptide repeat homeobox 1 | .0342 | -1.2431 |
| **189** | TRIM2 | tripartite motif containing 2 | .0027 | -1.2421 |
| **190** | ACTR5 | ARP5 actin-related protein 5 homolog (yeast) | .0005 | -1.2417 |
| **191** | IGFBPL1 | insulin-like growth factor binding protein-like 1 | .0292 | -1.2416 |
| **192** | NAPA-AS1 | NAPA antisense RNA 1 | .0095 | -1.2416 |
| **193** | TRIM9 | tripartite motif containing 9 | .0045 | -1.2413 |
| **194** | IFT22 | intraflagellar transport 22 homolog (Chlamydomonas) | .0026 | -1.2406 |
| **195** | GPR155 | G protein-coupled receptor 155 | .0047 | -1.2401 |
| **196** | SLC16A4 | solute carrier family 16, member 4 | .0047 | -1.2392 |
| **197** | FAM66D | family with sequence similarity 66, member D | .0443 | -1.2391 |
| **198** | ZNF474 | zinc finger protein 474 | .0061 | -1.2387 |
| **199** | PKMP3 | pyruvate kinase, muscle pseudogene 3 | .0266 | -1.2382 |
| **200** | PDE3A | phosphodiesterase 3A, cGMP-inhibited | .0021 | -1.2380 |
| **201** | C1QL1 | complement component 1, q subcomponent-like 1 | .0183 | -1.2377 |
| **202** | TMEM130 | transmembrane protein 130 | .0338 | -1.2374 |
| **203** | TRAJ5 | T cell receptor alpha joining 5 | .0173 | -1.2371 |
| **204** | MARCKS | myristoylated alanine-rich protein kinase C substrate | .0010 | -1.2368 |
| **205** | FEZF1-AS1 | FEZF1 antisense RNA 1 | .0133 | -1.2365 |
| **206** | TPST1 | tyrosylprotein sulfotransferase 1 | .0000 | -1.2365 |
| **207** | DNAI1 | dynein, axonemal, intermediate chain 1 | .0035 | -1.2360 |
| **208** | TFPI2 | tissue factor pathway inhibitor 2 | .0062 | -1.2357 |
| **209** | PABPC1L2A | poly(A) binding protein, cytoplasmic 1-like 2A | .0270 | -1.2356 |
| **210** | ACYP1 | acylphosphatase 1, erythrocyte (common) type | .0130 | -1.2355 |
| **211** | ZXDA | zinc finger, X-linked, duplicated A | .0388 | -1.2354 |
| **212** | CMIP | c-Maf inducing protein | .0432 | -1.2352 |
| **213** | TBXAS1 | thromboxane A synthase 1 (platelet) | .0473 | -1.2350 |
| **214** | EXTL2 | exostosin-like glycosyltransferase 2 | .0001 | -1.2350 |
| **215** | IMMP1L | IMP1 inner mitochondrial membrane peptidase-like (S. cerevisiae) | .0095 | -1.2346 |
| **216** | C8orf37 | chromosome 8 open reading frame 37 | .0033 | -1.2340 |
| **217** | SLC15A2 | solute carrier family 15 (oligopeptide transporter), member 2 | .0060 | -1.2340 |
| **218** | ANKRD44 | ankyrin repeat domain 44 | .0038 | -1.2335 |
| **219** | COL1A2 | collagen, type I, alpha 2 | .0261 | -1.2334 |
| **220** | KLHDC8A | kelch domain containing 8A | .0001 | -1.2324 |
| **221** | PLEKHM1 | pleckstrin homology domain containing, family M 1 | .0210 | -1.2313 |
| **222** | SPATA7 | spermatogenesis associated 7 | .0162 | -1.2292 |
| **223** | GATM | glycine amidinotransferase (L-arginine:glycine amidinotransferase) | .0051 | -1.2280 |
| **224** | GLTSCR2 | glioma tumor suppressor candidate region gene 2 | .0023 | -1.2276 |
| **225** | SMOC1 | SPARC related modular calcium binding 1 | .0011 | -1.2276 |
| **226** | ABCC6P2 | ATP-binding cassette, sub-family C, member 6 pseudogene 2 | .0458 | -1.2273 |
| **227** | CDK10 | cyclin-dependent kinase 10 | .0389 | -1.2271 |
| **228** | ENKUR | enkurin, TRPC channel interacting protein | .0024 | -1.2270 |
| **229** | PLXDC2 | plexin domain containing 2 | .0076 | -1.2267 |
| **230** | TAGLN3 | transgelin 3 | .0120 | -1.2264 |
| **231** | TTC33 | tetratricopeptide repeat domain 33 | .0043 | -1.2264 |
| **232** | DST | dystonin | .0438 | -1.2259 |
| **233** | IGLV3-22 | immunoglobulin lambda variable 3-22 (gene/pseudogene) | .0304 | -1.2257 |
| **234** | CCDC177 | coiled-coil domain containing 177 | .0077 | -1.2248 |
| **235** | KCNJ10 | potassium inwardly-rectifying channel, subfamily J, member 10 | .0112 | -1.2247 |
| **236** | SLC9A3R1 | solute carrier family 9, subfamily A member 3, regulator 1 | .0005 | -1.2243 |
| **237** | TTC21B | tetratricopeptide repeat domain 21B | .0065 | -1.2242 |
| **238** | GLUD1 | glutamate dehydrogenase 1 | .0291 | -1.2241 |
| **239** | MPPED2 | metallophosphoesterase domain containing 2 | .0231 | -1.2239 |
| **240** | LGR6 | leucine-rich repeat containing G protein-coupled receptor 6 | .0237 | -1.2230 |
| **241** | LRRC9 | leucine rich repeat containing 9 | .0417 | -1.2217 |
| **242** | LYRM4 | LYR motif containing 4 | .0121 | -1.2215 |
| **243** | RPS4Y2 | ribosomal protein S4, Y-linked 2 | .0109 | -1.2211 |
| **244** | SHF | Src homology 2 domain containing F | .0033 | -1.2202 |
| **245** | MEGF8 | multiple EGF-like-domains 8 | .0024 | -1.2202 |
| **246** | LLGL1 | lethal giant larvae homolog 1 (Drosophila) | .0234 | -1.2196 |
| **247** | GAP43 | growth associated protein 43 | .0183 | -1.2194 |
| **248** | NAT2 | N-acetyltransferase 2 (arylamine N-acetyltransferase) | .0014 | -1.2187 |
| **249** | SMAD9 | SMAD family member 9 | .0004 | -1.2187 |
| **250** | FHDC1 | FH2 domain containing 1 | .0261 | -1.2187 |
| **251** | CNTD2 | cyclin N-terminal domain containing 2 | .0435 | -1.2184 |
| **252** | TCEANC2 | transcription elongation factor A (SII) N-terminal and central domain 2 | .0437 | -1.2184 |
| **253** | GSTM3 | glutathione S-transferase mu 3 (brain) | .0046 | -1.2183 |
| **254** | ELMO1 | engulfment and cell motility 1 | .0128 | -1.2181 |
| **255** | RCBTB2 | regulator of chromosome condensation (RCC1) and BTB (POZ) domain 2 | .0054 | -1.2179 |
| **256** | APC | adenomatous polyposis coli | .0047 | -1.2178 |
| **257** | RPL7P58 | ribosomal protein L7 pseudogene 58 | .0167 | -1.2175 |
| **258** | TSHR | thyroid stimulating hormone receptor | .0219 | -1.2174 |
| **259** | AF015720.3 | novel transcript | .0468 | -1.2173 |
| **260** | IBA57-AS1 | IBA57 antisense RNA 1 (head to head) | .0426 | -1.2171 |
| **261** | ANKFN1 | ankyrin-repeat and fibronectin type III domain containing 1 | .0031 | -1.2170 |
| **262** | GLCCI1 | glucocorticoid induced transcript 1 | .0046 | -1.2168 |
| **263** | LSAMP | limbic system-associated membrane protein | .0018 | -1.2164 |
| **264** | SLC25A16 | solute carrier family 25 (mitochondrial carrier), member 16 | .0229 | -1.2160 |
| **265** | SULT1C4 | sulfotransferase family, cytosolic, 1C, member 4 | .0177 | -1.2159 |
| **266** | SPACA1 | sperm acrosome associated 1 | .0154 | -1.2158 |
| **267** | CEP97 | centrosomal protein 97kDa | .0041 | -1.2157 |
| **268** | FAM81B | family with sequence similarity 81, member B | .0289 | -1.2153 |
| **269** | OGN | osteoglycin | .0235 | -1.2152 |
| **270** | SERPINA10 | serpin peptidase inhibitor, clade A 10 | .0242 | -1.2150 |
| **271** | SEPT4. | septin 4 | .0111 | -1.2145 |
| **272** | PHYHIPL | phytanoyl-CoA 2-hydroxylase interacting protein-like | .0086 | -1.2139 |
| **273** | SLC12A6 | solute carrier family 12 (potassium/chloride transporter), member 6 | .0001 | -1.2137 |
| **274** | DPY19L3 | dpy-19-like 3 (C. elegans) | .0055 | -1.2134 |
| **275** | RAB9A | RAB9A, member RAS oncogene family | .0125 | -1.2134 |
| **276** | PTPN20B | protein tyrosine phosphatase, non-receptor type 20B | .0389 | -1.2134 |
| **277** | GPATCH11 | G patch domain containing 11 | .0291 | -1.2131 |
| **278** | RNF180 | ring finger protein 180 | .0162 | -1.2123 |
| **279** | CROCCP2 | ciliary rootlet coiled-coil, rootletin pseudogene 2 | .0408 | -1.2118 |
| **280** | ROBO1 | roundabout, axon guidance receptor, homolog 1 (Drosophila) | .0087 | -1.2118 |
| **281** | HDAC5 | deacetylase 5 | .0017 | -1.2118 |
| **282** | ZNRF4 | zinc and ring finger 4 | .0031 | -1.2117 |
| **283** | ZC3H12C | zinc finger CCCH-type containing 12C | .0028 | -1.2106 |
| **284** | WDR38 | WD repeat domain 38 | .0297 | -1.2105 |
| **285** | RIMKLA | ribosomal modification protein rimK-like family member A | .0498 | -1.2105 |
| **286** | KIF26B | kinesin family member 26B | .0028 | -1.2105 |
| **287** | SPAG1 | sperm associated antigen 1 | .0093 | -1.2102 |
| **288** | KRTAP19-4 | keratin associated protein 19-4 | .0281 | -1.2096 |
| **289** | CXCL12 | chemokine (C-X-C motif) ligand 12 | .0258 | -1.2095 |
| **290** | LRTOMT | leucine rich transmembrane and O-methyltransferase domain | .0461 | -1.2095 |
| **291** | SERPINE2 | serpin peptidase inhibitor, clade E 2 | .0120 | -1.2093 |
| **292** | INSM1 | insulinoma-associated 1 | .0266 | -1.2091 |
| **293** | WNT8B | wingless-type MMTV integration site family, member 8B | .0406 | -1.2080 |
| **294** | TRIM36-IT1 | TRIM36 intronic transcript 1 (non-protein coding) | .0034 | -1.2079 |
| **295** | CXorf57 | chromosome X open reading frame 57 | .0205 | -1.2075 |
| **296** | TIAM1 | T-cell lymphoma invasion and metastasis 1 | .0220 | -1.2069 |
| **297** | SNN | stannin | .0114 | -1.2066 |
| **298** | EMID1 | EMI domain containing 1 | .0323 | -1.2065 |
| **299** | IGBP1 | immunoglobulin (CD79A) binding protein 1 | .0127 | -1.2064 |
| **300** | LUM | lumican | .0192 | -1.2063 |
| **301** | SIGLEC15 | sialic acid binding Ig-like lectin 15 | .0017 | -1.2059 |
| **302** | IQGAP2 | IQ motif containing GTPase activating protein 2 | .0260 | -1.2056 |
| **303** | PTRHD1 | peptidyl-tRNA hydrolase domain containing 1 | .0354 | -1.2051 |
| **304** | GP5 | glycoprotein V (platelet) | .0331 | -1.2049 |
| **305** | DLGAP2 | discs, large (Drosophila) homolog-associated protein 2 | .0102 | -1.2048 |
| **306** | PCCA | propionyl CoA carboxylase, alpha polypeptide | .0049 | -1.2048 |
| **307** | MAGEC1 | melanoma antigen family C, 1 | .0201 | -1.2037 |
| **308** | SOX4 | SRY (sex determining region Y)-box 4 | .0005 | -1.2035 |
| **309** | ASCL1 | achaete-scute family bHLH transcription factor 1 | .0024 | -1.2031 |
| **310** | CHD3 | chromodomain helicase DNA binding protein 3 | .0142 | -1.2029 |
| **311** | FBXO21 | F-box protein 21 | .0010 | -1.2011 |
| **312** | SNX29 | sorting nexin 29 | .0142 | -1.2010 |
| **313** | PTBP3 | polypyrimidine tract binding protein 3 | .0003 | -1.2007 |
| **314** | TTYH2 | tweety family member 2 | .0168 | 1.2001 |
| **315** | VAV1 | vav 1 guanine nucleotide exchange factor | .0435 | 1.2009 |
| **316** | GFOD1 | glucose-fructose oxidoreductase domain containing 1 | .0455 | 1.2016 |
| **317** | NEFL | neurofilament, light polypeptide | .0036 | 1.2016 |
| **318** | ESRRA | estrogen-related receptor alpha | .0167 | 1.2020 |
| **319** | ANKDD1A | ankyrin repeat and death domain containing 1A | .0040 | 1.2022 |
| **320** | BCL2L12 | BCL2-like 12 (proline rich) | .0469 | 1.2027 |
| **321** | ABHD15 | abhydrolase domain containing 15 | .0482 | 1.2029 |
| **322** | KCNIP1 | Kv channel interacting protein 1 | .0005 | 1.2030 |
| **323** | FPR1 | formyl peptide receptor 1 | .0118 | 1.2039 |
| **324** | MZF1 | myeloid zinc finger 1 | .0313 | 1.2046 |
| **325** | PLEKHG3 | pleckstrin homology domain containing, family G 3 | .0362 | 1.2057 |
| **326** | AFAP1L1 | actin filament associated protein 1-like 1 | .0158 | 1.2059 |
| **327** | SHISA5 | shisa family member 5 | .0024 | 1.2061 |
| **328** | MT-TV | mitochondrially encoded tRNA valine | .0490 | 1.2065 |
| **329** | OOEP-AS1 | OOEP antisense RNA 1 | .0138 | 1.2068 |
| **330** | AP1B1P1 | adaptor-related protein complex 1, beta 1 subunit pseudogene 1 | .0332 | 1.2071 |
| **331** | C2orf27A | chromosome 2 open reading frame 27A | .0133 | 1.2075 |
| **332** | FAM124A | family with sequence similarity 124A | .0199 | 1.2076 |
| **333** | IRF1 | interferon regulatory factor 1 | .0198 | 1.2078 |
| **334** | MICA | MHC class I polypeptide-related sequence A | .0418 | 1.2083 |
| **335** | GARS | glycyl-tRNA synthetase | .0206 | 1.2087 |
| **336** | IGFBP3 | insulin-like growth factor binding protein 3 | .0110 | 1.2091 |
| **337** | ZNF296 | zinc finger protein 296 | .0457 | 1.2105 |
| **338** | MAP3K11 | mitogen-activated protein kinase kinase kinase 11 | .0220 | 1.2107 |
| **339** | MASTL | microtubule associated serine/threonine kinase-like | .0060 | 1.2110 |
| **340** | C3orf20 | chromosome 3 open reading frame 20 | .0241 | 1.2113 |
| **341** | FAM46A | family with sequence similarity 46, member A | .0117 | 1.2121 |
| **342** | CD34 | CD34 molecule | .0030 | 1.2121 |
| **343** | SLC7A6OS | solute carrier family 7, member 6 opposite strand | .0261 | 1.2126 |
| **344** | BHLHE41 | basic helix-loop-helix family, member e41 | .0303 | 1.2156 |
| **345** | OLFML1 | olfactomedin-like 1 | .0369 | 1.2162 |
| **346** | VLDLR | very low density lipoprotein receptor | .0139 | 1.2164 |
| **347** | OR2A2 | olfactory receptor, family 2, subfamily A, member 2 | .0464 | 1.2173 |
| **348** | FGF2 | fibroblast growth factor 2 (basic) | .0018 | 1.2175 |
| **349** | GYLTL1B | glycosyltransferase-like 1B | .0061 | 1.2176 |
| **350** | AJUBA | ajuba LIM protein | .0031 | 1.2183 |
| **351** | MPHOSPH10 | M-phase phosphoprotein 10 | .0327 | 1.2196 |
| **352** | PATL1 | protein associated with topoisomerase II homolog 1 (yeast) | .0019 | 1.2200 |
| **353** | CXorf38 | chromosome X open reading frame 38 | .0256 | 1.2201 |
| **354** | OLFML3 | olfactomedin-like 3 | .0219 | 1.2210 |
| **355** | FJX1 | four jointed box 1 (Drosophila) | .0010 | 1.2212 |
| **356** | PLAUR | plasminogen activator, urokinase receptor | .0065 | 1.2220 |
| **357** | DSTNP2 | destrin (actin depolymerizing factor) pseudogene 2 | .0470 | 1.2226 |
| **358** | ENPP2 | ectonucleotide pyrophosphatase/phosphodiesterase 2 | .0028 | 1.2231 |
| **359** | MMP24 | matrix metallopeptidase 24 (membrane-inserted) | .0476 | 1.2237 |
| **360** | PIDD1 | p53-induced death domain protein 1 | .0272 | 1.2238 |
| **361** | FAM83G | family with sequence similarity 83, member G | .0365 | 1.2245 |
| **362** | MARS | methionyl-tRNA synthetase | .0001 | 1.2247 |
| **363** | THSD1 | thrombospondin, type I, domain containing 1 | .0214 | 1.2261 |
| **364** | ENOX1 | ecto-NOX disulfide-thiol exchanger 1 | .0190 | 1.2263 |
| **365** | TSPYL5 | TSPY-like 5 | .0396 | 1.2265 |
| **366** | CX3CL1 | chemokine (C-X3-C motif) ligand 1 | .0091 | 1.2268 |
| **367** | PAGE1 | P antigen family, member 1 (prostate associated) | .0450 | 1.2274 |
| **368** | HNF4G | hepatocyte nuclear factor 4, gamma | .0342 | 1.2275 |
| **369** | HCG4B | HLA complex group 4B (non-protein coding) | .0494 | 1.2280 |
| **370** | LYPD8 | PLAUR domain containing 8 | .0338 | 1.2281 |
| **371** | WDFY1 | WD repeat and FYVE domain containing 1 | .0070 | 1.2288 |
| **372** | FGF11 | fibroblast growth factor 11 | .0263 | 1.2302 |
| **373** | GBP1P1 | guanylate binding protein 1, interferon-inducible pseudogene 1 | .0495 | 1.2329 |
| **374** | REEP4 | receptor accessory protein 4 | .0075 | 1.2332 |
| **375** | DHX34 | DEAH (Asp-Glu-Ala-His) box polypeptide 34 | .0320 | 1.2355 |
| **376** | EDN1 | endothelin 1 | .0026 | 1.2364 |
| **377** | LILRA3 | leukocyte immunoglobulin-like receptor, subfamily A 3 | .0237 | 1.2390 |
| **378** | DUSP16 | dual specificity phosphatase 16 | .0011 | 1.2392 |
| **379** | RNF31 | ring finger protein 31 | .0220 | 1.2393 |
| **380** | SLC7A5 | solute carrier family 7 (amino acid transporter light chain), member 5 | .0036 | 1.2397 |
| **381** | EHD4 | EH-domain containing 4 | .0106 | 1.2404 |
| **382** | PTK2B | protein tyrosine kinase 2 beta | .0077 | 1.2423 |
| **383** | KCNJ2 | potassium inwardly-rectifying channel, subfamily J, member 2 | .0229 | 1.2438 |
| **384** | IER3 | immediate early response 3 | .0004 | 1.2453 |
| **385** | TRIP13 | thyroid hormone receptor interactor 13 | .0351 | 1.2454 |
| **386** | UNC93B3 | unc-93 homolog B3 pseudogene (C. elegans) | .0167 | 1.2463 |
| **387** | SEC11C | SEC11 homolog C (S. cerevisiae) | .0248 | 1.2473 |
| **388** | C6orf62 | chromosome 6 open reading frame 62 | .0034 | 1.2476 |
| **389** | DPPA2P3 | developmental pluripotency associated 2 pseudogene 3 | .0155 | 1.2476 |
| **390** | UNC93B1 | unc-93 homolog B1 (C. elegans) | .0257 | 1.2485 |
| **391** | BACE2 | beta-site APP-cleaving enzyme 2 | .0105 | 1.2490 |
| **392** | SLCO4A1 | solute carrier organic anion transporter family, member 4 | .0287 | 1.2498 |
| **393** | WHAMM | WAS protein homolog associated with actin, golgi membranes | .0119 | 1.2502 |
| **394** | INHA | inhibin, alpha | .0267 | 1.2508 |
| **395** | PSME1 | proteasome (prosome, macropain) activator subunit 1 | .0003 | 1.2524 |
| **396** | GBP2 | guanylate binding protein 2, interferon-inducible | .0351 | 1.2528 |
| **397** | ELF4 | E74-like factor 4 (ets domain transcription factor) | .0129 | 1.2546 |
| **398** | CX3CR1 | chemokine (C-X3-C motif) receptor 1 | .0236 | 1.2548 |
| **399** | CD6 | CD6 molecule | .0078 | 1.2550 |
| **400** | LARGE | like-glycosyltransferase | .0002 | 1.2562 |
| **401** | MICB | MHC class I polypeptide-related sequence B | .0111 | 1.2563 |
| **402** | OGDHL | oxoglutarate dehydrogenase-like | .0343 | 1.2565 |
| **403** | ADM | adrenomedullin | .0201 | 1.2566 |
| **404** | VCX2 | variable charge, X-linked 2 | .0211 | 1.2581 |
| **405** | AQP2 | aquaporin 2 (collecting duct) | .0256 | 1.2591 |
| **406** | ZNF517 | zinc finger protein 517 | .0277 | 1.2593 |
| **407** | C14orf159 | chromosome 14 open reading frame 159 | .0008 | 1.2602 |
| **408** | ASNSP1 | asparagine synthetase pseudogene 1 | .0003 | 1.2627 |
| **409** | EBI3 | Epstein-Barr virus induced 3 | .0204 | 1.2633 |
| **410** | FAM111A | family with sequence similarity 111, member A | .0117 | 1.2642 |
| **411** | MT-TF | mitochondrially encoded tRNA phenylalanine | .0044 | 1.2642 |
| **412** | LRRC2 | leucine rich repeat containing 2 | .0037 | 1.2649 |
| **413** | GOLGA6L10 | golgin A6 family-like 10 | .0096 | 1.2670 |
| **414** | FOSL1 | FOS-like antigen 1 | .0396 | 1.2676 |
| **415** | ITIH4 | inter-alpha-trypsin inhibitor heavy chain family, member 4 | .0430 | 1.2689 |
| **416** | VCAM1 | vascular cell adhesion molecule 1 | .0099 | 1.2696 |
| **417** | POLR3G | polymerase (RNA) III (DNA directed) polypeptide G (32kD) | .0070 | 1.2726 |
| **418** | SLC47A1 | solute carrier family 47 (multidrug and toxin extrusion), membeber 1 | .0056 | 1.2735 |
| **419** | ALX3 | ALX homeobox 3 | .0459 | 1.2736 |
| **420** | CD68 | CD68 molecule | .0020 | 1.2739 |
| **421** | MMP2 | matrix metallopeptidase 2 | .0003 | 1.2745 |
| **422** | REPIN1 | replication initiator 1 | .0022 | 1.2762 |
| **423** | KCNQ2 | potassium voltage-gated channel, KQT-like subfamily, member 2 | .0005 | 1.2768 |
| **424** | IGHV7-81 | immunoglobulin heavy variable 7-81 (non-functional) | .0232 | 1.2784 |
| **425** | CLDN1 | claudin 1 | .0294 | 1.2798 |
| **426** | MET | MET proto-oncogene, receptor tyrosine kinase | .0069 | 1.2805 |
| **427** | DNM1P46 | DNM1 pseudogene 46 | .0470 | 1.2834 |
| **428** | C3orf62 | chromosome 3 open reading frame 62 | .0396 | 1.2858 |
| **429** | TGFA | transforming growth factor, alpha | .0166 | 1.2894 |
| **430** | CCND1 | cyclin D1 | .0020 | 1.2927 |
| **431** | CYP4A22-AS1 | CYP4A22 antisense RNA 1 | .0325 | 1.2929 |
| **432** | ZNF302 | zinc finger protein 302 | .0271 | 1.2937 |
| **433** | GTF3C2-AS1 | GTF3C2 antisense RNA 1 | .0491 | 1.2937 |
| **434** | PHF11 | PHD finger protein 11 | .0036 | 1.2949 |
| **435** | TRIM6 | tripartite motif containing 6 | .0176 | 1.2951 |
| **436** | MROH2A | maestro heat-like repeat family member 2A | .0018 | 1.2955 |
| **437** | BANCR | BRAF-activated non-protein coding RNA | .0174 | 1.2980 |
| **438** | SOCS1 | suppressor of cytokine signaling 1 | .0128 | 1.3006 |
| **439** | KLK10 | kallikrein-related peptidase 10 | .0082 | 1.3047 |
| **440** | KRT80 | keratin 80 | .0425 | 1.3061 |
| **441** | HES7 | hes family bHLH transcription factor 7 | .0085 | 1.3092 |
| **442** | DUSP4 | dual specificity phosphatase 4 | .0178 | 1.3100 |
| **443** | RTP4 | receptor (chemosensory) transporter protein 4 | .0177 | 1.3109 |
| **444** | PDLIM1 | PDZ and LIM domain 1 | .0001 | 1.3114 |
| **445** | CCRN4L | CCR4 carbon catabolite repression 4-like (S. cerevisiae) | .0002 | 1.3116 |
| **446** | FLT3LG | fms-related tyrosine kinase 3 ligand | .0012 | 1.3131 |
| **447** | H1F0 | H1 histone family, member 0 | .0228 | 1.3138 |
| **448** | LGALS14 | lectin, galactoside-binding, soluble, 14 | .0194 | 1.3143 |
| **449** | TRAJ18 | T cell receptor alpha joining 18 | .0067 | 1.3144 |
| **450** | RBMXL3 | RNA binding motif protein, X-linked-like 3 | .0049 | 1.3152 |
| **451** | AKAP3 | A kinase (PRKA) anchor protein 3 | .0102 | 1.3155 |
| **452** | CASP1 | caspase 1, apoptosis-related cysteine peptidase | .0034 | 1.3156 |
| **453** | TAPBPL | TAP binding protein-like | .0229 | 1.3174 |
| **454** | CHAC1 | ChaC, cation transport regulator homolog 1 (E. coli) | .0007 | 1.3191 |
| **455** | NUP210 | nucleoporin 210kDa | .0014 | 1.3196 |
| **456** | C1QL3 | complement component 1, q subcomponent-like 3 | .0029 | 1.3211 |
| **457** | CPZ | carboxypeptidase Z | .0087 | 1.3227 |
| **458** | NAMPT | nicotinamide phosphoribosyltransferase | .0020 | 1.3229 |
| **459** | TNFAIP2 | tumor necrosis factor, alpha-induced protein 2 | .0009 | 1.3250 |
| **460** | TGFBR3L | transforming growth factor, beta receptor III-like | .0060 | 1.3252 |
| **461** | GPX8 | glutathione peroxidase 8 (putative) | .0382 | 1.3294 |
| **462** | HERC2P9 | hect domain and RLD 2 pseudogene 9 | .0250 | 1.3359 |
| **463** | SQRDL | sulfide quinone reductase-like (yeast) | .0076 | 1.3370 |
| **464** | NFKBIA | nuclear factor of kappa light polypeptide gene enhancer in B-cells | .0007 | 1.3385 |
| **465** | TMEM62 | transmembrane protein 62 | .0005 | 1.3409 |
| **466** | FBXO9 | F-box protein 9 | .0298 | 1.3425 |
| **467** | PAQR5 | progestin and adipoQ receptor family member V | .0424 | 1.3439 |
| **468** | ASNS | asparagine synthetase (glutamine-hydrolyzing) | .0132 | 1.3491 |
| **469** | KRT24 | keratin 24 | .0348 | 1.3498 |
| **470** | RASGRP3 | RAS guanyl releasing protein 3 | .0009 | 1.3504 |
| **471** | NFE2L3 | nuclear factor, erythroid 2-like 3 | .0012 | 1.3528 |
| **472** | TAPBP | TAP binding protein (tapasin) | .0000 | 1.3533 |
| **473** | KRT72 | keratin 72 | .0427 | 1.3569 |
| **474** | PKD1 | polycystic kidney disease 1 (autosomal dominant) | .0115 | 1.3623 |
| **475** | TRIM78P | tripartite motif containing 78, pseudogene | .0120 | 1.3624 |
| **476** | TRIM5 | tripartite motif containing 5 | .0037 | 1.3648 |
| **477** | KRT71 | keratin 71 | .0259 | 1.3661 |
| **478** | THEMIS2 | thymocyte selection associated family member 2 | .0005 | 1.3701 |
| **479** | C1R | complement component 1, r subcomponent | .0042 | 1.3703 |
| **480** | HIST2H2BF | histone cluster 2, H2bf | .0255 | 1.3750 |
| **481** | C2 | complement component 2 | .0116 | 1.3772 |
| **482** | C1S | complement component 1, s subcomponent | .0006 | 1.3823 |
| **483** | CHRNA1 | cholinergic receptor, nicotinic, alpha 1 (muscle) | .0023 | 1.3853 |
| **484** | RBCK1 | RanBP-type and C3HC4-type zinc finger containing 1 | .0016 | 1.3867 |
| **485** | TMEM140 | transmembrane protein 140 | .0281 | 1.3895 |
| **486** | CCR1 | chemokine (C-C motif) receptor 1 | .0066 | 1.3896 |
| **487** | PANDAR | promoter of CDKN1A antisense DNA damage activated RNA | .0110 | 1.3902 |
| **488** | PSMB8-AS1 | SMB8 antisense RNA 1 (head to head) | .0001 | 1.3910 |
| **489** | DRD4 | dopamine receptor D4 | .0053 | 1.3965 |
| **490** | SLC1A5 | solute carrier family 1 (neutral amino acid transporter), member 5 | .0005 | 1.4017 |
| **491** | TNFAIP3 | tumor necrosis factor, alpha-induced protein 3 | .0029 | 1.4021 |
| **492** | PARP10 | poly (ADP-ribose) polymerase family, member 10 | .0030 | 1.4024 |
| **493** | STAP2 | signal transducing adaptor family member 2 | .0249 | 1.4031 |
| **494** | CD180 | CD180 molecule | .0008 | 1.4035 |
| **495** | USP29 | ubiquitin specific peptidase 29 | .0177 | 1.4052 |
| **496** | APOBEC3D | apolipoprotein B mRNA editing enzyme, catalytic subunit 3D | .0202 | 1.4120 |
| **497** | PARP6 | poly (ADP-ribose) polymerase family, member 6 | .0004 | 1.4156 |
| **498** | CFB | complement factor B | .0070 | 1.4191 |
| **499** | ALPPL2 | alkaline phosphatase, placental-like 2 | .0043 | 1.4307 |
| **500** | PLEKHA6 | pleckstrin homology domain containing, family A member 6 | .0234 | 1.4430 |
| **501** | TUNAR | TCL1 upstream neural differentiation-associated RNA | .0007 | 1.4503 |
| **502** | OGFR | opioid growth factor receptor | .0003 | 1.4504 |
| **503** | NPIPA5 | nuclear pore complex interacting protein family, member A5 | .0230 | 1.4588 |
| **504** | PKD1P1 | polycystic kidney disease 1 (autosomal dominant) pseudogene 1 | .0105 | 1.4600 |
| **505** | MT2A | metallothionein 2A | .0010 | 1.4731 |
| **506** | IGKV2D-24 | immunoglobulin kappa variable 2D-24 (non-functional) | .0466 | 1.4751 |
| **507** | CPM | carboxypeptidase M | .0131 | 1.4770 |
| **508** | AGTRAP | angiotensin II receptor-associated protein | .0000 | 1.4810 |
| **509** | GMPR | guanosine monophosphate reductase | .0076 | 1.4817 |
| **510** | ICAM1 | intercellular adhesion molecule 1 | .0000 | 1.4967 |
| **511** | MDM2 | MDM2 proto-oncogene, E3 ubiquitin protein ligase | .0414 | 1.5096 |
| **512** | IFI30 | interferon, gamma-inducible protein 30 | .0013 | 1.5198 |
| **513** | NPIPA3 | nuclear pore complex interacting protein family, member A3 | .0190 | 1.5242 |
| **514** | TNFSF10 | tumor necrosis factor (ligand) superfamily, member 10 | .0002 | 1.5259 |
| **515** | SLC15A3 | solute carrier family 15 (oligopeptide transporter), member 3 | .0027 | 1.5259 |
| **516** | SECTM1 | secreted and transmembrane 1 | .0013 | 1.5310 |
| **517** | NPIPA1 | nuclear pore complex interacting protein family, member A1 | .0239 | 1.5323 |
| **518** | ERAP2 | endoplasmic reticulum aminopeptidase 2 | .0001 | 1.5409 |
| **519** | HTR6 | 5-hydroxytryptamine (serotonin) receptor 6, G protein-coupled | .0075 | 1.5559 |
| **520** | CSAG3 | CSAG family, member 3 | .0035 | 1.5610 |
| **521** | C3 | complement component 3 | .0006 | 1.5708 |
| **522** | MT-TP | mitochondrially encoded tRNA proline | .0004 | 1.6007 |
| **523** | APOL2 | apolipoprotein L, 2 | .0041 | 1.6221 |
| **524** | ECH1 | enoyl CoA hydratase 1, peroxisomal | .0283 | 1.7217 |
| **525** | ZCCHC2 | zinc finger, CCHC domain containing 2 | .0000 | 1.7223 |
| **526** | CXCL10 | chemokine (C-X-C motif) ligand 10 | .0061 | 1.7347 |
| **527** | KRT75 | keratin 75 | .0024 | 1.7402 |
| **528** | ITGA11 | integrin, alpha 11 | .0012 | 1.9056 |
| **529** | SERPING1 | serpin peptidase inhibitor, clade G 1 | .0018 | 2.0229 |
| **530** | GBP3 | guanylate binding protein 3 | .0006 | 2.0516 |
