# Supplementary material for: Interferon-Alpha Reduces Human Hippocampal Neurogenesis and Increases Apoptosis via Activation of Distinct STAT1-Dependent Mechanisms
Source: Int J Neuropsychopharmacol. 2017 Oct 10;21(2):187–200. doi: 10.1093/ijnp/pyx083 (PMC5793815; doi:10.1093/ijnp/pyx083)
Supplement: Supplementary Table 3 [file pyx083_suppl_supplementary_table_3.docx]

**Supplementary Table 3. Genes Commonly Modulated by IFN-α 500 pg/mL IFN-α 5000 pg/mL When Compared with Vehicle**

| **Elements in common between IFN-α 500 pg/mL (vs vehicle) and IFN-α 5000 pg/mL (vs vehicle)** | | | | |  |  |
| --- | --- | --- | --- | --- | --- | --- |
|  |  |  |  |  |  |  |
|  | **Gene** | **Gene title** | **IFN-α 500 pg/mL** | | **IFN-α 5000 pg/mL** | |
|  |  |  | ***P*** | **FC** | ***P*** | **FC** |
| **1** | ACO1 | aconitase 1, soluble | .0005 | -1.2539 | .0000 | -1.6126 |
| **2** | ACSL5 | acyl-CoA synthetase long-chain family member 5 | .0488 | 1.3648 | .0043 | 1.7526 |
| **3** | ADAMTS15 | ADAM metallopeptidase with thrombospondin type 1 motif, 15 | .0004 | -1.3701 | .0038 | -1.2238 |
| **4** | ADAMTS9 | ADAM metallopeptidase with thrombospondin type 1 motif, 9 | .0242 | 1.2561 | .0036 | 1.4207 |
| **5** | ADAR | adenosine deaminase, RNA-specific | .0000 | 1.4422 | .0000 | 1.6058 |
| **6** | AGRN | agrin | .0396 | 1.2005 | .0076 | 1.3170 |
| **7** | ALOX5 | arachidonate 5-lipoxygenase | .0006 | 1.5504 | .0001 | 1.9468 |
| **8** | AMBN | ameloblastin (enamel matrix protein) | .0092 | -1.2003 | .0001 | -1.5116 |
| **9** | ANKRD34B | ankyrin repeat domain 34B | .0027 | -1.3883 | .0036 | -1.3626 |
| **10** | APOL6 | apolipoprotein L, 6 | .0044 | 1.6836 | .0001 | 3.0540 |
| **11** | APOPT1 | apoptogenic 1, mitochondrial | .0038 | -1.2128 | .0020 | -1.2469 |
| **12** | ASPA | aspartoacylase | .0317 | -1.3282 | .0197 | -1.3786 |
| **13** | ATP10B | ATPase, class V, type 10B | .0123 | -1.2768 | .0066 | -1.3253 |
| **14** | ATP5SL | ATP5S-like | .0102 | 1.3991 | .0472 | 1.2545 |
| **15** | B2M | beta-2-microglobulin | .0027 | 1.2616 | .0001 | 1.5171 |
| **16** | B3GALNT1 | beta-1,3-N-acetylgalactosaminyltransferase 1 | .0054 | 1.2367 | .0045 | 1.2481 |
| **17** | BRINP1 | bone morphogenetic protein/retinoic acid inducible 1 | .0284 | -1.2500 | .0007 | -1.6313 |
| **18** | BST2 | bone marrow stromal cell antigen 2 | .0000 | 3.4287 | .0000 | 4.7511 |
| **19** | C14orf37 | chromosome 14 open reading frame 37 | .0005 | -1.2861 | .0000 | -1.6469 |
| **20** | C19orf40 | chromosome 19 open reading frame 40 | .0318 | 1.2093 | .0230 | 1.2299 |
| **21** | C19orf66 | chromosome 19 open reading frame 66 | .0000 | 1.7292 | .0000 | 2.3800 |
| **22** | C2orf50 | chromosome 2 open reading frame 50 | .0289 | 1.3091 | .0256 | 1.3205 |
| **23** | C5orf56 | chromosome 5 open reading frame 56 | .0003 | 1.3167 | .0001 | 1.4310 |
| **24** | CAPN6 | calpain 6 | .0044 | -1.4791 | .0004 | -1.8819 |
| **25** | CAPS | calcyphosine | .0049 | -1.4256 | .0015 | -1.5683 |
| **26** | CASC18 | cancer susceptibility candidate 18 | .0004 | -1.2482 | .0002 | -1.2827 |
| **27** | CDC14B | cell division cycle 14B | .0004 | -1.3659 | .0007 | -1.3372 |
| **28** | CDS1 | CDP-diacylglycerol synthase 1 | .0224 | -1.2011 | .0193 | -1.2096 |
| **29** | CLEC19A | C-type lectin domain family 19, member A | .0009 | -1.4745 | .0003 | -1.6205 |
| **30** | CMPK2 | cytidine monophosphate (UMP-CMP) kinase 2 | .0000 | 2.0960 | .0000 | 5.0366 |
| **31** | CNR1 | cannabinoid receptor 1 (brain) | .0243 | 1.2636 | .0036 | 1.4347 |
| **32** | COL8A1 | collagen, type VIII, alpha 1 | .0164 | -1.2692 | .0294 | -1.2280 |
| **33** | CRMP1 | collapsin response mediator protein 1 | .0018 | -1.3101 | .0001 | -1.6487 |
| **34** | CXCL16 | chemokine (C-X-C motif) ligand 16 | .0012 | 1.2351 | .0000 | 1.6389 |
| **35** | DAGLA | diacylglycerol lipase, alpha | .0461 | 1.2565 | .0335 | 1.2843 |
| **36** | DANCR | differentiation antagonizing non-protein coding RNA | .0041 | -1.3461 | .0009 | -1.4966 |
| **37** | DDX26B-AS1 | DDX26B antisense RNA 1 | .0101 | -1.2716 | .0098 | -1.2741 |
| **38** | DDX58 | DEAD (Asp-Glu-Ala-Asp) box polypeptide 58 | .0000 | 2.4229 | .0000 | 3.4534 |
| **39** | DDX60 | DEAD (Asp-Glu-Ala-Asp) box polypeptide 60 | .0000 | 3.1126 | .0000 | 4.1705 |
| **40** | DDX60L | DEAD (Asp-Glu-Ala-Asp) box polypeptide 60-like | .0000 | 1.7868 | .0000 | 2.9599 |
| **41** | DGCR5 | DiGeorge syndrome critical region gene 5 | .0471 | 1.2311 | .0483 | -1.2063 |
| **42** | DHX58 | DEXH (Asp-Glu-X-His) box polypeptide 58 | .0291 | 1.2458 | .0003 | 1.7892 |
| **43** | DOC2A | double C2-like domains, alpha | .0049 | 1.2594 | .0120 | 1.2077 |
| **44** | DTX3L | deltex 3 like, E3 ubiquitin ligase | .0000 | 1.8187 | .0000 | 2.1390 |
| **45** | EIF2AK2 | eukaryotic translation initiation factor 2-alpha kinase 2 | .0001 | 1.7434 | .0000 | 1.9347 |
| **46** | ELFN1 | extracellular leucine-rich repeat and fibronectin type III | .0014 | 1.4152 | .0004 | 1.5632 |
| **47** | ERAP1 | endoplasmic reticulum aminopeptidase 1 | .0000 | 1.3315 | .0000 | 1.5561 |
| **48** | ESPN | espin | .0152 | -1.2515 | .0298 | -1.2081 |
| **49** | ETV7 | ets variant 7 | .0081 | 1.2045 | .0002 | 1.4807 |
| **50** | FAM69C | family with sequence similarity 69, member C | .0190 | -1.2242 | .0004 | -1.5670 |
| **51** | FAR2 | fatty acyl CoA reductase 2 | .0144 | 1.2072 | .0007 | 1.4252 |
| **52** | FBLIM1 | filamin binding LIM protein 1 | .0006 | -1.2221 | .0001 | -1.3502 |
| **53** | FBXO6 | F-box protein 6 | .0043 | 1.3307 | .0003 | 1.6299 |
| **54** | FEZF1 | FEZ family zinc finger 1 | .0057 | -1.3439 | .0071 | -1.3259 |
| **55** | GACAT2 | gastric cancer associated transcript 2 (non-protein coding) | .0436 | -1.2516 | .0028 | -1.5331 |
| **56** | GBP1 | guanylate binding protein 1, interferon-inducible | .0014 | 1.5760 | .0000 | 2.7362 |
| **57** | GNG3 | guanine nucleotide binding protein (G protein), gamma 3 | .0055 | -1.3444 | .0028 | -1.4067 |
| **58** | H19 | H19, imprinted maternally expressed transcript | .0143 | -1.2891 | .0004 | -1.7145 |
| **59** | HCG27 | HLA complex group 27 (non-protein coding) | .0191 | 1.3388 | .0413 | 1.2281 |
| **60** | HCP5 | HLA complex P5 (non-protein coding) | .0048 | 1.4681 | .0002 | 2.0001 |
| **61** | HELZ2 | helicase with zinc finger 2, transcriptional coactivator | .0053 | 1.4933 | .0003 | 2.0145 |
| **62** | HERC5 | HECT and RLD domain containing E3 ubiquitin protein ligase 5 | .0000 | 4.2651 | .0000 | 9.5901 |
| **63** | HERC6 | HECT and RLD domain containing E3 ubiquitin protein ligase 6 | .0000 | 5.1428 | .0000 | 7.3100 |
| **64** | HIST1H4H | histone cluster 1, H4h | .0141 | -1.3206 | .0281 | -1.2635 |
| **65** | HIST1H4L | histone cluster 1, H4l | .0095 | -1.3376 | .0043 | -1.4135 |
| **66** | HLA-A | major histocompatibility complex, class I, A | .0088 | 1.3165 | .0000 | 1.4497 |
| **67** | HLA-B | major histocompatibility complex, class I, B | .0020 | 1.2798 | .0087 | 1.4906 |
| **68** | HLA-C | major histocompatibility complex, class I, C | .0036 | 1.2311 | .0001 | 1.4904 |
| **69** | HLA-DQA1 | major histocompatibility complex, class II, DQ alpha 1 | .0029 | -1.2054 | .0023 | -1.2161 |
| **70** | HLA-E | major histocompatibility complex, class I, E | .0002 | 1.3170 | .0000 | 1.4655 |
| **71** | HLA-F | major histocompatibility complex, class I, F | .0334 | 1.2081 | .0015 | 1.5117 |
| **72** | HLA-G | major histocompatibility complex, class I, G | .0000 | 1.2122 | .0000 | 1.3427 |
| **73** | HLA-L | major histocompatibility complex, class I, L (pseudogene) | .0032 | 1.5773 | .0009 | 1.8063 |
| **74** | HSD17B14 | hydroxysteroid (17-beta) dehydrogenase 14 | .0358 | -1.3025 | .0378 | -1.2973 |
| **75** | IFI16 | interferon, gamma-inducible protein 16 | .0001 | 1.3184 | .0000 | 1.4212 |
| **76** | IFI27 | interferon, alpha-inducible protein 27 | .0000 | 5.8118 | .0000 | 8.9488 |
| **77** | IFI35 | interferon-induced protein 35 | .0000 | 1.6937 | .0000 | 2.3638 |
| **78** | IFI44 | interferon-induced protein 44 | .0000 | 2.9721 | .0000 | 5.0064 |
| **79** | IFI44L | interferon-induced protein 44-like | .0000 | 2.7889 | .0000 | 3.5954 |
| **80** | IFI6 | interferon, alpha-inducible protein 6 | .0000 | 1.6726 | .0000 | 1.9137 |
| **81** | IFIH1 | interferon induced with helicase C domain 1 | .0000 | 2.4741 | .0000 | 3.7995 |
| **82** | IFIT1 | interferon-induced protein with tetratricopeptide repeats 1 | .0000 | 3.9687 | .0000 | 5.9327 |
| **83** | IFIT2 | interferon-induced protein with tetratricopeptide repeats 2 | .0101 | 1.3058 | .0001 | 1.9393 |
| **84** | IFIT3 | interferon-induced protein with tetratricopeptide repeats 3 | .0000 | 4.5061 | .0000 | 6.7023 |
| **85** | IFIT5 | interferon-induced protein with tetratricopeptide repeats 5 | .0000 | 2.0472 | .0000 | 2.7849 |
| **86** | IFITM1 | interferon induced transmembrane protein 1 | .0000 | 1.5383 | .0000 | 1.8268 |
| **87** | IFITM2 | interferon induced transmembrane protein 2 | .0116 | 1.2423 | .0007 | 1.4769 |
| **88** | IFITM3 | interferon induced transmembrane protein 3 | .0005 | 1.3325 | .0002 | 1.4276 |
| **89** | IFITM4P | interferon induced transmembrane protein 4 pseudogene | .0360 | 1.3224 | .0001 | 2.2261 |
| **90** | IGFBP5 | insulin-like growth factor binding protein 5 | .0097 | -1.3044 | .0206 | -1.2488 |
| **91** | IGHV1-58 | immunoglobulin heavy variable 1-58 | .0470 | 1.3584 | .0384 | 1.3838 |
| **92** | IGLV3-16 | immunoglobulin lambda variable 3-16 | .0364 | 1.4011 | .0372 | 1.3984 |
| **93** | IRF7 | interferon regulatory factor 7 | .0013 | 1.4297 | .0000 | 2.0507 |
| **94** | IRF9 | interferon regulatory factor 9 | .0000 | 1.7799 | .0000 | 1.9109 |
| **95** | ISG15 | ISG15 ubiquitin-like modifier | .0000 | 2.2637 | .0000 | 3.4895 |
| **96** | KB-1107E3.1 | novel transcript | .0035 | 1.2614 | .0052 | 1.2372 |
| **97** | KBTBD3 | kelch repeat and BTB (POZ) domain containing 3 | .0113 | -1.2939 | .0038 | -1.3870 |
| **98** | KRT78 | keratin 78 | .0237 | -1.3323 | .0161 | -1.3713 |
| **99** | LA16c-23H5 | LA16c-23H5.4 // novel transcript | .0161 | -1.3708 | .0083 | -1.4444 |
| **100** | LAP3 | leucine aminopeptidase 3 | .0001 | 1.4705 | .0000 | 1.8712 |
| **101** | LGALS3BP | lectin, galactoside-binding, soluble, 3 binding protein | .0000 | 1.3978 | .0000 | 1.5323 |
| **102** | LPXN | leupaxin | .0052 | -1.3475 | .0193 | -1.2477 |
| **103** | LRTM1 | leucine-rich repeats and transmembrane domains 1 | .0122 | 1.3307 | .0099 | 1.3501 |
| **104** | LY6E | lymphocyte antigen 6 complex, locus E | .0009 | 1.4221 | .0000 | 1.9659 |
| **105** | MAGEB16 | melanoma antigen family B, 16 | .0238 | 1.2043 | .0129 | 1.2412 |
| **106** | MFAP2 | microfibrillar-associated protein 2 | .0085 | -1.3346 | .0110 | -1.3132 |
| **107** | MFAP5 | microfibrillar associated protein 5 | .0009 | -1.4425 | .0151 | -1.2267 |
| **108** | MOV10 | Mov10, Moloney leukemia virus 10, homolog (mouse) | .0008 | 1.2031 | .0000 | 1.5627 |
| **109** | MT1CP | metallothionein 1C, pseudogene | .0323 | 1.2763 | .0032 | 1.5174 |
| **110** | MT1F | metallothionein 1F | .0120 | 1.3267 | .0063 | 1.3874 |
| **111** | MX1 | myxovirus (influenza virus) resistance 1 | .0000 | 3.4770 | .0000 | 4.9116 |
| **112** | MX2 | myxovirus (influenza virus) resistance 2 (mouse) | .0046 | 1.4066 | .0000 | 2.2792 |
| **113** | NLRC5 | NLR family, CARD domain containing 5 | .0001 | 1.3049 | .0000 | 1.7724 |
| **114** | NMI | N-myc (and STAT) interactor | .0093 | 1.5819 | .0006 | 2.2210 |
| **115** | NNMT | nicotinamide N-methyltransferase | .0241 | -1.2137 | .0061 | -1.3058 |
| **116** | NPTX2 | neuronal pentraxin II | .0200 | -1.2303 | .0037 | -1.3542 |
| **117** | NR4A3 | nuclear receptor subfamily 4, group A, member 3 | .0032 | 1.2599 | .0004 | 1.4104 |
| **118** | NT5E | 5-nucleotidase, ecto (CD73) | .0000 | 2.5283 | .0000 | 5.3562 |
| **119** | NXNL1 | nucleoredoxin-like 1 | .0141 | 1.2319 | .0133 | 1.2356 |
| **120** | OAS1 | 2-5-oligoadenylate synthetase 1, 40/46kDa | .0000 | 4.0275 | .0000 | 6.1462 |
| **121** | OAS2 | 2-5-oligoadenylate synthetase 2, 69/71kDa | .0000 | 5.9169 | .0000 | 9.5578 |
| **122** | OAS3 | 2-5-oligoadenylate synthetase 3, 100kDa | .0000 | 3.0732 | .0000 | 5.3823 |
| **123** | OASL | 2-5-oligoadenylate synthetase-like | .0001 | 1.5931 | .0000 | 3.1111 |
| **124** | PARP12 | poly (ADP-ribose) polymerase family, member 12 | .0000 | 2.0394 | .0000 | 2.8551 |
| **125** | PARP14 | poly (ADP-ribose) polymerase family, member 14 | .0000 | 2.2509 | .0000 | 2.8087 |
| **126** | PARP9 | poly (ADP-ribose) polymerase family, member 9 | .0000 | 1.7170 | .0000 | 1.9867 |
| **127** | PCDH18 | protocadherin 18 | .0030 | -1.2033 | .0023 | -1.2146 |
| **128** | PCDHB3 | protocadherin beta 3 | .0234 | -1.2477 | .0041 | -1.3906 |
| **129** | PDCD1LG2 | programmed cell death 1 ligand 2 | .0453 | 1.3479 | .0071 | 1.6078 |
| **130** | PGAM2 | phosphoglycerate mutase 2 (muscle) | .0239 | -1.2473 | .0018 | -1.4808 |
| **131** | PIRT | phosphoinositide-interacting regulator of receptor channels | .0402 | -1.3411 | .0040 | -1.6636 |
| **132** | PLA2G2F | phospholipase A2, group IIF | .0175 | 1.2268 | .0239 | 1.2083 |
| **133** | PLCH2 | phospholipase C, eta 2 | .0186 | -1.2294 | .0000 | 1.2337 |
| **134** | PLN | phospholamban | .0113 | -1.4861 | .0045 | -1.6234 |
| **135** | PLSCR1 | phospholipid scramblase 1 | .0004 | 1.6421 | .0000 | 2.0842 |
| **136** | PML | promyelocytic leukemia | .0016 | 1.3296 | .0001 | 1.6179 |
| **137** | PNPT1 | polyribonucleotide nucleotidyltransferase 1 | .0011 | 1.2901 | .0000 | 1.6812 |
| **138** | PPM1K | protein phosphatase, Mg2+/Mn2+ dependent, 1K | .0003 | 1.7138 | .0000 | 2.2608 |
| **139** | PRSS35 | protease, serine, 35 | .0228 | -1.3022 | .0081 | -1.4021 |
| **140** | PSMB8 | proteasome (prosome, macropain) subunit, beta type, 8 | .0053 | 1.3056 | .0002 | 1.6960 |
| **141** | PSMB9 | proteasome (prosome, macropain) subunit, beta type, 9 | .0463 | 1.3505 | .0000 | 2.2142 |
| **142** | PSME2 | proteasome (prosome, macropain) activator subunit 2 | .0033 | 1.2253 | .0000 | 1.6183 |
| **143** | RAB13 | RAB13, member RAS oncogene family | .0293 | -1.2203 | .0333 | -1.2119 |
| **144** | RARG | retinoic acid receptor, gamma | .0331 | -1.2717 | .0158 | -1.3375 |
| **145** | RARRES3 | retinoic acid receptor responder (tazarotene induced) 3 | .0363 | 1.2190 | .0013 | 1.5179 |
| **146** | RNF213 | ring finger protein 213 | .0032 | 1.2386 | .0001 | 1.5579 |
| **147** | RPS3A | ribosomal protein S3A | .0320 | 1.2369 | .0151 | 1.2938 |
| **148** | RSAD2 | radical S-adenosyl methionine domain containing 2 | .0000 | 2.9648 | .0000 | 8.5718 |
| **149** | SAMD9 | sterile alpha motif domain containing 9 | .0000 | 2.2459 | .0000 | 3.6705 |
| **150** | SAMD9L | sterile alpha motif domain containing 9-like | .0000 | 1.9238 | .0000 | 2.7190 |
| **151** | SAMHD1 | SAM domain and HD domain 1 | .0000 | 1.8799 | .0000 | 2.6031 |
| **152** | SERPINA2 | serpin peptidase inhibitor, clade A, member 2 | .0164 | -1.2384 | .0079 | -1.2885 |
| **153** | SERTAD4 | SERTA domain containing 4 | .0052 | -1.3425 | .0106 | -1.2868 |
| **154** | SFMBT1 | Scm-like with four mbt domains 1 | .0039 | 1.2181 | .0052 | 1.2037 |
| **155** | SH2D2A | SH2 domain containing 2A | .0081 | 1.4800 | .0288 | 1.3340 |
| **156** | SKA1 | spindle and kinetochore associated complex subunit 1 | .0169 | 1.2558 | .0172 | 1.2545 |
| **157** | SLC16A14 | solute carrier family 16, member 14 | .0116 | 1.3151 | .0139 | 1.3002 |
| **158** | SLC27A2 | solute carrier family 27 (fatty acid transporter), member 2 | .0113 | 1.4809 | .0108 | 1.4876 |
| **159** | SLC43A2 | solute carrier family 43, member 2 | .0003 | 1.2375 | .0006 | 1.2089 |
| **160** | SLC44A5 | solute carrier family 44, member 5 | .0087 | -1.2367 | .0001 | -1.5998 |
| **161** | SLC7A11 | solute carrier family 7, member 11 | .0048 | 1.3111 | .0001 | 1.7716 |
| **162** | SLCO1C1 | solute carrier organic anion transporter family, member 1C1 | .0432 | -1.4285 | .0016 | -2.1357 |
| **163** | SLFN5 | schlafen family member 5 | .0000 | 2.9082 | .0000 | 6.5694 |
| **164** | SLN | sarcolipin | .0004 | -1.6016 | .0016 | -1.4407 |
| **165** | SMR3A | submaxillary gland androgen regulated protein 3A | .0036 | -1.5494 | .0060 | -1.4800 |
| **166** | SP100 | SP100 nuclear antigen | .0001 | 2.7683 | .0000 | 5.1035 |
| **167** | SP110 | SP110 nuclear body protein | .0000 | 1.9264 | .0000 | 2.8375 |
| **168** | SPATS2L | spermatogenesis associated, serine-rich 2-like | .0031 | 1.2076 | .0001 | 1.4599 |
| **169** | SST | somatostatin | .0288 | -1.2600 | .0093 | -1.3564 |
| **170** | ST8SIA3 | ST8 alpha-N-acetyl-neuraminide alpha-2,8-sialyltransferase 3 | .0390 | -1.2328 | .0056 | -1.3991 |
| **171** | STAT1 | signal transducer and activator of transcription 1, 91kDa | .0000 | 1.9603 | .0000 | 2.3445 |
| **172** | STAT2 | signal transducer and activator of transcription 2 | .0029 | 1.2293 | .0001 | 1.5424 |
| **173** | SULF2 | sulfatase 2 | .0017 | -1.2731 | .0005 | -1.3541 |
| **174** | TAP1 | transporter 1, ATP-binding cassette, sub-family B (MDR/TAP) | .0004 | 1.4556 | .0000 | 2.1199 |
| **175** | TAP2 | transporter 2, ATP-binding cassette, sub-family B (MDR/TAP) | .0000 | 1.3521 | .0001 | 1.3254 |
| **176** | TDO2 | tryptophan 2,3-dioxygenase | .0012 | -1.8207 | .0002 | -2.3823 |
| **177** | TDRD7 | tudor domain containing 7 | .0002 | 1.6277 | .0000 | 2.1877 |
| **178** | TESC | tescalcin | .0064 | -1.2778 | .0037 | -1.3176 |
| **179** | TLR3 | toll-like receptor 3 | .0000 | 1.4333 | .0000 | 1.7502 |
| **180** | TMEM229A | transmembrane protein 229A | .0143 | -1.3474 | .0105 | -1.3782 |
| **181** | TNFRSF10D | tumor necrosis factor receptor superfamily, member 10d | .0005 | -1.6536 | .0002 | -1.7999 |
| **182** | TRAJ23 | T cell receptor alpha joining 23 | .0005 | -1.6448 | .0158 | -1.2783 |
| **183** | TRANK1 | tetratricopeptide repeat and ankyrin repeat containing 1 | .0492 | 1.2089 | .0001 | 3.2876 |
| **184** | TRIM14 | tripartite motif containing 14 | .0019 | 1.5826 | .0004 | 1.8787 |
| **185** | TRIM21 | tripartite motif containing 21 | .0002 | 1.5350 | .0000 | 2.3863 |
| **186** | TRIM22 | tripartite motif containing 22 | .0001 | 1.6354 | .0000 | 1.9637 |
| **187** | TRIM25 | tripartite motif containing 25 | .0000 | 1.4487 | .0000 | 1.6746 |
| **188** | TRIM69 | tripartite motif containing 69 | .0161 | 1.2287 | .0097 | 1.2611 |
| **189** | UBA7 | ubiquitin-like modifier activating enzyme 7 | .0000 | 2.2169 | .0000 | 3.5884 |
| **190** | UBE2L6 | ubiquitin-conjugating enzyme E2L 6 | .0023 | 1.4078 | .0000 | 2.0867 |
| **191** | USP18 | ubiquitin specific peptidase 18 | .0000 | 2.7977 | .0000 | 4.2467 |
| **192** | WARS | tryptophanyl-tRNA synthetase | .0003 | 1.2103 | .0000 | 1.5337 |
| **193** | XAF1 | XIAP associated factor 1 | .0000 | 4.8040 | .0000 | 12.1451 |
| **194** | XKRX | XK, Kell blood group complex subunit-related, X-linked | .0072 | -1.3228 | .0006 | -1.5886 |
| **195** | YME1L1 | YME1-like 1 ATPase | .0046 | -1.3095 | .0000 | -1.2949 |
| **196** | ZC3HAV1 | zinc finger CCCH-type, antiviral 1 | .0022 | 1.2623 | .0013 | 1.2966 |
| **197** | ZKSCAN3 | zinc finger with KRAB and SCAN domains 3 | .0079 | -1.2372 | .0052 | -1.2633 |
| **198** | ZNF132 | zinc finger protein 132 | .0157 | 1.2313 | .0152 | 1.2336 |
| **199** | ZNF530 | zinc finger protein 530 | .0084 | -1.2804 | .0027 | -1.3688 |
| **200** | ZNFX1 | zinc finger, NFX1-type containing 1 | .0003 | 1.4190 | .0000 | 1.9431 |
