# Supplementary material for: Interferon-Alpha Reduces Human Hippocampal Neurogenesis and Increases Apoptosis via Activation of Distinct STAT1-Dependent Mechanisms
Source: Int J Neuropsychopharmacol. 2017 Oct 10;21(2):187–200. doi: 10.1093/ijnp/pyx083 (PMC5793815; doi:10.1093/ijnp/pyx083)
Supplement: Supplementary Table 4 [file pyx083_suppl_supplementary_table_4.docx]

**Supplementary Table 4. Validation of Genes Regulated by IFN-α 500 pg/mL Only and IFN-α 5000 pg/L Only When Compared with Vehicle**

| **Validation of genes regulated by IFN-α 500pg/ml only vs vehicle** | |  |  |  |  |
| --- | --- | --- | --- | --- | --- |
|  |  | **Microarray** | | **qRT-PCR** |  |
|  |  |  |  |  |  |
| **Gene** | **Gene name** | ***P*** | **Fold** | ***P*** | **Fold** |
| AKR7A3 | aldo-keto reductase family 7 member A3 | .02 | -1.7 | 9.003 | -1.8 |
| TFF3 | trefoil factor 3 | .02 | -1.25 | .002 | -1.35 |
| S100A13 | S100 calcium binding protein A13 | .03 | -1.2 | .01 | -1.3 |
| TMEM86A | transmembrane protein 86A | .04 | -1.2 | .02 | -1.4 |
| TPH1 | tryptophan hydroxylase 1 | .008 | -1.2 | .002 | -1.35 |
|  |  |  |  |  |  |
|  |  |  |  |  |  |
| **Validation of Genes Regulated by IFN-α 5000 pg/mL only vs vehicle** | |  |  |  |  |
|  |  | **Microarray** | | **qRT-PCR** |  |
|  |  |  |  |  |  |
| **Gene** | **Gene name** | ***P*** | **Fold** | ***P*** | **Fold** |
| AQP4 | aquaporin 4 | .0007 | -1.5 | .0004 | -1.7 |
| HDAC5 | histone deacetylase 5 | .005 | -1.2 | .0001 | -1.3 |
| PIDD1 | p53-induced death domain protein 1 | .02 | 1.2 | .001 | 1.4 |
| CASP1 | caspase 1 | .003 | 1.3 | .0001 | 1.5 |
| CCND1 | cyclin D1 | .002 | 1.3 | .0001 | 1.45 |
