# Supplementary material for: Interferon-Alpha Reduces Human Hippocampal Neurogenesis and Increases Apoptosis via Activation of Distinct STAT1-Dependent Mechanisms
Source: Int J Neuropsychopharmacol. 2017 Oct 10;21(2):187–200. doi: 10.1093/ijnp/pyx083 (PMC5793815; doi:10.1093/ijnp/pyx083)
Supplement: Supplementary Table 5 [file pyx083_suppl_supplementary_table_5.docx]

**Supplementary Table 5. Validation of Genes Commonly Regulated by IFN-α 500 pg/mL and IFN-α 5000 pg/mL When Compared with Vehicle**

| **Validation of genes regulated by both IFN-α 500 pg/mL and IFN-α 5000 pg/mL** | |  |  |  |  |
| --- | --- | --- | --- | --- | --- |
| **IFN-α 500pg/ml vs vehicle** | |  |  |  |  |
|  |  | **Microarray** | | **qRT-PCR** |  |
|  |  |  |  |  |  |
| **Gene** | **Gene name** | ***P*** | **Fold** | ***P*** | **Fold** |
| HERC5 | HECT and RLD domain containing E3 ubiquitin protein ligase 5 | <.0001 | 4.3000 | .0003 | 3.4000 |
| ISG15 | ISG15 ubiquitin-like modifier | <.0001 | 2.3000 | <.0001 | 2.1000 |
| STAT1 | signal transducer and activator of transcription 1 | <.0001 | 2.3000 | <.0001 | 2.7000 |
| USP18 | ubiquitin specific peptidase 18 | <.0001 | 2.8000 | <.0001 | 2.1000 |
| UBE2L6 | ubiquitin-conjugating enzyme E2L 6 | .0020 | 1.4000 | .0010 | 1.4000 |
| UBA7 | ubiquitin-like modifier activating enzyme 7 | <.0001 | 2.2000 | .0009 | 2.0000 |
|  |  |  |  |  |  |
|  |  |  |  |  |  |
| **IFN-α 5000 pg/ml vs vehicle** | |  |  |  |  |
|  |  | **Microarray** | | **qRT-PCR** |  |
|  |  |  |  |  |  |
| **Gene** | **Gene name** | ***P*** | **Fold** | ***P*** | **Fold** |
| HERC5 | HECT and RLD domain containing E3 ubiquitin protein ligase 5 | <.0001 | 9.6000 | .0023 | 7.0500 |
| ISG15 | ISG15 ubiquitin-like modifier | <.0001 | 3.5000 | <.0001 | 3.4000 |
| STAT1 | signal transducer and activator of transcription 1 | <.0001 | 2.3000 | <.0001 | 3.1000 |
| UBA7 | ubiquitin-like modifier activating enzyme 7 | <.0001 | 3.6000 | <.0001 | 3.9000 |
| UBE2L6 | ubiquitin-conjugating enzyme E2L 6 | <.0001 | 2.1000 | <.0001 | 2.1000 |
| USP18 | ubiquitin specific peptidase 18 | <.0001 | 4.2000 | <.0001 | 3.5000 |
