# Supplementary material for: Interferon-Alpha Reduces Human Hippocampal Neurogenesis and Increases Apoptosis via Activation of Distinct STAT1-Dependent Mechanisms
Source: Int J Neuropsychopharmacol. 2017 Oct 10;21(2):187–200. doi: 10.1093/ijnp/pyx083 (PMC5793815; doi:10.1093/ijnp/pyx083)
Supplement: Supplementary Table 6 [file pyx083_suppl_supplementary_table_6.docx]

**Supplementary Table 6.** **Pathways and Related Molecules Modulated by IFN-α in Human Progenitors**

| **Pathways modulated by IFN-**α **500 pg/mL** | | |
| --- | --- | --- |
| **Pathway name** | **Molecules** | |
| 4-aminobutyrate Degradation I | ALDH5A1 | |
| Adenosine Nucleotides Degradation II | NT5C1A | |
| Agranulocyte Adhesion and Diapedesis | SELL,MMP14,CCL25 | |
| Amyotrophic Lateral Sclerosis Signalling | CASP7 | |
| Apoptosis Signalling | CASP7 | |
| Aryl Hydrocarbon Receptor Signalling | ALDH5A1 | |
| Bladder Cancer Signalling | MMP14 | |
| cAMP-mediated signalling | LPAR1,PDE10A,CHRM4 | |
| Cardiac Î²-adrenergic Signalling | PDE10A | |
| Coagulation System | F7 | |
| Cytotoxic T Lymphocyte-mediated Apoptosis of Target Cells | CASP7 | |
| Death Receptor Signalling | CASP7 | |
| Eicosanoid Signalling | DPEP2 | |
| EIF2 Signalling | RPL26L1,RPL23 | |
| Endoplasmic Reticulum Stress Pathway | CASP7 | |
| Endothelin-1 Signalling | CASP7 | |
| eNOS Signalling | LPAR1 | |
| Extrinsic Prothrombin Activation Pathway | F7 | |
| GABA Receptor Signalling | ALDH5A1 | |
| Gap Junction Signalling | LPAR1 | |
| GÎ±12/13 Signalling | LPAR1 | |
| GÎ±i Signalling | LPAR1,CHRM4 | |
| Glutamate Degradation III (via 4-aminobutyrate) | ALDH5A1 | |
| G-Protein Coupled Receptor Signalling | LPAR1,PDE10A,CHRM4 | |
| Granulocyte Adhesion and Diapedesis | SELL,MMP14,CCL25 | |
| Guanosine Nucleotides Degradation III | NT5C1A | |
| Gustation Pathway | PDE10A | |
| Hepatic Fibrosis / Hepatic Stellate Cell Activation | CXCR3 | |
| HIF1Î± Signalling | MMP14 | |
| Histidine Degradation VI | CYP4F11 | |
| IL-15 Production | PTK6 | |
| Inhibition of Matrix Metalloproteases | MMP14 | |
| Leukotriene Biosynthesis | DPEP2 | |
| Methylglyoxal Degradation III | AKR7A3 | |
| NAD Salvage Pathway II | NT5C1A | |
| Natural Killer Cell Signalling | NCR1 | |
| Notch Signalling | HEY2 | |
| NRF2-mediated Oxidative Stress Response | AKR7A3 | |
| Pathogenesis of Multiple Sclerosis | CXCR3 | |
| PEDF Signalling | CASP7 | |
| Pregnenolone Biosynthesis | CYP4F11 | |
| Purine Nucleotides Degradation II (Aerobic) | NT5C1A | |
| Relaxin Signalling | PDE10A | |
| RhoA Signalling | LPAR1 | |
| Role of BRCA1 in DNA Damage Response | UIMC1 | |
| Role of Tissue Factor in Cancer | F7 | |
| Serotonin and Melatonin Biosynthesis | TPH1 | |
| Serotonin Receptor Signalling | TPH1 | |
| Sperm Motility | PTK6 | |
| Sphingosine-1-phosphate Signalling | CASP7 | |
| TNFR1 Signalling | CASP7 | |
| tRNA Splicing | PDE10A | |
| Tumoricidal Function of Hepatic Natural Killer Cells | CASP7 | |
| TWEAK Signalling | CASP7 | |
| Ubiquinol-10 Biosynthesis (Eukaryotic) | CYP4F11 | |
| Urate Biosynthesis/Inosine 5'-phosphate Degradation | NT5C1A | |
|  |  | |
| **Pathways modulated by IFN-**α **5000 pg/mL** | | |
| 1D-myo-inositol Hexakisphosphate Biosynthesis II (Mammalian) | ITPKB | |
| 2-oxobutanoate Degradation I | PCCA | |
| 3-phosphoinositide Biosynthesis | PPP1R1B,PTPN20,PPIP5K1,VAV1,IGBP1,DUSP16 | |
| 3-phosphoinositide Degradation | PPP1R1B,PTPN20,PPIP5K1,IGBP1,DUSP16 | |
| 4-1BB Signalling in T Lymphocytes | NFKBIA | |
| Actin Cytoskeleton Signalling | IQGAP2,TIAM1,FGF2,FGF11,VAV1,APC | |
| Acute Myeloid Leukemia Signalling | FLT3LG,CCND1 | |
| Acute Phase Response Signalling | C1R,SOCS1,SERPING1,C3,NFKBIA,ITIH4,C1S,CFB,C2 | |
| Adipogenesis pathway | PPARG,SMAD9,FGF2,PPIP5K1,NOCT,HDAC5 | |
| Agranulocyte Adhesion and Diapedesis | CXCL10,VCAM1,ICAM1,CLDN1,MMP16,CXCL12,MMP2,CD34,CX3CL1,MMP24 | |
| Airway Pathology in Chronic Obstructive Pulmonary Disease | MMP2 | |
| Amyloid Processing | MAPT,BACE2 | |
| Antigen Presentation Pathway | TAPBP | |
| April Mediated Signalling | NFKBIA | |
| Arginine Biosynthesis IV | GLUD1 | |
| Aryl Hydrocarbon Receptor Signalling | NR2F1,GSTM3,MDM2,CCND1 | |
| Asparagine Biosynthesis I | ASNS | |
| Atherosclerosis Signalling | COL1A2,VCAM1,ICAM1,CXCL12 | |
| ATM Signalling | NFKBIA,GADD45G,MDM2 | |
| B Cell Activating Factor Signalling | NFKBIA | |
| B Cell Receptor Signalling | NFKBIA,MAP3K11,PTK2B,PAG1,VAV1,FCGR2B | |
| Basal Cell Carcinoma Signalling | WNT8B,APC | |
| Bladder Cancer Signalling | FGF2,MMP16,FGF11,MDM2,MMP2,CCND1,MMP24 | |
| Calcium Transport I | ATP2B2 | |
| cAMP-mediated signalling | HTR6,PDE3A,S1PR1,DUSP4,AKAP3,PKIA,DRD4,FPR1 | |
| Cardiac Î²-adrenergic Signalling | PDE3A,AKAP3,PKIA | |
| CD27 Signalling in Lymphocytes | NFKBIA,MAP3K11 | |
| CD40 Signalling | ICAM1,NFKBIA,TNFAIP3 | |
| Cdc42 Signalling | IQGAP2,MAP3K11,LLGL1,VAV1,APC | |
| CDK5 Signalling | MAPK4,PPP1R1B,MAPT | |
| Cell Cycle Regulation by BTG Family Proteins | CCND1,NOCT | |
| Cell Cycle: G1/S Checkpoint Regulation | MDM2,CCND1,HDAC5 | |
| Chemokine Signalling | PTK2B,CXCL12 | |
| Chronic Myeloid Leukemia Signalling | MDM2,CCND1,HDAC5 | |
| Circadian Rhythm Signalling | BHLHE41,CRY1 | |
| Coagulation System | PLAUR | |
| Colorectal Cancer Metastasis Signalling | MMP16,MMP2,WNT8B,CCND1,APC,MMP24 | |
| Complement System | C1R,SERPING1,C3,C1S,CFB,C2 | |
| Crosstalk between Dendritic Cells and Natural Killer Cells | MICB,TNFSF10,MICA | |
| Cyclins and Cell Cycle Regulation | CCND1,HDAC5 | |
| Death Receptor Signalling | PARP6,NFKBIA,PARP10,TNFSF10 | |
| D-myo-inositol (1,3,4)-trisphosphate Biosynthesis | ITPKB | |
| D-myo-inositol (1,4,5,6)-Tetrakisphosphate Biosynthesis | PPP1R1B,PTPN20,PPIP5K1,IGBP1,DUSP16 | |
| D-myo-inositol (3,4,5,6)-tetrakisphosphate Biosynthesis | PPP1R1B,PTPN20,PPIP5K1,IGBP1,DUSP16 | |
| D-myo-inositol-5-phosphate Metabolism | PPP1R1B,PTPN20,PPIP5K1,IGBP1,DUSP16 | |
| DNA Methylation and Transcriptional Repression Signalling | CHD3 | |
| Dopamine Degradation | MAOB,SULT1C4,LRTOMT | |
| Dopamine Receptor Signalling | MAOB,PPP1R1B,DRD4 | |
| Dopamine-DARPP32 Feedback in cAMP Signalling | KCNJ2,PPP1R1B,KCNJ10,DRD4 | |
| Ephrin B Signalling | CXCL12,VAV1 | |
| Erythropoietin Signalling | SOCS1,NFKBIA | |
| Estrogen-mediated S-phase Entry | CCND1 | |
| FGF Signalling | MET,FGF2,FGF11 | |
| FXR/RXR Activation | PPARG,C3,ITIH4,VLDLR | |
| GABA Receptor Signalling | SLC6A11,GAD2,KCNQ2,SLC6A1 | |
| GADD45 Signalling | GADD45G,CCND1 | |
| GÎ±12/13 Signalling | NFKBIA,PTK2B,VAV1 | |
| GÎ±i Signalling | S1PR1,DRD4,FPR1 | |
| Glioblastoma Multiforme Signalling | MDM2,WNT8B,CCND1,APC | |
| Glioma Invasiveness Signalling | PLAUR,MMP2 | |
| Glioma Signalling | TGFA,MDM2,CCND1 | |
| Glutamate Biosynthesis II | GLUD1 | |
| Glutamate Degradation III (via 4-aminobutyrate) | GAD2 | |
| Glutamate Degradation X | GLUD1 | |
| Glutamate Dependent Acid Resistance | GAD2 | |
| Glutathione Redox Reactions I | GPX8 | |
| Glutathione-mediated Detoxification | GSTM3 | |
| Glycerol Degradation I | GPD1 | |
| Glycerol-3-phosphate Shuttle | GPD1 | |
| Glycine Degradation (Creatine Biosynthesis) | GATM | |
| Glycogen Biosynthesis II (from UDP-D-Glucose) | GYG2 | |
| G-Protein Coupled Receptor Signalling | HTR6,NFKBIA,PTK2B,PDE3A,S1PR1,DUSP4,DRD4,FPR1 | |
| Granulocyte Adhesion and Diapedesis | CXCL10,VCAM1,ICAM1,CLDN1,MMP16,CXCL12,MMP2,CX3CL1,MMP24,FPR1 | |
| Granzyme A Signalling | HIST1H1B,H1F0 | |
| Growth Hormone Signalling | SOCS1,IGFBP3 | |
| Gustation Pathway | TAS2R19,PDE3A,TAS2R46 | |
| Heparan Sulfate Biosynthesis | SULT1C4,EXTL2 | |
| Heparan Sulfate Biosynthesis (Late Stages) | SULT1C4,EXTL2 | |
| Hepatic Fibrosis / Hepatic Stellate Cell Activation | MET,COL1A2,VCAM1,ICAM1,EDNRB,EDN1,FGF2,TGFA,IGFBP3,MMP2 | |
| HER-2 Signalling in Breast Cancer | MDM2,MMP2,CCND1 | |
| HGF Signalling | MET,ELF4,MAP3K11,CCND1 | |
| HIF1Î± Signalling | EDN1,MAPK4,MMP16,MDM2,MMP2,MMP24 | |
| HIPPO signalling | LLGL1,AJUBA,CRB1 | |
| Histidine Degradation VI | MICAL1 | |
| Human Embryonic Stem Cell Pluripotency | FGF2,S1PR1,WNT8B,APC | |
| Hypoxia Signalling in the Cardiovascular System | NFKBIA,EDN1,MDM2 | |
| Î³-glutamyl Cycle | CHAC1 | |
| IL-10 Signalling | CCR1,NFKBIA,FCGR2B | |
| IL-15 Production | MAP3K11,PTK2B,IRF1 | |
| IL-17A Signalling in Fibroblasts | NFKBIA | |
| IL-17A Signalling in Gastric Cells | CXCL10 | |
| IL-2 Signalling | SOCS1,PTK2B | |
| IL-8 Signalling | VCAM1,ICAM1,PTK2B,MMP2,CCND1 | |
| Inflammasome pathway | CASP1 | |
| Inhibition of Matrix Metalloproteases | MMP16,MMP2,TFPI2,MMP24 | |
| iNOS Signalling | NFKBIA,IRF1 | |
| Inositol Pyrophosphates Biosynthesis | PPIP5K1 | |
| Interferon Signalling | SOCS1,IRF1 | |
| Intrinsic Prothrombin Activation Pathway | COL1A2 | |
| L-cysteine Degradation III | MPST | |
| L-DOPA Degradation | LRTOMT | |
| Leptin Signalling in Obesity | NPY,PDE3A | |
| Leukocyte Extravasation Signalling | VCAM1,ICAM1,PTK2B,CLDN1,MMP16,CXCL12,VAV1,MMP2,MMP24 | |
| Lymphotoxin Î² Receptor Signalling | VCAM1,NFKBIA | |
| Melanoma Signalling | MDM2,CCND1 | |
| Melatonin Degradation I | LARGE1,SULT1C4,LARGE2 | |
| Melatonin Degradation II | MAOB | |
| Methylmalonyl Pathway | PCCA | |
| MIF Regulation of Innate Immunity | NFKBIA | |
| MIF-mediated Glucocorticoid Regulation | NFKBIA | |
| NAD Biosynthesis III | NAMPT | |
| Neuroprotective Role of THOP1 in Alzheimer's Disease | MAPT | |
| Nicotine Degradation II | LARGE1,LARGE2 | |
| Nicotine Degradation III | LARGE1,LARGE2 | |
| nNOS Signalling in Skeletal Muscle Cells | CHRNA1 | |
| Non-Small Cell Lung Cancer Signalling | TGFA,CCND1 | |
| Noradrenaline and Adrenaline Degradation | MAOB,LRTOMT | |
| Notch Signalling | DLL3,HES7,HEY1 | |
| Oncostatin M Signalling | MT2A | |
| Ovarian Cancer Signalling | EDN1,MMP2,WNT8B,CCND1,APC | |
| p53 Signalling | GADD45G,MDM2,PIDD1,CCND1,SERPINE2 | |
| Pancreatic Adenocarcinoma Signalling | TGFA,MDM2,CCND1 | |
| Pathogenesis of Multiple Sclerosis | CXCL10,CCR1 | |
| PEDF Signalling | PPARG,NFKBIA | |
| Phenylalanine Degradation IV (Mammalian, via Side Chain) | MAOB | |
| PI3K Signalling in B Lymphocytes | C3,NFKBIA,CD180,VAV1,FCGR2B | |
| PI3K/AKT Signalling | NFKBIA,MDM2,CCND1 | |
| PKCÎ¸ Signalling in T Lymphocytes | NFKBIA,MAP3K11,VAV1 | |
| Polyamine Regulation in Colon Cancer | PPARG,PSME1,APC | |
| PPAR Signalling | PPARG,NR2F1,NFKBIA | |
| Pregnenolone Biosynthesis | MICAL1 | |
| Prolactin Signalling | SOCS1,IRF1 | |
| Prostanoid Biosynthesis | TBXAS1 | |
| Prostate Cancer Signalling | NFKBIA,MDM2,CCND1 | |
| Protein Kinase A Signalling | HIST1H1B,NFKBIA,PTK2B,PPP1R1B,PDE3A,DUSP4,AKAP3,H1F0,EBI3,DUSP16 | |
| Putrescine Degradation III | MAOB | |
| Rac Signalling | TIAM1,IQGAP2,MAP3K11,PTK2B | |
| RANK Signalling in Osteoclasts | NFKBIA,MAP3K11,PTK2B | |
| Reelin Signalling in Neurons | MAP3K11,MAPT,ARHGEF2,VLDLR,DCX | |
| Regulation of IL-2 Expression in Activated and Anergic T Lymphocytes | NFKBIA,VAV1 | |
| Regulation of the Epithelial-Mesenchymal Transition Pathway | MET,FGF2,SNAI1,FGF11,MMP2,WNT8B,APC | |
| Remodeling of Epithelial Adherens Junctions | MET,APC | |
| Renal Cell Carcinoma Signalling | MET,TGFA | |
| Retinoic acid Mediated Apoptosis Signalling | PARP6,PARP10,TNFSF10,IRF1 | |
| Role of Hypercytokinemia/hyperchemokinemia in the Pathogenesis of Influenza | CXCL10,CCR1 | |
| Role of IL-17F in Allergic Inflammatory Airway Diseases | CXCL10 | |
| Role of JAK family kinases in IL-6-type Cytokine Signalling | SOCS1 | |
| Role of JAK1 and JAK3 in Î³c Cytokine Signalling | SOCS1,PTK2B | |
| Role of JAK1, JAK2 and TYK2 in Interferon Signalling | SOCS1 | |
| Role of JAK2 in Hormone-like Cytokine Signalling | SOCS1 | |
| Role of Macrophages, Fibroblasts and Endothelial Cells in Rheumatoid Arthritis | SOCS1,VCAM1,ICAM1,NFKBIA,FRZB,FGF2,PRSS2,CXCL12,WNT8B,CCND1,APC | |
| Role of NANOG in Mammalian Embryonic Stem Cell Pluripotency | SMAD9,WNT8B,APC | |
| Role of Osteoblasts, Osteoclasts and Chondrocytes in Rheumatoid Arthritis | NFKBIA,FRZB,PTK2B,SMAD9,DLX5,WNT8B,APC | |
| Role of p14/p19ARF in Tumor Suppression | MDM2 | |
| Role of PKR in Interferon Induction and Antiviral Response | NFKBIA,IRF1 | |
| Role of RIG1-like Receptors in Antiviral Innate Immunity | NFKBIA | |
| Role of Wnt/GSK-3Î² Signalling in the Pathogenesis of Influenza | WNT8B,APC | |
| Serotonin Degradation | LARGE1,MAOB,SULT1C4,LARGE2 | |
| Serotonin Receptor Signalling | HTR6,MAOB | |
| Small Cell Lung Cancer Signalling | NFKBIA,CCND1 | |
| Sphingosine-1-phosphate Signalling | PTK2B,CASP1,S1PR1 | |
| STAT3 Pathway | SOCS1,MAP3K11 | |
| Superpathway of D-myo-inositol (1,4,5)-trisphosphate Metabolism | ITPKB | |
| Superpathway of Inositol Phosphate Compounds | ITPKB,PPP1R1B,PTPN20,PPIP5K1,VAV1,IGBP1,DUSP16 | |
| Superpathway of Melatonin Degradation | LARGE1,MAOB,SULT1C4,LARGE2 | |
| Superpathway of Methionine Degradation | PCCA | |
| T Cell Receptor Signalling | NFKBIA,PAG1,VAV1 | |
| TCA Cycle II (Eukaryotic) | OGDHL | |
| TGF-Î² Signalling | INHA,SMAD9 | |
| The Visual Cycle | RLBP1 | |
| Thiosulfate Disproportionation III (Rhodanese) | MPST | |
| Thyroid Cancer Signalling | PPARG,CXCL10,CCND1 | |
| Thyroid Hormone Metabolism II (via Conjugation and/or Degradation) | LARGE1,SULT1C4,LARGE2 | |
| Tight Junction Signalling | TIAM1,CLDN1,LLGL1,ARHGEF2,SNAP25 | |
| TNFR1 Signalling | NFKBIA,TNFAIP3 | |
| TNFR2 Signalling | NFKBIA,TNFAIP3 | |
| Toll-like Receptor Signalling | NFKBIA,TNFAIP3 | |
| TREM1 Signalling | ICAM1,CASP1,FCGR2B | |
| tRNA Charging | GARS,MARS | |
| tRNA Splicing | PDE3A | |
| Tryptophan Degradation X (Mammalian, via Tryptamine) | MAOB | |
| Tumoricidal Function of Hepatic Natural Killer Cells | ICAM1 | |
| TWEAK Signalling | NFKBIA | |
| Type I Diabetes Mellitus Signalling | SOCS1,GAD2,NFKBIA,IRF1 | |
| Type II Diabetes Mellitus Signalling | PPARG,SOCS1,NFKBIA | |
| Ubiquinol-10 Biosynthesis (Eukaryotic) | MICAL1 | |
| VDR/RXR Activation | CXCL10,IGFBP3 | |
| Wnt/Î²-catenin Signalling | SOX4,FRZB,MDM2,WNT8B,CCND1,APC | |
|  | | |
| **Pathways modulated by IFN-**α **500 pg/mL and IFN-**α **5000 pg/mL** | | |
| Activation of IRF by Cytosolic Pattern Recognition Receptors | | DHX58,IFIH1,IRF7,DDX58,STAT2,IRF9,STAT1,ADAR,IFIT2,ISG15 |
| Acute Myeloid Leukemia Signalling | | PML |
| Adenosine Nucleotides Degradation II | | NT5E |
| Agrin Interactions at Neuromuscular Junction | | AGRN |
| Allograft Rejection Signalling | | HLA-G,B2M,HLA-A,HLA-C,HLA-B,HLA-DQA1,HLA-F,HLA-E |
| Altered T Cell and B Cell Signalling in Rheumatoid Arthritis | | HLA-A,HLA-B,HLA-DQA1,TLR3 |
| Amyloid Processing | | CAPN6 |
| Amyotrophic Lateral Sclerosis Signalling | | CAPN6 |
| Androgen Biosynthesis | | HSD17B14 |
| Androgen Signalling | | GNG3 |
| Antigen Presentation Pathway | | HLA-G,B2M,PSMB9,NLRC5,HLA-A,HLA-C,HLA-B,HLA-DQA1,PSMB8,HLA-F,TAP1,TAP2,HLA-E |
| Antioxidant Action of Vitamin C | | PLA2G2F,RARRES3,PLCH2 |
| Antiproliferative Role of Somatostatin Receptor 2 | | SST,GNG3 |
| Apoptosis Signalling | | CAPN6 |
| Atherosclerosis Signalling | | PLA2G2F,RARRES3,ALOX5 |
| Autoimmune Thyroid Disease Signalling | | HLA-G,HLA-A,HLA-C,HLA-B,HLA-DQA1,HLA-F,HLA-E |
| B Cell Development | | HLA-A,HLA-B,HLA-DQA1 |
| Calcium-induced T Lymphocyte Apoptosis | | HLA-A,HLA-B,HLA-DQA1 |
| Cardiac Î²-adrenergic Signalling | | PLN,GNG3 |
| Caveolar-mediated Endocytosis Signalling | | B2M,HLA-A,HLA-C,HLA-B |
| CCR3 Signalling in Eosinophils | | PLA2G2F,GNG3 |
| CCR5 Signalling in Macrophages | | GNG3 |
| CD28 Signalling in T Helper Cells | | HLA-A,HLA-B,HLA-DQA1 |
| Cdc42 Signalling | | HLA-G,B2M,HLA-A,HLA-C,HLA-B,HLA-DQA1,HLA-F,HLA-E |
| CDP-diacylglycerol Biosynthesis I | | CDS1 |
| Cholecystokinin/Gastrin-mediated Signalling | | SST |
| CNTF Signalling | | STAT1 |
| Colorectal Cancer Metastasis Signalling | | GNG3,TLR3,STAT1 |
| Communication between Innate and Adaptive Immune Cells | | HLA-G,B2M,HLA-A,HLA-C,HLA-B,TLR3,HLA-F,HLA-E |
| Corticotropin Releasing Hormone Signalling | | CNR1 |
| CREB Signalling in Neurons | | GNG3,PLCH2 |
| Crosstalk between Dendritic Cells and Natural Killer Cells | | HLA-G,HLA-A,HLA-C,HLA-B,TLR3,HLA-F,HLA-E |
| CTLA4 Signalling in Cytotoxic T Lymphocytes | | B2M,HLA-A,HLA-C,HLA-B |
| Cytotoxic T Lymphocyte-mediated Apoptosis of Target Cells | | B2M,HLA-A,HLA-C,HLA-B |
| Death Receptor Signalling | | ZC3HAV1,PARP12,PARP9,PARP14 |
| Dendritic Cell Maturation | | B2M,HLA-A,HLA-C,HLA-B,HLA-DQA1,STAT2,TLR3,PLCH2,STAT1 |
| D-myo-inositol (1,4,5)-Trisphosphate Biosynthesis | | PLCH2 |
| D-myo-inositol-5-phosphate Metabolism | | PPM1K,PLCH2 |
| EGF Signalling | | STAT1 |
| Eicosanoid Signalling | | PLA2G2F,RARRES3,ALOX5 |
| EIF2 Signalling | | RPS3A,EIF2AK2 |
| Endothelin-1 Signalling | | PLA2G2F,RARRES3,PLCH2 |
| Ephrin B Signalling | | GNG3 |
| ERK/MAPK Signalling | | PLA2G2F,STAT1 |
| ERK5 Signalling | | SH2D2A |
| Estrogen Biosynthesis | | HSD17B14 |
| Estrogen-Dependent Breast Cancer Signalling | | HSD17B14 |
| FAK Signalling | | CAPN6 |
| Fatty Acid Activation | | SLC27A2,ACSL5 |
| Fatty Acid Î²-oxidation I | | SLC27A2,ACSL5 |
| FLT3 Signalling in Hematopoietic Progenitor Cells | | STAT2,STAT1 |
| G Beta Gamma Signalling | | GNG3 |
| G Protein Signalling Mediated by Tubby | | GNG3 |
| GÎ±i Signalling | | CNR1,GNG3 |
| GÎ±s Signalling | | CNR1,GNG3 |
| Gluconeogenesis I | | PGAM2 |
| Glycolysis I | | PGAM2 |
| GM-CSF Signalling | | STAT1 |
| GPCR-Mediated Integration of Enteroendocrine Signalling Exemplified by an L Cell | | SST,PLCH2 |
| GPCR-Mediated Nutrient Sensing in Enteroendocrine Cells | | GNG3,PLCH2 |
| Graft-versus-Host Disease Signalling | | HLA-G,HLA-A,HLA-C,HLA-B,HLA-DQA1,HLA-F,HLA-E |
| Growth Hormone Signalling | | STAT1 |
| Guanosine Nucleotides Degradation III | | NT5E |
| Hepatic Fibrosis / Hepatic Stellate Cell Activation | | COL8A1,IGFBP5,STAT1 |
| Huntington's Disease Signalling | | CAPN6,PSME2,GNG3 |
| Hypoxia Signalling in the Cardiovascular System | | UBE2L6 |
| Î±-Adrenergic Signalling | | GNG3 |
| Î³-linolenate Biosynthesis II (Animals) | | SLC27A2,ACSL5 |
| iCOS-iCOSL Signalling in T Helper Cells | | HLA-A,HLA-B,HLA-DQA1 |
| IGF-1 Signalling | | IGFBP5 |
| IL-1 Signalling | | GNG3 |
| IL-15 Production | | STAT1 |
| IL-22 Signalling | | STAT1 |
| IL-3 Signalling | | STAT1 |
| IL-4 Signalling | | HLA-A,HLA-B,HLA-DQA1 |
| IL-9 Signalling | | STAT1 |
| iNOS Signalling | | STAT1 |
| Interferon Signalling | | IFIT3,OAS1,MX1,IFI35,IRF9,PSMB8,IFITM2,TAP1,ISG15,IFITM3,IFIT1,IFI6,STAT2,STAT1,IFITM1 |
| JAK/Stat Signalling | | STAT2,STAT1 |
| Leptin Signalling in Obesity | | PLCH2 |
| Leukotriene Biosynthesis | | ALOX5 |
| Lipid Antigen Presentation by CD1 | | B2M |
| LPS/IL-1 Mediated Inhibition of RXR Function | | SLC27A2,ACSL5 |
| Melatonin Signalling | | PLCH2 |
| MIF Regulation of Innate Immunity | | PLA2G2F |
| MIF-mediated Glucocorticoid Regulation | | PLA2G2F |
| Mitochondrial L-carnitine Shuttle Pathway | | SLC27A2,ACSL5 |
| NAD biosynthesis II (from tryptophan) | | TDO2 |
| NAD Salvage Pathway II | | NT5E |
| Neuroprotective Role of THOP1 in Alzheimer's Disease | | HLA-G,HLA-A,HLA-C,HLA-B,SST,HLA-F,HLA-E |
| NF-ÎºB Activation by Viruses | | EIF2AK2 |
| NF-ÎºB Signalling | | EIF2AK2,TLR3 |
| Nitric Oxide Signalling in the Cardiovascular System | | PLN |
| nNOS Signalling in Neurons | | CAPN6 |
| Nur77 Signalling in T Lymphocytes | | HLA-A,HLA-B,HLA-DQA1 |
| Oncostatin M Signalling | | STAT1 |
| OX40 Signalling Pathway | | HLA-G,B2M,HLA-A,HLA-C,HLA-B,HLA-DQA1,HLA-F,HLA-E |
| P2Y Purigenic Receptor Signalling Pathway | | GNG3,PLCH2 |
| p38 MAPK Signalling | | PLA2G2F,STAT1 |
| p53 Signalling | | PML |
| PDGF Signalling | | EIF2AK2,STAT1 |
| phagosome formation | | TLR3,PLCH2 |
| phagosome maturation | | B2M,HLA-A,HLA-C,HLA-B,TAP1 |
| Phenylalanine Degradation IV (Mammalian, via Side Chain) | | SLC27A2 |
| Phosphatidylglycerol Biosynthesis II (Non-plastidic) | | CDS1 |
| Phospholipases | | PLA2G2F,RARRES3,PLCH2 |
| PKCÎ¸ Signalling in T Lymphocytes | | HLA-A,HLA-B,HLA-DQA1 |
| Polyamine Regulation in Colon Cancer | | PSME2 |
| PPARÎ±/RXRÎ± Activation | | HELZ2,PLCH2 |
| Primary Immunodeficiency Signalling | | TAP1,TAP2 |
| Prolactin Signalling | | NMI,STAT1 |
| Protein Kinase A Signalling | | PLN,CDC14B,GNG3,PLCH2 |
| Protein Ubiquitination Pathway | | B2M,PSMB9,USP18,HLA-A,HLA-C,HLA-B,PSME2,PSMB8,TAP1,UBE2L6,TAP2 |
| Purine Nucleotides Degradation II (Aerobic) | | NT5E |
| Pyridoxal 5'-phosphate Salvage Pathway | | EIF2AK2 |
| Pyrimidine Deoxyribonucleotides De Novo Biosynthesis I | | CMPK2 |
| Pyrimidine Ribonucleotides De Novo Biosynthesis | | CMPK2 |
| Pyrimidine Ribonucleotides Interconversion | | CMPK2 |
| Rapoport-Luebering Glycolytic Shunt | | PGAM2 |
| RAR Activation | | PML,RARG |
| Reelin Signalling in Neurons | | CNR1 |
| Regulation of Cellular Mechanics by Calpain Protease | | CAPN6 |
| Retinoic acid Mediated Apoptosis Signalling | | ZC3HAV1,TNFRSF10D,PARP12,RARG,PARP9,PARP14 |
| Role of BRCA1 in DNA Damage Response | | FAAP24,STAT1 |
| Role of JAK family kinases in IL-6-type Cytokine Signalling | | STAT1 |
| Role of JAK1 and JAK3 in Î³c Cytokine Signalling | | STAT1 |
| Role of JAK1, JAK2 and TYK2 in Interferon Signalling | | STAT2,STAT1 |
| Role of JAK2 in Hormone-like Cytokine Signalling | | STAT1 |
| Role of Lipids/Lipid Rafts in the Pathogenesis of Influenza | | RSAD2 |
| Role of MAPK Signalling in the Pathogenesis of Influenza | | PLA2G2F,RARRES3 |
| Role of NFAT in Cardiac Hypertrophy | | GNG3,PLCH2 |
| Role of NFAT in Regulation of the Immune Response | | HLA-A,HLA-B,HLA-DQA1,GNG3 |
| Role of Oct4 in Mammalian Embryonic Stem Cell Pluripotency | | TDRD7 |
| Role of Pattern Recognition Receptors in Recognition of Bacteria and Viruses | | IFIH1,OAS1,IRF7,OAS2,DDX58,EIF2AK2,TLR3,OAS3 |
| Role of PKR in Interferon Induction and Antiviral Response | | EIF2AK2,TLR3,STAT1 |
| Role of RIG1-like Receptors in Antiviral Innate Immunity | | DHX58,IFIH1,IRF7,DDX58,TRIM25 |
| Salvage Pathways of Pyrimidine Ribonucleotides | | CMPK2,EIF2AK2 |
| SAPK/JNK Signalling | | SH2D2A |
| Semaphorin Signalling in Neurons | | CRMP1 |
| Sperm Motility | | PLA2G2F,RARRES3,PLCH2 |
| Stearate Biosynthesis I (Animals) | | SLC27A2,ACSL5 |
| Superpathway of Inositol Phosphate Compounds | | PPM1K,PLCH2 |
| Synaptic Long Term Depression | | PLA2G2F,RARRES3,PLCH2 |
| Systemic Lupus Erythematosus Signalling | | HLA-G,HLA-A,HLA-C,HLA-B,HLA-F,HLA-E |
| T Helper Cell Differentiation | | HLA-A,HLA-B,HLA-DQA1,STAT1 |
| TCA Cycle II (Eukaryotic) | | ACO1 |
| Tec Kinase Signalling | | STAT2,GNG3,STAT1 |
| TGF-Î² Signalling | | IRF7 |
| Thrombin Signalling | | GNG3,PLCH2 |
| Thrombopoietin Signalling | | STAT1 |
| Toll-like Receptor Signalling | | EIF2AK2,TLR3 |
| TREM1 Signalling | | NLRC5,TLR3 |
| Triacylglycerol Degradation | | DAGLA |
| tRNA Charging | | WARS |
| Tryptophan Degradation III (Eukaryotic) | | TDO2 |
| Tryptophan Degradation to 2-amino-3-carboxymuconate Semialdehyde | | TDO2 |
| Type I Diabetes Mellitus Signalling | | HLA-G,HLA-A,HLA-C,HLA-B,HLA-DQA1,HLA-F,STAT1,HLA-E |
| Type II Diabetes Mellitus Signalling | | SLC27A2,ACSL5 |
| Urate Biosynthesis/Inosine 5'-phosphate Degradation | | NT5E |
| UVA-Induced MAPK Signalling | | ZC3HAV1,PARP12,PLCH2,STAT1,PARP9,PARP14 |
| VDR/RXR Activation | | IGFBP5 |
| VEGF Family Ligand-Receptor Interactions | | PLA2G2F |
| VEGF Signalling | | SH2D2A |
| Virus Entry via Endocytic Pathways | | B2M,HLA-A,HLA-C,HLA-B |
| Wnt/Ca+ pathway | | PLCH2 |
