# Supplementary figures and images for: Interferon-Alpha Reduces Human Hippocampal Neurogenesis and Increases Apoptosis via Activation of Distinct STAT1-Dependent Mechanisms
Source: Int J Neuropsychopharmacol. 2017 Oct 10;21(2):187–200. doi: 10.1093/ijnp/pyx083 (PMC5793815; doi:10.1093/ijnp/pyx083)

**Supplementary Figure 1**


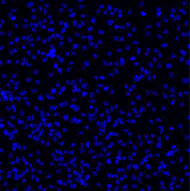

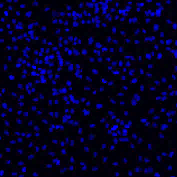


**DAPI**


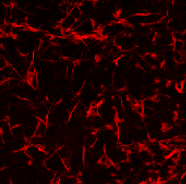

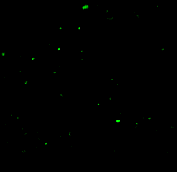

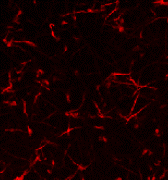


**a) Control**

**b) IFN-α 500pg/ml**

**MAP2**

**c) IFN-α 5000pg/ml**

**CC3**


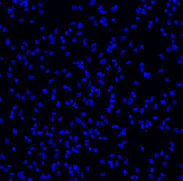

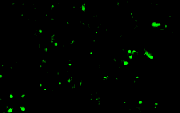

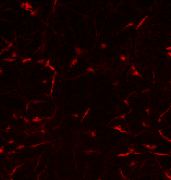

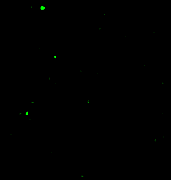

Supplement: Supplementary Figure 1 [file pyx083_suppl_supplementary_figure_1.docx]

**Supplementary Figure 2**

a)


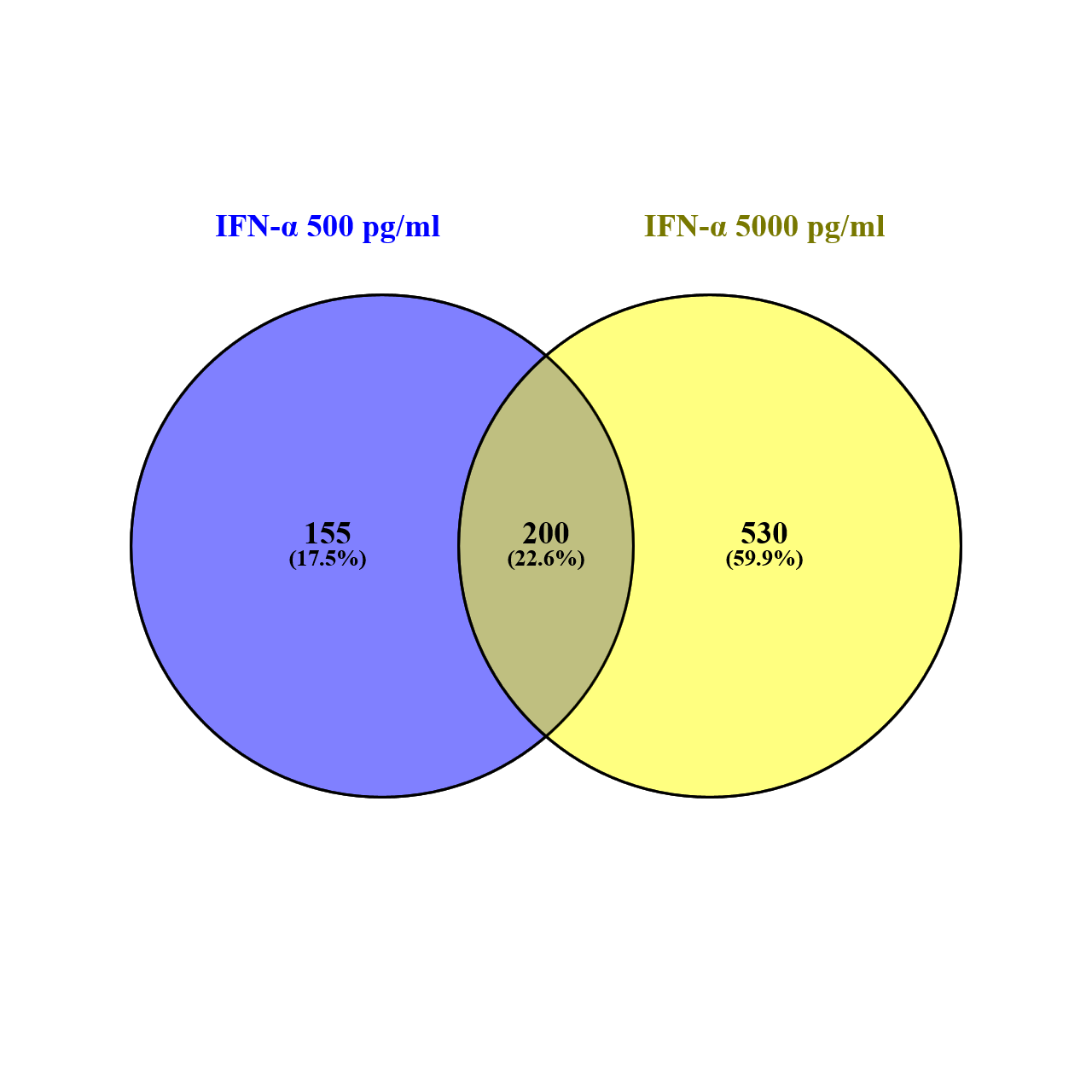

Supplement: Supplementary Figure 2 [file pyx083_suppl_supplementary_figure_2.doc]

**Supplementary Figure 3**

a)


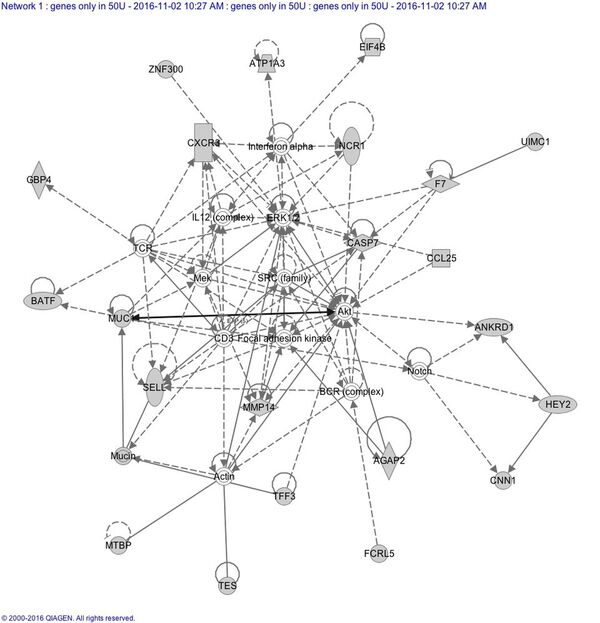


b)


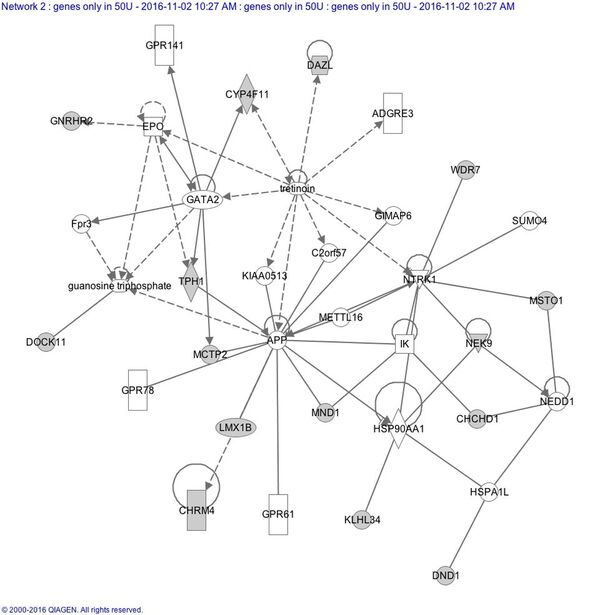


c)


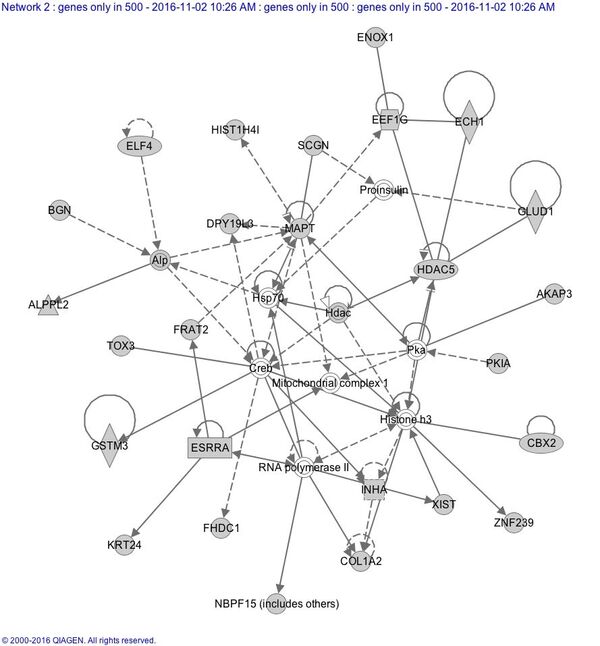


d)


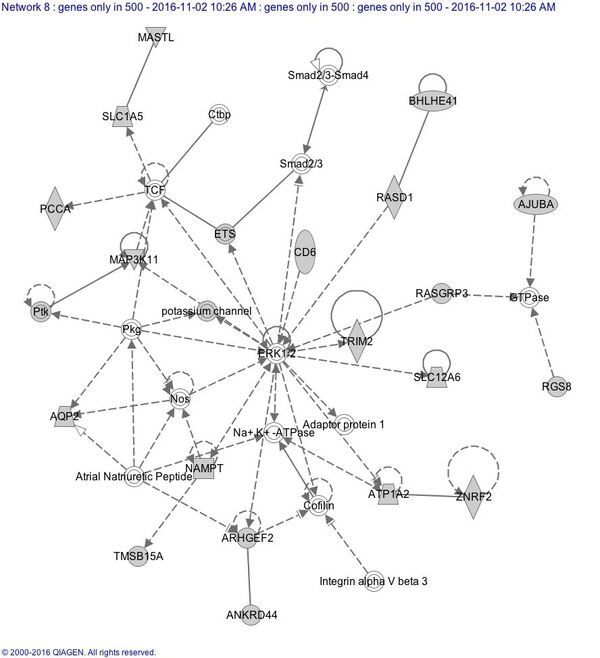


e)


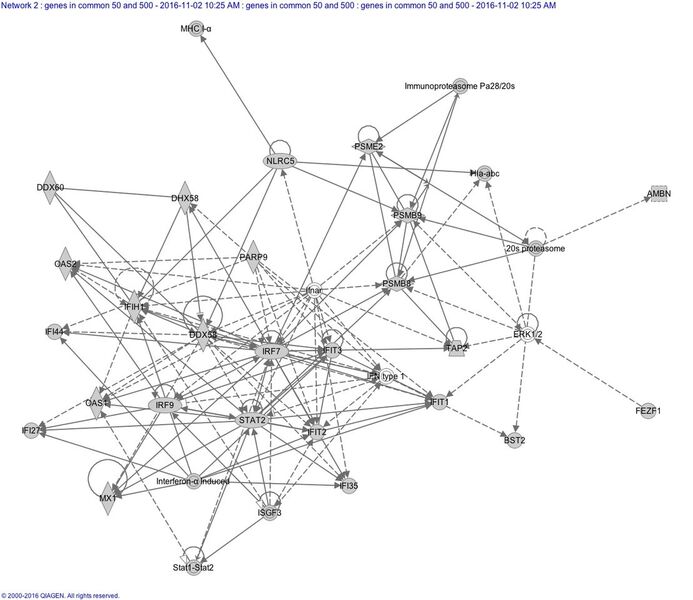


f)


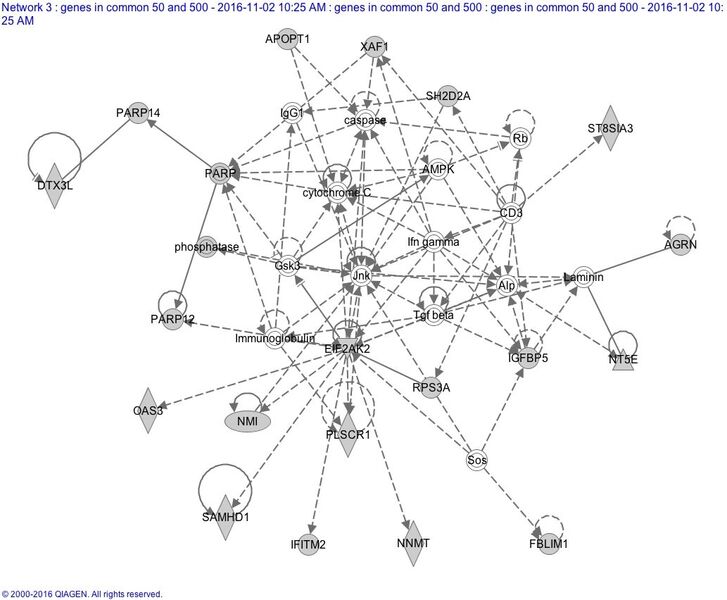

Supplement: Supplementary Figure 3 [file pyx083_suppl_supplementary_figure_3.doc]

**Supplementary Figure 4**


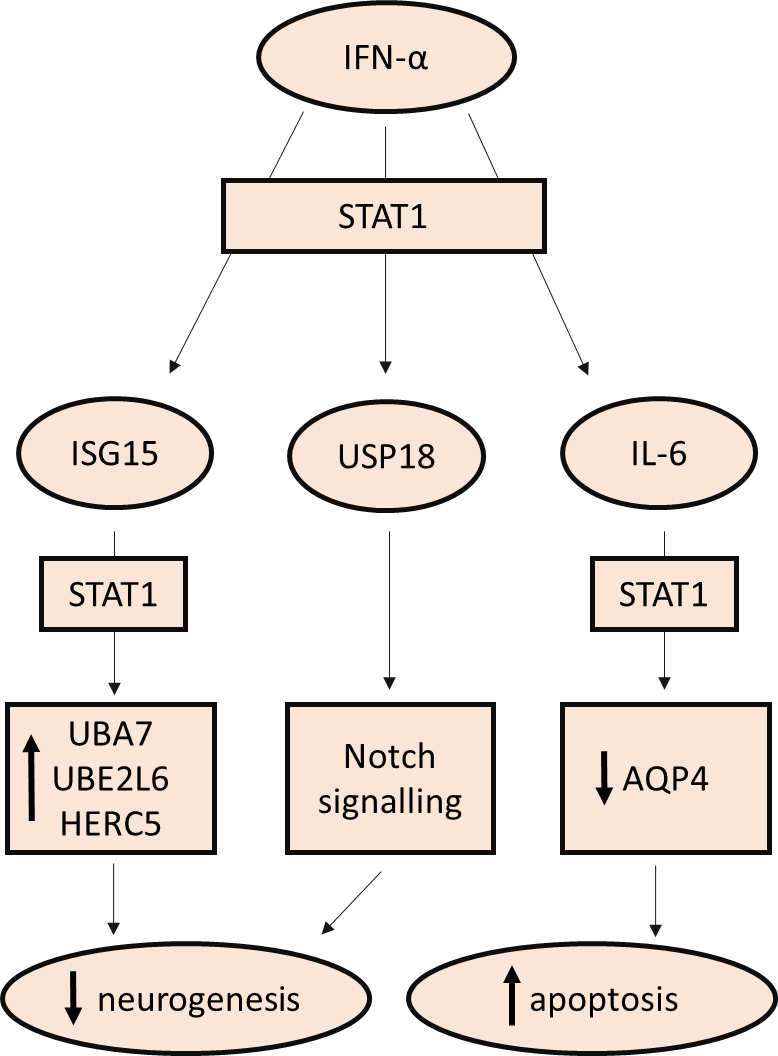

Supplement: Supplementary Figure 4 [file pyx083_suppl_supplementary_figure_4.doc]
