# Supplementary material for: Interferon-Alpha Reduces Human Hippocampal Neurogenesis and Increases Apoptosis via Activation of Distinct STAT1-Dependent Mechanisms
Source: Int J Neuropsychopharmacol. 2017 Oct 10;21(2):187–200. doi: 10.1093/ijnp/pyx083 (PMC5793815; doi:10.1093/ijnp/pyx083)
Supplement: Supplementary_Materials [file pyx083_suppl_supplementary_materials.docx]

**SUPPLEMENTARY METHODS**

**Cell Culture and Drug Treatments**

Multipotent human hippocampal progenitor cells (HHPCs) were used in all our experiments and were treated for 3 days of proliferation followed by 7 subsequent days of differentiation, with distinct proteins or drugs. We used 2 concentrations of IFN-α-2α: 500 and 5000 pg/mL, similar to the lowest (Francois et al., 2010) and highest (Bruno et al., 2004) mean levels of IFN-α found in the serum of patients treated with IFN-α; a concentration >5000 pg/mL caused significant cell death in our HHPCs (data not shown). These cells express IFN-α receptor (IFNAR) mRNA (data not shown). ISG15 and USP18 proteins were used at 10 pg/μL after a pilot study where we tested 1, 10, and 100 pg/μL. Fludarabine (STAT1 inhibitor) was used at 10 nM, again after a pilot study where we tested a range of concentrations from 10 nM up to 100 μM. IL-6 was used at 5 pg/mL based on results obtained from the cytokines analysis following the high dose of IFN-α in vitro (see Results). The concentration of 0.10 μg/mL for IL-6 antibody was selected on previous evidence of its efficacy in targeting and blocking IL-6 (Pathak et al., 2015).

**Drugs**

All reagents were purchased from Sigma-Aldrich unless otherwise stated. IL-6, EGF, and bFGF were purchased from Peprotech, and IL-6 antibody was purchased by R&D System Inc.

**Immunocytochemistry**

Neuronal differentiation was assessed with DCX and MAP2 after 10 days of treatment (3 days of proliferation followed by 7 subsequent days of differentiation) with: IFN-α (either alone or with fludarabine/IL-6 antibody); a cotreatment with ISG15, USP18, and IL-6 (either alone or with fludarabine); and SG15, or USP18 or IL-6 alone (either alone or with fludarabine). Cells were fixed and incubated in blocking solution (0.3% Triton X-100 and 5% normal donkey serum) in PBS for 1 hour at RT and then with primary antibodies (rabbit anti-DCX, 1:500; mouse anti-MAP2 [HM], 1:500, Abcam) at 4°C overnight. Subsequently, cells were incubated in blocking solution for 30 minutes, secondary antibodies (Alexa 488 donkey anti-rabbit; 1:1000; Alexa donkey 555 anti-mouse, 1:1000, Invitrogen) for 2 hours, and DAPI 33342 dye (0.02 mg/mL, Sigma-Aldrich) for 5 minutes at RT. Apoptotic cells were examined using CC3 primary (rabbit anti-CC3; 1:500; Technol) and secondary antibody (Alexa 488 donkey anti-rabbit; 1:1000; Invitrogen). Negative controls were incubated with unspecific rabbit IgGs (1:500, control for DCX), mouse IgGs (1:500, control for MAP2), and rabbit IgGs (1:500, control for CC3) in place of the specific primary antibody. The number of DCX, MAP2, CC3, and CC3/MAP2 positive cells over total DAPI positive cells was counted with CellInsight (see below). Three independent experiments were conducted on 3 independent cultures, and each sample was tested in triplicate.

**Automated Quantification of Immunofluorescence**

An automated approach using CellInsight NXT High Content Screening Platform (ThermoScientific) was employed to quantify cell number, markers of differentiation, and cell death. The iDEV workflow facility within the Cell Insight scanning software is characterized by the following steps: acquire images; primary object identification channel 1 (DAPI); primary target selection channel 2 (CC3 and DCX); and primary target selection channel 3 (MAP2). Once focused on nuclear staining (DAPI) to determine fields of interest, next exposures were adjusted in channels 2 and 3 to obtain the optimum exposure times for all wells and conditions across the 96-well plate. Image saturation was set within the 20% to 30% range to avoid under- or overexposure. Following this, images at 10x magnification were acquired that represent positive and negative staining. Based on values from the negative staining controls and good positive staining, distinct thresholds were set for average intensity of target regions of interest (nuclear or cell body) to delineate positive populations in both channels 2 and 3. These settings were applied to each well upon scanning of the plate and to all corresponding plates within an experiment, ensuring reproducibility and unbiased comparisons.

**Multiplex Cytokine Measurement**

Cell supernatants of differentiated cells were run on the Human ProInflammatory Multileplex Very-Sensitive Kit from Meso Scale Discovery using to the manufacturers’ instructions. Briefly, 50 µL of prepared samples was added into each well of the MSD plate, which was subsequently incubated for 2 hours with vigorous shaking at 1000 rpm at room temperature. The plate was then washed 3 times with 150 µL/well of Wash Buffer, and 25 µL of detection antibody solution was added to each well followed by another 2 hours incubation with vigorous shaking at 1000 rpm at room temperature. Finally, the plate was washed 3 times with 150 µL/well of Wash Buffer, and 150 µL of 2x Read Buffer T was added to each well. The plate was analyzed in the SECTOR Imager machine for the measurement of IL-6, IL-8, IL-10, IL-13, and IFN-γ.

**RNA Isolation and cDNA Synthesis**

Cells at 60% to 70% confluence in 6-well plates were treated for 3 days under proliferating conditions following by 7 days under differentiating condition with: IFN-α (either alone or with fludarabine/IL-6); a cotreatment with ISG15, USP18, and IL-6 (either alone or with fludarabine); and SG15, or USP18 or IL-6 alone (either alone or with fludarabine). RNA was isolated using the RNeasy Micro Kit (Qiagen) following the manufacturer’s instructions, and samples were kept frozen at -80°C until further use. RNA quantity and quality were assessed by evaluation of the A260/280 and A260/230 ratios using a Nanodrop spectrometer (NanoDrop Technologies). SuperScript III Reverse Transcriptase (Life Technologies) was then used to synthesise cDNA. Briefly, a mix of 1 μg RNA, 250 ng random hexamers (Life Technologies), and 1 mM dNTP mix (Thermo Scientific) was used to made up a 13-μL solution with nuclease-free H_2_O (Sigma-Aldrich). The mix was incubated for 5 minutes at 65°C on a heated block to denature RNA secondary structure. Subsequently, it was placed on ice and incubated for 1 minute to allow the annealing process. A volume of 7 μL consisting of 1x First Strand Buffer (Invitrogen), 5 mM dithiothreitol (Life Technologies), 40 units RNaseOUT (Life Technologies), 200 units SuperScript III Reverse Transcriptase (Life Technologies), and 3 μL of nuclease-free H_2_O (Sigma-Aldrich) was made and added to the samples. Samples now containing a total volume of 20 μL were incubated at 25ºC for 5 minutes, 50ºC for 1 hour, 55ºC for 30 minutes, and finally 70 ºC for 15 minutes to terminate the reaction. Samples were diluted to a concentration of 1:10 in nuclease-free H_2_O for subsequent qPCR analysis.

**Microarray Analysis**

Microarray assays were performed on RNA extracted from differentiated cells following protocol in the Affymetrix GeneChip Expression Analysis technical manual (Affymetrix), as previously described (Anacker et al., 2013). Briefly, 250 ng RNA was used to synthesize cDNA with the Ambion WT Expression Kit (ThermoFisher Scientific), which was then purified, fragmented, labelled, and hybridized onto Human Gene 1.1 ST Array Strips (Affymetrix). The reactions of hybridation, fluidics, and imaging were performed on the Affymetrix GeneAtlas platform (Affymetrix) instrument according to the manufacturer’s protocol. Affymetrix CEL files were imported into Partek Genomics Suite version 6.6 for data visualization and statistical testing. Quality control assessment was performed using Partek Genomic Suite 6.6. All samples passed the criteria for hybridization controls, labelling controls, and 3’/5’ Metrics. Background correction was conducted using Robust Multi-strip Average (Irizarry et al., 2003) to remove noise from auto fluorescence. After background correction, normalization was conducted using quantiles normalization (Bolstad et al., 2003) to normalize the distribution of probe intensities among different microarray chips. Subsequently, a summarization step was conducted using a linear median polish algorithm to integrate probe intensities in order to compute the expression levels for each gene transcript. Upon data upload, preprocessing of CEL data for the complete data set (total of 18 samples; 6 biological replicates per sample for vehicle, IFN-α 500 pg/mL and IFN-α 5000 pg/mL) was performed using the Robust MultiChip Average ANOVA statistical test.

**Pathway and Network Analysis**

IPA Software was used to identify regulation of molecular signalling pathways. Differential gene expression across treatment was assessed by applying a *P* value filter (for treatment) of *P* < .05 to the ANOVA results. To investigate the effect of different IFN-α concentrations, a 3 linear contrast was performed (IFN-α 500 pg/mL vs vehicle; IFN-α 5000 pg/mL vs vehicle; IFN-α 500 pg/mL vs IFN-α 5000 pg/ml). In this comparison, a maximum filter of *P* < .05 and a minimum absolute fold change cut-off of 1.2 was applied. Genes that passed these criteria were used to build up the Venn diagram (supplementary Figure 5a).

**qPCR Analysis and Validation**

Seventeen genes were measured using qPCR analyses as a validation step for the transcriptomics analysis, using both upregulated and downregulated genes: 5 genes modulated only by IFN-α 500 pg/mL, 5 genes modulated only by IFN-α 5000 pg/mL, and 7 genes modulated by both concentrations. mRNA gene expression levels were analyzed by TaqMan qRT-PCR instrument (CFX384 real time system, Bio-Rad) using the iScriptTM one-step RTPCR kit for probes (Bio-Rad) previously described (Anacker et al., 2013). Briefly, samples were run in 96-well formats in triplicate as multiplexed reactions and each target gene normalized to the expression of the housekeeping genes ribosomal protein L13A (RPL13A) and vimentin (VIM) as references. For each sample, 30 ng of RNA was added to the Real Time PCR Mix. Thermal cycling was initiated with an incubation at 50°C for 10 minutes (RNA retrotranscription), then 95°C for 5 minutes (TaqMan polymerase activation). After this, 39 cycles of PCR were performed, with each cycle consisting of heating samples at 95°C for 10 seconds to enable the melting process, then at 60°C for 30 seconds for annealing and extension. Relative target gene expression was calculated according to the 2(-Delta Delta C(T)) method.

**SUPPLEMENTARY RESULTS**

**IFN-α Modulates Signaling Networks Involved in Neurogenesis and Inflammation**

Seven networks were regulated only by the lowest concentration of IFN-α. Of particular relevance are network 1 and network 2 together with their molecules, involved respectively in cellular development (supplementary Figure 3a) and cellular movement (supplementary Figure 3b). The ubiquitin interaction motif containing (UIMC)1 gene belongs to the first network and is known to interact with BRCA1 protein, which is involved in regulating hippocampal neuronal differentiation and plasticity (Chen et al., 2005). Similarly, the gene dedicator of cytokinesis (DOCK) family of proteins, such as DOCK11, is known to be highly expressed in the CNS and to have a protective function against neurodegeneration (Namekata et al., 2014). In addition, DOCK11 also has a role in the immune system (Lin et al., 2006). Interestingly, in our microarray data, this gene was significantly downregulated (supplementary Table 1), therefore suggesting a role in mediating the detrimental effect on neurogenesis as well as in governing the responsiveness of the immune system upon stimulation with IFN-α.

In addition, 13 networks were regulated only by the highest concentration of IFN-α. Of particular note are network 2 (supplementary Figure 3c) and 8 (supplementary Figure 3d) with their molecules, associated, respectively, with developmental disorders and hereditary disorders. From the first network, the transcription factor TOX high mobility group box family member 3 (TOX3) gene, highly expressed during development, is known to be part of the CREB signalling pathway (Dittmer et al., 2011), to stimulate cell proliferation, and to suppress cell death (Sahu et al., 2016). In contrast, among the molecules belonging to the second network, the tripartite motif containing 2 (TRIM2) is relevant for its involvement in preventing hereditary disorders such as axonal neuropathy (Ylikallio et al., 2013), associated with axonal degeneration and loss of neuronal polarity in hippocampal neurons (Khazaei et al., 2011). Interestingly, both genes were significantly downregulated in our microarray data (supplementary Table 2), again suggesting a role in mediating the detrimental effect on neurogenesis as well as the increase in cell death.

Finally, both concentrations of IFN-α modulated 10 networks, including network 2 (supplementary Figure 3e) and 3 (supplementary Figure 3f) with their main molecules, linked, respectively, to the inflammatory response and cell signalling. Specifically, within the genes from the first network, 2'-5'-oligoadenylate synthetase 1 (OAS1) is known to be highly upregulated by IFN-α and to participate in the inflammatory cascade (Hakansson et al., 2004). Most interestingly, evidence has shown that OAS1 is often induced in the presence of the abelson tyrosine kinase (ABL1) protein (Hakansson et al., 2004), which has the ability to inhibit hippocampal neurogenesis (Schlatterer et al., 2012). Similarly, insulin like growth factor binding protein 5 (IGFBP5) gene is also able to regulate cell proliferation and differentiation; indeed, its suppression can significantly impair neuronal generation (Tanno et al., 2005). In our microarray results, OAS1 and IGFBP5 were, respectively, upregulated and downregulated by both concentrations of IFN-α (supplementary Table 3), thus again suggesting a role in mediating the detrimental effect on neurogenesis.

**REFERENCES**

Anacker C, Cattaneo A, Luoni A, Musaelyan K, Zunszain PA, Milanesi E, Rybka J, Berry A, Cirulli F, Thuret S, Price J, Riva MA, Gennarelli M, Pariante CM (2013) Glucocorticoid-related molecular signaling pathways regulating hippocampal neurogenesis. Neuropsychopharmacology 38:872–883.

Bolstad BM, Irizarry RA, Astrand M, Speed TP (2003) A comparison of normalization methods for high density oligonucleotide array data based on variance and bias. Bioinformatics 19:185–193.

Bruno R, Sacchi P, Ciappina V, Zochetti C, Patruno S, Maiocchi L, Filice G (2004) Viral dynamics and pharmacokinetics of peginterferon alpha-2a and peginterferon alpha-2b in naive patients with chronic hepatitis c: a randomized, controlled study. Antiviral Ther 9:491–497.

Chen J, Zacharek A, Zhang C, Jiang H, Li Y, Roberts C, Lu M, Kapke A, Chopp M (2005) Endothelial nitric oxide synthase regulates brain-derived neurotrophic factor expression and neurogenesis after stroke in mice. J Neuroscience 25:2366–2375.

Dittmer S, Kovacs Z, Yuan SH, Siszler G, Kogl M, Summer H, Geerts A, Golz S, Shioda T, Methner A (2011) TOX3 is a neuronal survival factor that induces transcription depending on the presence of CITED1 or phosphorylated CREB in the transcriptionally active complex. J Cell Sci 124:252–260.

Francois C, Descamps V, Brochot E, Bernard I, Canva V, Mathurin P, Castelain S, Duverlie G (2010) Relationship between the hepatitis C viral load and the serum interferon concentration during the first week of peginterferon-alpha-2b-ribavirin combination therapy. J Med Virol 82:1640–1646.

Hakansson P, Segal D, Lassen C, Gullberg U, Morse HC, 3rd, Fioretos T, Meltzer PS (2004) Identification of genes differentially regulated by the P210 BCR/ABL1 fusion oncogene using cDNA microarrays. Exp Hematol 32:476–482.

Irizarry RA, Bolstad BM, Collin F, Cope LM, Hobbs B, Speed TP (2003) Summaries of Affymetrix GeneChip probe level data. Nucleic Acids Res 31:e15.

Khazaei MR, Bunk EC, Hillje AL, Jahn HM, Riegler EM, Knoblich JA, Young P, Schwamborn JC (2011) The E3-ubiquitin ligase TRIM2 regulates neuronal polarization. J Neurochem 117:29–37.

Lin Q, Yang W, Baird D, Feng Q, Cerione RA (2006) Identification of a DOCK180-related guanine nucleotide exchange factor that is capable of mediating a positive feedback activation of Cdc42. J Biol Chem 281:35253–35262.

Namekata K, Kimura A, Kawamura K, Harada C, Harada T (2014) Dock GEFs and their therapeutic potential: neuroprotection and axon regeneration. Prog Ret Eye Res 43:1–16.

Pathak JL, Bakker AD, Verschueren P, Lems WF, Luyten FP, Klein-Nulend J, Bravenboer N (2015) CXCL8 and CCL20 Enhance Osteoclastogenesis via Modulation of Cytokine Production by Human Primary Osteoblasts. PloS One 10:e0131041.

Sahu SK, Fritz A, Tiwari N, Kovacs Z, Pouya A, Wullner V, Bora P, Schacht T, Baumgart J, Peron S, Berninger B, Tiwari VK, Methner A (2016) TOX3 regulates neural progenitor identity. Biochim Biophys Acta 1859:833–840.

Schlatterer SD, Suh HS, Conejero-Goldberg C, Chen S, Acker CM, Lee SC, Davies P (2012) Neuronal c-Abl activation leads to induction of cell cycle and interferon signaling pathways. J Neuroinflammation 9:208.

Tanno B, Cesi V, Vitali R, Sesti F, Giuffrida ML, Mancini C, Calabretta B, Raschella G (2005) Silencing of endogenous IGFBP-5 by micro RNA interference affects proliferation, apoptosis and differentiation of neuroblastoma cells. Cell Death Diff 12:213–223.

Ylikallio E, Poyhonen R, Zimon M, De Vriendt E, Hilander T, Paetau A, Jordanova A, Lonnqvist T, Tyynismaa H (2013) Deficiency of the E3 ubiquitin ligase TRIM2 in early-onset axonal neuropathy. Hum Mol Genet 22:2975–2983.

**Supplementary Figure Legends**

**Supplementary Figure** **1**. Representative immunostaining images of neurogenic and apoptotic markers in control and upon both concentrations of IFN-α. Cells were treated with IFN-α (500pg/ml and 5000pg/ml) for 3 days during proliferation, following by 7 days during differentiation. In both control (a) and upon both concentrations of IFN-α (b, c) mature neurons were detected by MAP2 (red) over the total number of cells DAPI (blue), whereas apoptotic cells were stained by CC3 (green) over the total number of cells DAPI (blue).

**Supplementary Figure 2.** Affymetrix gene expression microarray analysis and qPCR validation. Venn diagram indicating the number of genes regulated by IFN-α (500pg/ml and 5000pg/ml) (a). qPCR validation of fold changes for 17 genes regulated by IFN-α (b).

**Supplementary Figure 3.** Network analysis of genes modulated by IFN-α. Network 1, “Cellular Development” (a) and network 2, “Cellular Movement” (b) modulated by IFN-α 500pg/ml only. Network 2, “Developmental Disorder” (c) and network 8, “Hereditary Disorder” (d) modulated by IFN-α 5000pg/ml only. Network 2, “Inflammatory Response” (e) and network 3, “Cell Signalling” (f) commonly modulated by both concentrations of IFN-α.

**Supplementary Figure 4.** Main features of the IFN-α signalling pathway. IFN-α upregulates ISG15 and USP18 gene expression, and IL-6 protein expression via activation of STAT1-mediated mechanisms. Subsequently, ISG15 upregulates UBA7, UBE2L6 and HERC5 gene expression again via STAT1 activation, potentially leading to a decrease in neurogenesis. USP18 reduces neurogenesis, potentially via regulation of the Notch signalling pathway. Finally, IL-6 downregulates AQP4 gene expression again via STAT1 activation, potentially leading to an increase in apoptosis.
